# Hypofractionated breast radiotherapy for 1 week versus 3 weeks (FAST-Forward): 5-year efficacy and late normal tissue effects results from a multicentre, non-inferiority, randomised, phase 3 trial

**DOI:** 10.1016/S0140-6736(20)30932-6

**Published:** 2020-05-23

**Authors:** Adrian Murray Brunt, Joanne S Haviland, Duncan A Wheatley, Mark A Sydenham, Abdulla Alhasso, David J Bloomfield, Charlie Chan, Mark Churn, Susan Cleator, Charlotte E Coles, Andrew Goodman, Adrian Harnett, Penelope Hopwood, Anna M Kirby, Cliona C Kirwan, Carolyn Morris, Zohal Nabi, Elinor Sawyer, Navita Somaiah, Liba Stones, Isabel Syndikus, Judith M Bliss, John R Yarnold, FAST-Forward Trial Management Group, Abdulla Alhasso, Anne Armstrong, Judith Bliss, David Bloomfield, Jo Bowen, Murray Brunt, Charlie Chan, Hannah Chantler, Mark Churn, Susan Cleator, Charlotte Coles, Ellen Donovan, Andy Goodman, Susan Griffin, Jo Haviland, Penny Hopwood, Anna Kirby, Julie Kirk, Cliona Kirwan, Marjory MacLennan, Carolyn Morris, Zohal Nabi, Elinor Sawyer, Mark Sculphur, Judith Sinclair, Navita Somaiah, Liba Stones, Mark Sydenham, Isabel Syndikus, Jean Tremlett, Karen Venables, Duncan Wheatley, John Yarnold

**Affiliations:** a University Hospitals of North Midlands and University of Keele, Stoke on Trent, UK; b Clinical Trials and Statistics Unit, The Institute of Cancer Research, Sutton, London, UK; c Royal Cornwall Hospital, Treliske, Truro, UK; d Beatson West of Scotland Cancer Centre, Glasgow, UK; e Brighton and Sussex University Hospitals, Brighton, UK; f Nuffield Health Cheltenham Hospital, Cheltenham, UK; g Worcestershire Acute Hospitals NHS Trust, Worcester, UK; h Imperial Healthcare NHS Trust, London, UK; i University of Cambridge, Cambridge, UK; j Royal Devon and Exeter NHS Foundation Trust, Exeter, UK; k Torbay Hospital NHS Foundation Trust, Torquay, UK; l Norfolk and Norwich University Hospital, Norwich, UK; m The Royal Marsden NHS Foundation Trust and The Institute of Cancer Research, London, UK; n University of Manchester, Manchester, UK; o Independent Cancer Patients' Voice, London, UK; p Mount Vernon Cancer Centre, Northwood, UK; q King's College London, London, UK; r Clatterbridge Cancer Centre, Bebington, Wirral, UK

## Abstract

**Background:**

We aimed to identify a five-fraction schedule of adjuvant radiotherapy (radiation therapy) delivered in 1 week that is non-inferior in terms of local cancer control and is as safe as an international standard 15-fraction regimen after primary surgery for early breast cancer. Here, we present 5-year results of the FAST-Forward trial.

**Methods:**

FAST-Forward is a multicentre, phase 3, randomised, non-inferiority trial done at 97 hospitals (47 radiotherapy centres and 50 referring hospitals) in the UK. Patients aged at least 18 years with invasive carcinoma of the breast (pT1–3, pN0–1, M0) after breast conservation surgery or mastectomy were eligible. We randomly allocated patients to either 40 Gy in 15 fractions (over 3 weeks), 27 Gy in five fractions (over 1 week), or 26 Gy in five fractions (over 1 week) to the whole breast or chest wall. Allocation was not masked because of the nature of the intervention. The primary endpoint was ipsilateral breast tumour relapse; assuming a 2% 5-year incidence for 40 Gy, non-inferiority was predefined as ≤1·6% excess for five-fraction schedules (critical hazard ratio [HR] of 1·81). Normal tissue effects were assessed by clinicians, patients, and from photographs. This trial is registered at isrctn.com, ISRCTN19906132.

**Findings:**

Between Nov 24, 2011, and June 19, 2014, we recruited and obtained consent from 4096 patients from 97 UK centres, of whom 1361 were assigned to the 40 Gy schedule, 1367 to the 27 Gy schedule, and 1368 to the 26 Gy schedule. At a median follow-up of 71·5 months (IQR 71·3 to 71·7), the primary endpoint event occurred in 79 patients (31 in the 40 Gy group, 27 in the 27 Gy group, and 21 in the 26 Gy group); HRs versus 40 Gy in 15 fractions were 0·86 (95% CI 0·51 to 1·44) for 27 Gy in five fractions and 0·67 (0·38 to 1·16) for 26 Gy in five fractions. 5-year incidence of ipsilateral breast tumour relapse after 40 Gy was 2·1% (1·4 to 3·1); estimated absolute differences versus 40 Gy in 15 fractions were −0·3% (−1·0 to 0·9) for 27 Gy in five fractions (probability of incorrectly accepting an inferior five-fraction schedule: p=0·0022 *vs* 40 Gy in 15 fractions) and −0·7% (−1·3 to 0·3) for 26 Gy in five fractions (p=0·00019 *vs* 40 Gy in 15 fractions). At 5 years, any moderate or marked clinician-assessed normal tissue effects in the breast or chest wall was reported for 98 of 986 (9·9%) 40 Gy patients, 155 (15·4%) of 1005 27 Gy patients, and 121 of 1020 (11·9%) 26 Gy patients. Across all clinician assessments from 1–5 years, odds ratios versus 40 Gy in 15 fractions were 1·55 (95% CI 1·32 to 1·83, p<0·0001) for 27 Gy in five fractions and 1·12 (0·94 to 1·34, p=0·20) for 26 Gy in five fractions. Patient and photographic assessments showed higher normal tissue effect risk for 27 Gy versus 40 Gy but not for 26 Gy versus 40 Gy.

**Interpretation:**

26 Gy in five fractions over 1 week is non-inferior to the standard of 40 Gy in 15 fractions over 3 weeks for local tumour control, and is as safe in terms of normal tissue effects up to 5 years for patients prescribed adjuvant local radiotherapy after primary surgery for early-stage breast cancer.

**Funding:**

National Institute for Health Research Health Technology Assessment Programme.

## Introduction

The Early Breast Cancer Trialists' Collaborative Group systematic overview confirms that radiotherapy after primary surgery in women with early-stage cancers reduces locoregional cancer recurrence and breast cancer deaths, including patients with positive lymph nodes treated by mastectomy and axillary clearance.^1^, ^2^ For many decades, schedules of adjuvant radiotherapy for these patients delivered 25 fractions of 2 Gy over 5 weeks. Randomised controlled trials with long-term follow-up have since confirmed that fewer, larger fractions giving a lower total dose are at least as safe and effective as the previously used international standard.^3^, ^4^, ^5^, ^6^, ^7^, ^8^, ^9^, ^10^ Specifically, mature data confirm the safety and non-inferiority of 15 or 16 fractions of about 2·7 Gy to total doses of 40·0 Gy or 42·5 Gy.^5^, ^8^ A 3-week schedule of 15 fractions has been the UK standard of care for adjuvant locoregional radiotherapy for early breast cancer since 2009 and is now an international standard for adjuvant local radiotherapy.^11^, ^12^ There is no reason to assume that 15 fractions represent the lower limits of this hypofractionated and accelerated approach. We report outcomes of the FAST-Forward randomised phase 3 trial testing two dose levels of a five-fraction regimen delivered in 1 week against 40 Gy in 15 fractions over 3 weeks for patients prescribed local radiotherapy after breast conservation surgery or mastectomy for early breast cancer. The objectives are to identify a 1-week schedule non-inferior to a standard 3-week regimen for 5-year local tumour control and similar in terms of late adverse effects. FAST-Forward was informed by the FAST trial that tested two dose levels of five once-weekly fractions;^13^, ^14^ FAST trial results to 10-year follow-up are to be published soon. The trial design used dose levels estimated to be the upper and lower bounds that are isoeffective with the control schedule in terms of tumour control and normal tissue effects.

**Research in context**

**Evidence before this study**

We searched PubMed on April 22, 2020, using the search terms “breast cancer”, “adjuvant radiotherapy”, “hypofractionation”, and “randomised clinical trials”. We searched for primary research and reviews published in English between Jan 1, 1980, and April 22, 2020. We found 13 randomised studies testing adjuvant breast hypofractionated radiotherapy regimens against standard fractionation ranging in sample size from 30 to 2236 patients. All offered consistent support for the experimental approach.

Radiotherapy (radiation therapy) after primary surgery for early breast cancer has historically been delivered in five daily doses (fractions) of 1·8–2·0 Gy per week over at least 5 weeks, but randomised phase 3 clinical trials done in Canada, the UK, and subsequently, China and Denmark have confirmed the safety and efficacy of 15-fraction or 16-fraction schedules using daily fractions of 2·7 Gy. Four of these trials published 10-year follow-up data on a total of 7000 patients, and 3-week schedules have replaced traditional regimens in many countries over the past decade for most, if not all, patients prescribed local or locoregional radiation therapy after breast conservation surgery or mastectomy. Most recently, long-term outcome data for a five-fraction schedule delivered by once-weekly treatments has been reported, which suggests further scope for simplifying curative radiotherapy for women with early breast cancer.

**Added value of this study**

15 or 16 fractions over 3·0–3·2 weeks are unlikely to represent the limits of this approach, called hypofractionation. The FAST-Forward trial shows that 26 Gy in five fractions of 5·2 Gy to the conserved breast or post-mastectomy chest wall after primary surgery is non-inferior in terms of 5-year ipsilateral local tumour relapse to 40 Gy in 15 fractions over 3 weeks within an absolute 1·6% non-inferiority margin compared with 2% incidence with 40 Gy. The 5-day schedule causes milder early skin reaction and similar rates of late adverse effects. When mature, a randomised FAST-Forward substudy will report the safety of the five-fraction regimen for patients prescribed radiotherapy to breast or chest wall combined with axilla or supraclavicular fossa.

**Implications of all the available evidence**

FAST-Forward results confirm that 26 Gy in five fractions is as effective and safe as an international standard 15-fraction regimen after primary surgery for early breast cancer. The 1-week schedule has major benefits over the 3-week or 5-week regimens in terms of convenience and cost for patients and for health services globally.

## Methods

### Study design

FAST-Forward is a multicentre, non-blinded, phase 3, randomised, non-inferiority trial, done at 97 hospitals (47 radiotherapy centres and 50 referring hospitals) in the UK, testing the safety and efficacy of five-fraction schedules of adjuvant radiotherapy to the whole breast or chest wall delivered in 1 week compared with the UK standard 15-fraction 3-week schedule. Substudies included a published acute toxicity study,^15^ photographic assessments of late adverse effects, and patient-reported outcomes; not all centres participated in the substudies. Following recruitment into the main trial a further substudy opened, testing the same fractionation schedules for patients requiring radiotherapy to the axilla or supraclavicular fossa lymph nodes after sentinel node biopsy or supraclavicular fossa only (levels 3–4) after axillary dissection with a primary endpoint focusing on safety. Patients and results from this substudy are not reported here because follow-up is not yet mature. FAST-Forward was approved by the national South East Coast Kent research ethics committee (11/LO/0958) and local research and development offices of all participating centres. The trial protocol is in the appendix (pp 15–78).

### Patients

Eligible patients were women or men aged at least 18 years with invasive carcinoma of the breast (pT1–3, pN0–1, M0) following complete microscopic excision of the primary tumour by breast conservation surgery or mastectomy (reconstruction allowed), recruited in the UK from 47 radiotherapy centres and 50 referral centres. A protocol amendment on Feb 15, 2013, excluded the lowest-risk patients (aged ≥65 years, pT1, grade 1 or 2, oestrogen receptor [ER] positive, HER2 negative, pN0, M0) to increase the overall primary event rate. All patients had axillary surgery (sentinel node biopsy or axillary dissection); nodal radiotherapy was not allowed in the main study. Concurrent endocrine therapy or trastuzumab, or both, were permitted but not concurrent chemotherapy. For the patient-reported outcomes substudy all patients at participating centres were eligible. All patients who had breast conservation surgery were eligible for the photographic substudy at participating centres. A small number of patients who had had mastectomy were recruited into the photographic substudy to validate the scoring method in patients who had chest wall radiotherapy, but are not reported here because photographs were only available for 76 patients. All patients provided written informed consent.

### Randomisation and masking

Patients were randomly assigned (1:1:1) to receive either 40 Gy in 15 fractions of 2·67 Gy; 27 Gy in five fractions of 5·4 Gy; or 26 Gy in five fractions of 5·2 Gy. A sequential tumour bed radiotherapy boost to the conserved breast was allowed, with centres required to specify boost intention and dose (10 Gy or 16 Gy in 2-Gy fractions) before randomisation. Randomisation was done by telephone or fax from the recruiting centre to the Institute of Cancer Research-Clinical Trials and Statistics Unit (ICR-CTSU), Sutton, London, UK, and used an in-house bespoke trial-specific randomisation system set-up by the ICR-CTSU IT team. Computer-generated random permuted blocks were used (block sizes 6 and 9), stratified by radiotherapy centre and risk group (high [age <50 years or grade 3] *vs* low [age ≥50 years and grade 1 or 2]). Treatment allocation was not masked to clinicians or patients.

Test dose levels were informed by START^8^ and FAST^15^ trials generating α/β values for late normal tissue effects. Assuming an α/β value of 3 Gy and no effect of overall time on outcomes, 27 Gy in five fractions of 5·4 Gy was predicted to match late normal tissue effects of 40 Gy in 15 fractions of 2·7 Gy or 46 Gy in 23 fractions of 2 Gy. Allowance for a possible effect of treatment time informed the choice of the slightly lower 26 Gy dose level.

### Radiotherapy

The whole breast clinical target volume, including the soft tissues from 5 mm below the skin surface to the deep fascia, was either established from field-based tangential fields or the volume was contoured prospectively. Post-mastectomy chest wall clinical target volume encompassed post-surgical skin flaps and underlying soft tissues to the deep fascia; both excluded underlying muscle and rib cage. Surgeons were strongly encouraged to mark the tumour cavity walls with titanium clips or gold seeds at the time of breast conservation surgery in order to aid placement of tangential fields and delineation of tumour bed. A typical margin of 10 mm was added around the breast or chest wall clinical target volume accounting for set-up error, breast swelling, and breathing to create a planning target volume (PTV). For all patients, a full 3D CT set of outlines covering the whole breast and organs at risk was collected with a slice separation up to 5 mm, and organs at risk were outlined prospectively. A tangential opposing pair beam arrangement encompassed the whole breast or chest wall PTV, minimising the ipsilateral lung and heart exposure. The treatment plan was optimised with 3D dose compensation to achieve the following PTV dose distribution: more than 95% of PTV received 95% of prescribed dose, less than 5% of PTV received 105% or more, less than 2% of PTV received 107% or more, and a global maximum of less than 110%. Dose constraints for the control group were as follows: volume of ipsilateral lung receiving 12 Gy less than 15%, and volume of heart receiving 2 Gy less than 30% and that receiving 10 Gy less than 5%. Dose constraints for the five-fraction schedules were as follows: volume of ipsilateral lung receiving 8 Gy less than 15%, and volume of heart receiving 1·5 Gy less than 30% and that receiving 7 Gy less than 5%. X-ray beam energies for treatment were 6 MV or 10 MV, but a mixture of energies—eg, 6 MV and 10–15 MV—was allowed for larger patients, assessed on a case-by-case basis. Tumour bed boost was delivered via electrons or photons. Verification was done using electronic portal imaging using MV or kV x-rays. Control group treatment verification was required for at least three fractions in the first week with correction for any systematic error and then once weekly with a tolerance of 5 mm. The five-fraction schedules required verification imaging for each fraction with recommendations to correct all measured displacements. A comprehensive quality assurance programme involved every radiotherapy centre before trial activation and continued throughout trial accrual; this was coordinated by the UK Radiotherapy Trials Quality Assurance team based at Mount Vernon Hospital, Northwood, UK. The radiotherapy planning pack is in the appendix (pp 79–103).

### Assessments

Patients were assessed by clinicians for ipsilateral breast tumour relapse and late normal tissue effects at annual follow-up visits. Starting 12 months after trial entry, late-onset normal tissue effects in ipsilateral breast or chest wall (breast distortion, shrinkage, induration and telangiectasia; and breast or chest wall oedema and discomfort) were graded by clinicians on a four-point scale (none, a little, quite a bit, or very much), interpreted as none, mild, moderate, or marked. Symptomatic rib fracture, symptomatic lung fibrosis, and ischaemic heart disease were recorded. Clinical assessments of acute skin toxicity have been previously reported.^15^

In the patient-reported outcomes substudy, questionnaires were administered at baseline (before randomisation) and at 3, 6, 12, 24, and 60 months, including the European Organisation for Research and Treatment of Cancer QLQ-BR23 breast cancer module, body image scale, and protocol-specific questions relating to changes to the affected breast after treatment (including breast appearance changed, smaller, harder or firmer, and skin appearance changed). Patient assessments used a four-point scale (not at all, a little, quite a bit, and very much).

In the photographic substudy, photographs were taken at baseline and at 2 and 5 years after radiotherapy. Change in photographic breast appearance compared with baseline (after surgery and before radiotherapy) was scored on a three-point scale (none, mild, or marked) based on changes in breast size and shape relative to the contralateral breast. Patients were ineligible for further photographic assessments after breast reconstruction surgery and further ipsilateral disease. Digital photographs were scored by three observers who were masked to patient identity and treatment allocation, following scoring procedures established in the START trials.^16^ Breast size and surgical deficit were assessed from the baseline photographs on a three-point scale (small, medium, and large).

### Outcomes

The primary endpoint was ipsilateral breast tumour relapse, defined as invasive carcinoma or ductal carcinoma in situ presenting anywhere in the ipsilateral breast parenchyma or overlying skin or post-mastectomy chest wall, whether considered local recurrence or new primary tumour. Data on first regional relapse (axilla, supraclavicular fossa, and internal mammary chain), distant metastases, new primary cancer, and death were collected. Key secondary endpoints were late normal tissue effects assessed by clinicians, patients, and from photographs, and other disease-related and survival outcomes (locoregional relapse, distant relapse, disease-free survival, and overall survival; appendix p 29).

### Statistical analysis

The target sample size was 4000 patients (balanced allocation between groups). This provided 80% power (one-sided α of 0·025 allowing for non-inferiority hypothesis and a simple Bonferroni correction taking into account comparisons between each test schedule and the control group^17^) to exclude an absolute increase of 1·6% in 5-year ipsilateral breast tumour relapse incidence for a five-fraction schedule compared with control, assuming 2% 5-year incidence in the 40 Gy group (START data,^7^ and allowing for reduced ipsilateral breast tumour relapse due to evolution of surgical techniques and systemic therapy). The 1·6% absolute non-inferiority margin was defined at the trial design stage by the protocol development group, which included clinicians and patient advocates and was considered to be acceptable and appropriate. Binary proportions were used for the sample size calculations because event rates are so low. Estimates allowed for 10% loss to follow-up or unevaluable patients, expected to be largely due to development of metastatic disease. 2196 patients (732 per group) was estimated for the photographic and patient-reported outcomes substudies to provide 80% power to detect an 8% difference in the 5-year prevalence of late normal tissue effects between the five-fraction schedules (assuming 35% with 5-year mild or marked change in photographic breast appearance from START-B 40 Gy results^7^), allowing for 10% loss to follow-up or unevaluable patients.

Kaplan-Meier estimates (with 95% CIs) of 5-year ipsilateral breast tumour relapse incidence were calculated, and hazard ratios (HRs; with 95% CIs) comparing fractionation schedules obtained from Cox proportional hazards regression, censoring patients at date of death or last follow-up. Absolute differences (with 95% CIs) in 5-year ipsilateral breast tumour relapse incidence were estimated by applying the HRs (and 95% CIs) to the control group 5-year event-free estimate.^18^ Primary assessment of non-inferiority was based on whether the upper limit of the two-sided 95% CI (corresponding to one-sided 97·5% CI) for the absolute difference in 5-year ipsilateral breast tumour relapse was less than 1·6%. Non-inferiority of each five-fraction schedule versus control was also tested using the a priori critical HR of 1·81 (ln0·964/ln0·98, from protocol-specified incidence); p<0·025 was deemed statistically significant (probability of incorrectly accepting an inferior five-fraction schedule). An exploratory competing risks analysis was done for ipsilateral breast tumour relapse, with death from any cause as a competing event in a Fine–Gray competing risks regression model.

Clinician and patient assessments of late normal tissue effects were analysed as follows: (1) 5-year cross-sectional analyses compared prevalence of moderate or marked effects versus none or mild effects between groups using risk ratios and risk differences and Fisher's exact test; and (2) longitudinal analyses of moderate or marked effects (*vs* none or mild) using generalised estimating equations^19^ including all assessments, comparing groups across the whole follow-up period using odds ratios (ORs) and the Wald test. Generalised estimating equations models included a term for years of follow-up, enabling time trends to be modelled. Additionally, to enable comparison of clinician-assessed normal tissue effects with results reported from other trials, survival analysis methods analysed time to first moderate or marked event, including Kaplan-Meier estimates of cumulative incidence, and groups compared using HRs from Cox proportional hazards regression and the pairwise log-rank test.

Scores for change in photographic breast appearance at 2 and 5 years were modelled using generalised estimating equations. Categories of mild and marked change in photographic breast appearance were combined for analysis because very few had marked changes. Pairwise comparisons of mild or marked change at 2 or 5 years between groups were described by ORs obtained from the generalised estimating equations models and the Wald test. Because of multiple testing, a significance level of 0·005 was used for the clinician and patient normal tissue effects assessments; all hypotheses for the normal tissue effects endpoints were two-sided.

Estimates of fractionation sensitivity (α/β values) in FAST-Forward were obtained for the primary endpoint of ipsilateral breast tumour relapse and late normal tissue effects as per methods in the START and FAST trials.^3^ The α/β estimate for breast cancer was obtained from a Cox proportional hazards regression model of time to first ipsilateral breast tumour relapse, and for late normal tissue effects from generalised estimating equations models including all follow-up assessments (separate models for photographic and clinician assessments). Each model included terms for total dose and total dose multiplied by fraction size; the α/β ratio was calculated by dividing the two parameter estimates respectively, with a 95% CI estimated from the model using the covariance of the two estimates (lower confidence limits were truncated at zero). Isoeffect doses in 2 Gy equivalents (EQD_2_) were calculated for the five-fraction schedules, together with an estimate of the five-fraction schedule that would be isoeffective with 40 Gy in 15 fractions in terms of local tumour control and late normal tissue effects. No correction was made for difference in treatment time.

No formal interim analyses were done; accumulating data were monitored annually by the independent data monitoring committee. All analyses were performed on an intention-to-treat basis that included all patients according to their allocated treatment regardless of what was actually received. Because the main hypothesis was non-inferiority, the primary endpoint was also tested in the per-protocol population, which excluded patients for whom a major deviation was reported. The database snapshot was taken on Nov 22, 2019; Stata, version 15 (StataCorp), was used for analyses. The trial is registered at isrctn.com, ISRCTN19906132.

### Role of the funding source

The funder of the study had no role in study design, data collection, data analysis, data interpretation, or writing of the report. The corresponding author had full access to all data in the study and had final responsibility for the decision to submit for publication.

## Results

**Figure 1:**
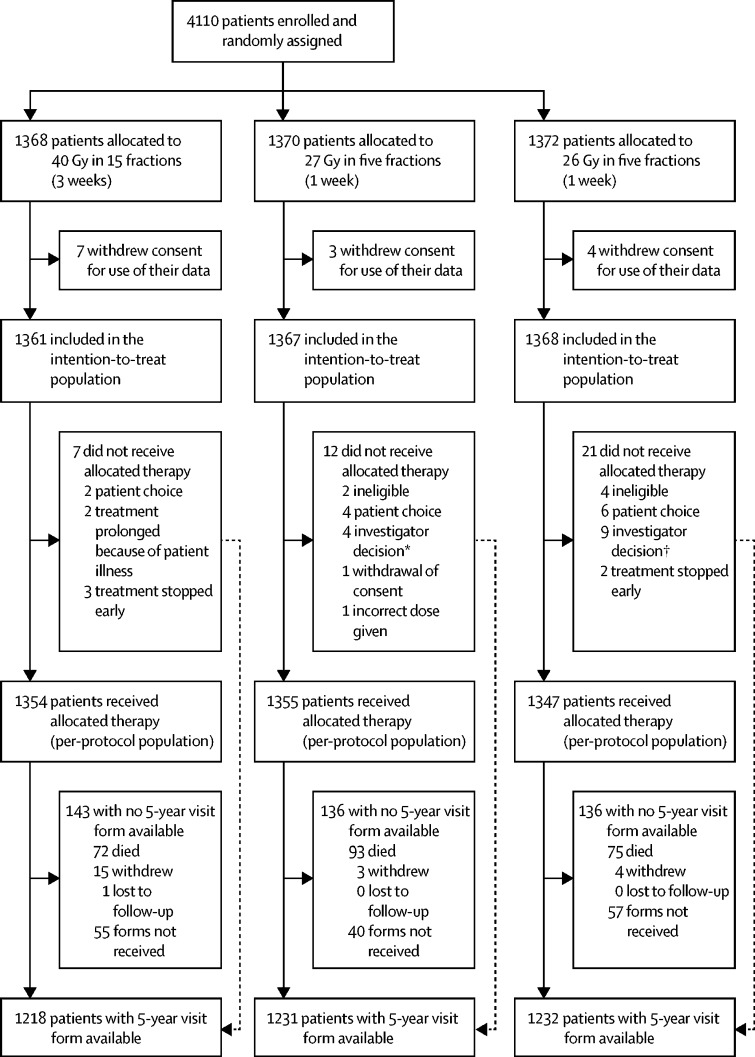
FAST-Forward trial profile *One patient had no radiotherapy as they were unable to get into a stable position; three were given 40 Gy in 15 fractions (one because of concern for brachial plexus, one decided on a different treatment plan, and one because of constraints of treatment planning). †One patient had no radiotherapy because they were diagnosed with pemphigoid and eight were given 40 Gy in 15 fractions (one because dose constraints were not met, one was unable to plan within protocol constraints because of tumour bed position, one had poor planning target volume coverage, one had technical difficulties in planning, one was transferred to direct electron field, one had a simulator plan because 3D images were not possible, one had a small pericardial effusion found at planning, and one gave no reason).

Between Nov 24, 2011, and June 19, 2014, 4110 patients were enrolled in the FAST-Forward trial. After randomisation, 14 patients withdrew consent for use of data and were removed from the intention-to-treat population; thus, 4096 patients were included in the intention-to-treat anlaysis (1361 assigned to 40 Gy in 15 fractions; 1367 assigned to 27 Gy in five fractions; and 1368 assigned to 26 Gy in five fractions; figure 1). Seven patients in the 40 Gy group, 12 in the 27 Gy group, and 21 in the 26 Gy group did not receive the allocated therapy and were not included in the per-protocol population. Compliance with allocated treatment was 99%. Demographic and clinical characteristics at baseline were well balanced between groups (table 1). 5-year visit forms were available for 3681 (96%) patients of 3833 still in follow-up (not died, withdrawn, or lost).

**Table 1:** Demographic, clinical, and treatment characteristics at randomisation (n=4096)*

				**40 Gy in 15 fractions (n=1361)**	**27 Gy in five fractions (n=1367)**	**26 Gy in five fractions (n=1368)**
Age, years						
	Median (IQR)			60 (53–66)	61 (53–67)	61 (52–66)
	Range			29–89	25–90	25–89
	<40			12 (0·9%)	16 (1·2%)	28 (2·0%)
	40–49			186 (13·7%)	173 (12·7%)	189 (13·8%)
	50–59			440 (32·3%)	423 (30·9%)	414 (30·3%)
	60–69			506 (37·2%)	511 (37·4%)	524 (38·3%)
	70–79			175 (12·9%)	197 (14·4%)	172 (12·6%)
	≥80			42 (3·1%)	47 (3·4%)	41 (3·0%)
Sex						
	Female			1355 (99·6%)	1365 (99·9%)	1362 (99·6%)
	Male			6 (0·4%)	2 (0·1%)	4 (0·3%)
	Unknown			0	0	2 (0·1%)
Tumour grade						
	1			315 (23·1%)	315 (23·0%)	300 (21·9%)
	2			660 (48·5%)	663 (48·5%)	690 (50·4%)
	3			386 (28·4%)	389 (28·5%)	378 (27·6%)
Risk group						
	Low (age ≥50 and grade 1 or 2)			843 (61·9%)	854 (62·5%)	854 (62·4%)
	High (age <50 or grade 3, or both)			518 (38·1%)	513 (37·5%)	514 (37·6%)
Primary surgery						
	Breast conservation surgery			1270 (93·3%)	1278 (93·5%)	1284 (93·9%)
			Breast conservation surgery with oncoplastic technique	42 (3·1%)	33 (2·4%)	42 (3·1%)
	Mastectomy			91 (6·7%)	89 (6·5%)	84 (6·1%)
		Mastectomy with immediate reconstruction		8 (0·6%)	11 (0·8%)	7 (0·5%)
			Autologous reconstruction	5/8 (62·5%)	7/11 (63·6%)	3/7 (42·9%)
			Implant-based reconstruction	2/8 (25·0%)	4/11 (27·3%)	4/7 (57·1%)
			Reconstruction type not specified	1/8 (12·5%)	0	0
Side of primary tumour						
	Left			726 (53·3%)	674 (49·3%)	662 (48·4%)
	Right			635 (46·7%)	693 (50·7%)	704 (51·5%)
	Unknown			0	0	2 (0·1%)
Maximal extent of axillary staging						
	Sentinel node biopsy or guided axillary sampling			1157 (85·0%)	1184 (86·6%)	1164 (85·1%)
	Axillary clearance			200 (14·7%)	181 (13·2%)	201 (14·7%)
	Other			4 (0·3%)	2 (0·1%)	1 (0·1%)
	Unknown			0	0	2 (0·1%)
Pathological node status						
	Positive			257 (18·9%)	243 (17·8%)	256 (18·7%)
	Negative			1103 (81·0%)	1124 (82·2%)	1110 (81·1%)
	Unknown			1 (0·1%)	0	2 (0·1%)
Histological type						
	Infiltrating ductal			1084 (79·6%)	1096 (80·2%)	1086 (79·4%)
	Lobular			144 (10·6%)	139 (10·2%)	127 (9·3%)
	Mixed			51 (3·7%)	63 (4·6%)	65 (4·8%)
	Other			82 (6·0%)	69 (5·0%)	87 (6·4%)
	Unknown			0	0	3 (0·2%)
Pathological tumour size, cm						
	Median (IQR)			1·6 (1·1–2·2)	1·6 (1–2·2)	1·6 (1·1–2·4)
Pathological T stage						
	T1mi			4 (0·3%)	5 (0·4%)	6 (0·4%)
	T1a			69 (5·1%)	68 (5·0%)	51 (3·7%)
	T1b			258 (19·0%)	270 (19·8%)	256 (18·7%)
	T1c			612 (45·0%)	601 (44·0%)	602 (44·0%)
	T2			394 (28·9%)	389 (28·5%)	424 (31·0%)
	T3			21 (1·5%)	30 (2·2%)	25 (1·8%)
	Unknown			3 (0·2%)	4 (0·3%)	4 (0·3%)
ER and HER2 status						
	ER positive HER2 positive			103 (7·6%)	103 (7·5%)	93 (6·8%)
	ER positive HER2 negative			1108 (81·4%)	1130 (82·7%)	1097 (80·2%)
	ER negative HER2 positive			32 (2·4%)	34 (2·5%)	42 (3·1%)
	ER negative HER2 negative			111 (8·2%)	96 (7·0%)	128 (9·4%)
	Not known			7 (0·5%)	4 (0·3%)	8 (0·6%)
Progesterone receptor status						
	Positive			577 (73·1%)†	541 (70·3%)†	566 (69·8%)†
	Negative			212 (26·9%)†	229 (29·7%)†	245 (30·2%)†
	Not done			571 (42·0%)	596 (43·6%)	555 (40·6%)
	Missing on form			1 (0·1%)	1 (0·1%)	2 (0·1%)
Lymphovascular invasion						
	Present			186 (13·7%)	178 (13·0%)	202 (14·8%)
	Absent			1085 (79·7%)	1084 (79·3%)	1055 (77·1%)
	Uncertain			34 (2·5%)	40 (2·9%)	51 (3·7%)
	Unknown			56 (4·1%)	65 (4·8%)	60 (4·4%)
Neoadjuvant chemotherapy received‡						
	Yes			48 (3·5%)	56 (4·1%)	43 (3·1%)
	No			1312 (96·4%)	1311 (95·9%)	1323 (96·7%)
	Unknown			1 (0·1%)	0	2 (0·1%)
Adjuvant therapy received: all patients						
	Chemotherapy§			333/1360 (24·5%)	324/1367 (23·7%)	370/1366 (27·1%)
Adjuvant therapy received:‡ HER2-positive patients						
	Chemotherapy and trastuzumab			84/135 (62·2%)	85/137 (62·0%)	100/135 (74·1%)
	Trastuzumab, no chemotherapy			16/135 (11·9%)	13/137 (9·5%)	13/135 (9·6%)
	Chemotherapy, no trastuzumab			2/135 (1·5%)	2/137 (1·5%)	0
	No chemotherapy, no trastuzumab			33/135 (24·4%)	37/137 (27·0%)	22/135 (16·3%)
Adjuvant therapy received:‡ ER-positive patients						
	Endocrine therapy			1169/1216 (96·1%)	1186/1237 (95·9%)	1157/1196 (96·7%)
Boost given						
	Yes			342 (25·1%)	337 (24·7%)	332 (24·3%)
	No			1017 (74·7%)	1027 (75·1%)	1031 (75·4%)
	Not known			2 (0·1%)	3 (0·2%)	5 (0·4%)
Boost dose						
	10 Gy in five fractions			260/342 (76·0%)	273/337 (81·0%)	257/332 (77·4%)
	16 Gy in eight fractions			80/342 (23·4%)	64/337 (19·0%)	75/332 (22·6%)
	Unknown			2/342 (0·6%)	0	0

Data are n (%) or n/N (%) unless otherwise stated. ER=oestrogen receptor.

* 14 patients withdrew consent for any of their data to be used in analysis.

† These percentages are calculated out of total patients who had available results for this test.

‡ Patients could have more than one type of adjuvant systemic therapy.

§ Chemotherapy type (for those specified): anthracyclines (n=584), taxane and anthracyclines (n=348), taxane and other—eg, docetaxel, carboplatin, and trastuzumab (n=83), and other (n=3).

**Figure 2:**
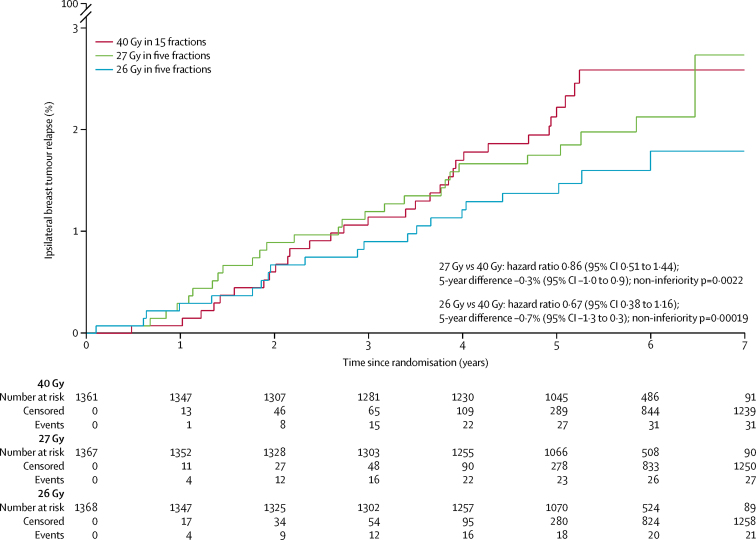
Cumulative risk of ipsilateral breast tumour relapse by fractionation schedule

After a median follow-up of 71·5 months (IQR 71·3 to 71·7), ipsilateral breast tumour relapse was recorded in 79 patients (31 in the 40 Gy group, 27 in the 27 Gy group, and 21 in the 26 Gy group); HRs versus 40 Gy in 15 fractions were 0·86 (95% CI 0·51 to 1·44) for 27 Gy in five fractions and 0·67 (0·38 to 1·16) for 26 Gy in five fractions. Estimated cumulative incidence of ipsilateral breast tumour relapse up to 5 years was 2·1% (95% CI 1·4 to 3·1) for 40 Gy (expected incidence 2%), 1·7% (1·2 to 2·6) for 27 Gy and 1·4% (0·9 to 2·2) for 26 Gy (table 2, figure 2). Estimated absolute differences in ipsilateral breast tumour relapse versus 40 Gy were −0·3% (−1·0 to 0·9) for 27 Gy and −0·7% (−1·3 to 0·3) for 26 Gy. The upper confidence limits excluded an increase in ipsilateral breast tumour relapse of 1·6% or more so non-inferiority can be claimed for both five-fraction schedules compared with 40 Gy in 15 fractions. A test against the critical HR greater than 1·81 confirmed the result, with a p value of 0·0022 for 27 Gy and 0·00019 for 26 Gy compared with 40 Gy. Analyses in the per-protocol population were consistent (estimated absolute difference *vs* 40 Gy −0·4% [–1·0 to 0·8], p=0·0017 for 27 Gy and −0·6% [–1·2 to 0·4], p=0·00037 for 26 Gy; full data for per-protocol analyses not shown because treatment compliance was 99%). Comparing the five-fraction schedules, the estimated absolute difference in ipsilateral breast tumour relapse cumulative incidence up to 5 years was −0·4% (−1·0 to 0·6) for 26 Gy versus 27 Gy. The unadjusted α/β estimate for ipsilateral breast tumour relapse was 3·7 Gy (0·3 to 7·1), with EQD_2_ estimates of 44·7 Gy for 40 Gy, 43·1 Gy for 27 Gy, and 40·6 Gy for 26 Gy with no correction for treatment time. Adjusting for risk group and ER and HER2 status made minimal difference (adjusted α/β estimate 3·7 Gy [95% CI 0·4 to 6·9]). HRs obtained from a competing risks analysis of ipsilateral breast tumour relapse with death from any cause as a competing event were almost identical to those from the primary analysis (HRs from competing risks model were 0·85 [95% CI 0·51 to 1·43] for 27 Gy *vs* 40 Gy; and 0·67 [0·38 to 1·16] for 26 Gy *vs* 40 Gy).

**Table 2:** Relapse and mortality by fractionation schedule: time-to-event analysis (n=4096)

	**Cumulative number of events**	**Estimated cumulative incidence by 5 years (95% CI)**	**Hazard ratio (95% CI); p value**	**Estimated absolute difference *vs* 40 Gy at 5 years (95% CI)**
Ipsilateral breast tumour (local) relapse*				
40 Gy (n=1361)	31 (2·3%)	2·1% (1·4 to 3·1)	1 (ref)	..
27 Gy (n=1367)	27 (2·0%)	1·7% (1·2 to 2·6)	0·86 (0·51 to 1·44); 0·56	−0·3% (−1·0 to 0·9)
26 Gy (n=1368)	21 (1·5%)	1·4% (0·9 to 2·2)	0·67 (0·38 to 1·16); 0·15	−0·7% (−1·3 to 0·3)
**Locoregional relapse**†				
40 Gy (n=1361)	43 (3·2%)	2·8% (2·0 to 3·9)	1 (ref)	..
27 Gy (n=1367)	35 (2·6%)	2·3% (1·6 to 3·3)	0·80 (0·51 to 1·25); 0·33	−0·5% (−1·4 to 0·7)
26 Gy (n=1368)	29 (2·1%)	1·8% (1·2 to 2·7)	0·66 (0·41 to 1·06); 0·083	−0·9% (−1·6 to 0·2)
**Distant relapse**				
40 Gy (n=1361)	59 (4·3%)	3·8% (2·9 to 5·0)	1 (ref)	..
27 Gy (n=1367)	69 (5·0%)	4·7% (3·7 to 6·0)	1·16 (0·82 to 1·64); 0·41	0·6% (−0·7 to 2·3)
26 Gy (n=1368)	76 (5·6%)	5·1% (4·0 to 6·4)	1·27 (0·90 to 1·79); 0·17	1·0% (−0·4 to 2·9)
**Any breast cancer-related event**‡				
40 Gy (n=1361)	119 (8·7%)	7·8% (6·5 to 9·4)	1 (ref)	..
27 Gy (n=1367)	112 (8·2%)	7·2% (5·9 to 8·7)	0·93 (0·71 to 1·20); 0·56	−0·6% (−2·2 to 1·5)
26 Gy (n=1368)	114 (8·3%)	7·5% (6·2 to 9·0)	0·94 (0·73 to 1·22); 0·65	−0·4% (−2·1 to 1·6)
**All-cause mortality**				
40 Gy (n=1361)	92 (6·8%)	5·4% (4·3 to 6·8)	1 (ref)	..
27 Gy (n=1367)	105 (7·7%)	6·9% (5·7 to 8·4)	1·12 (0·85 to 1·48); 0·42	0·6% (−0·8 to 2·5)
26 Gy (n=1368)	90 (6·6%)	5·6% (4·5 to 7·0)	0·96 (0·72 to 1·28); 0·78	−0·2% (−1·5 to 1·5)

Hazard ratios less than 1 favour five-fraction schedules. p values were calculated by log-rank test (two-sided).

* Includes three patients with angiosarcoma in ipsilateral breast (one in the 40 Gy group and two in the 26 Gy group).

† Defined as ipsilateral breast tumour relapse or regional relapse (axilla, supraclavicular fossa, and internal mammary chain).

‡ Includes local, regional, or distant relapse, breast cancer death, or contralateral breast cancer (disease-free survival).

Regional relapses occurred in 34 (0·8%) of 4096 patients (13 [1·0%] of 1361 in the 40 Gy group, 11 [0·8%] in the 27 Gy group, and ten [0·7%] in the 26 Gy group; table 3), six of which were concurrent with ipsilateral breast tumour relapse. Incidence of locoregional relapse, distant relapse, disease-free survival, and overall survival were similar between groups, with no statistically significant differences (table 2; appendix pp 3–4). No formal subgroup analyses were done because of the low number of primary endpoint events, but frequencies of ipsilateral breast tumour relapse, regional relapse, and distant relapse were tabulated according to age, grade, and ER and HER2 status for descriptive purposes; as expected, ipsilateral breast tumour relapse occurred in more of the patients with higher-grade primary tumours (appendix p 7). Invasive contralateral breast cancer was reported for 55 (1·3%) of 4096 patients (18 [1·3%] in the 40 Gy group, 17 [1·2%] in the 27 Gy group, and 20 [1·5%] in the 26 Gy group; table 3), and non-breast second primary cancers were reported for 123 (3·0%) of 4096 patients (42 [3·1%] in the 40 Gy group, 37 [2·7%] in the 27 Gy group, and 44 [3·2%] in the 26 Gy group; table 3), the most common being colorectal cancer (25 cases).

**Table 3:** Relapses, second primary cancers, and deaths by fractionation schedule (n=4096)

		**40 Gy in 15 fractions (n=1361)**	**27 Gy in five fractions (n=1367)**	**26 Gy in five fractions (n=1368)**
Local tumour control event (primary endpoint)*		31 (2·3%)	27 (2·0%)	21 (1·5%)
	Local relapse	23 (1·7%)	22 (1·6%)	17 (1·2%)
	Ipsilateral breast, new primary	6 (0·4%)	3 (0·2%)	4 (0·3%)
	Cannot differentiate	2 (0·1%)	2 (0·1%)	0
Regional relapse		13 (1·0%)	11 (0·8%)	10 (0·7%)
Distant relapse		59 (4·3%)	69 (5·0%)	76 (5·5%)
Contralateral breast, second primary		23 (1·7%)	20 (1·5%)	23 (1·7%)
	Invasive	18 (1·3%)	17 (1·2%)	20 (1·5%)
	Ductal carcinoma in situ	5 (0·4%)	3 (0·2%)	2 (0·1%)
	Unknown	0	0	1 (0·1%)
Non-breast, second primary		42 (3·1%)	37 (2·7%)	44 (3·2%)
Death		92 (6·8%)	105 (7·7%)	90 (6·6%)
	Breast cancer†	47 (3·5%)	51 (3·7%)	53 (3·9%)
	Second cancer	12 (0·9%)	16 (1·2%)	10 (0·7%)
	Cardiac	10 (0·7%)	9 (0·7%)	8 (0·6%)
	Other cause	17 (1·7%)	27 (2·0%)	16 (1·2%)
	Unknown	6 (1·2%)	2 (0·1%)	3 (0·2%)

Data are n (%). Patients reporting events of more than one type are included in each relevant row.

* Includes angiosarcoma in ipsilateral breast (one in the 40 Gy group and two in the 26 Gy group) and six patients with ductal carcinoma in situ (three in the 40 Gy group, two in the 27 Gy group, and one in the 26 Gy group).

† Includes 13 patients with distant relapse before death from other causes (four in the 40 Gy group, four in the 27 Gy group, and five in the 26 Gy group).

287 (7·0%) of 4096 patients died, 151 (3·7%) from breast cancer, 125 (3·1%) from other causes, and 11 (0·3%) with unknown cause of death and no evidence of disease relapse (table 3). Of 27 patients with a cardiac-related death, 15 had a history of cardiac disease reported at randomisation or were a current or ex-smoker in the past year.

At least one annual clinical assessment of normal tissue effects was available for 3975 (97·0%) of 4096 patients. At 5 years, any moderate or marked clinician-assessed normal tissue effects in the breast or chest wall was reported for 98 of 986 (9·9%) 40 Gy patients, 155 (15·4%) of 1005 27 Gy patients, and 121 of 1020 (11·9%) 26 Gy patients (appendix pp 5–6, 8–9), with a significant difference between 40 Gy and 27 Gy (p=0·0003) but not between 40 Gy and 26 Gy (p=0·17). Breast shrinkage was the most prevalent moderate or marked effect at 5 years, reported in 50 (5·5%) of 916 40 Gy patients, 78 (8·2%) of 948 27 Gy patients, and 65 (6·8%) of 954 26 Gy patients (appendix pp 8–9). Longitudinal analysis of all annual clinical assessments of normal tissue effects over follow-up showed a significantly increased risk of any moderate or marked effect in the breast or chest wall for the 27 Gy group compared with 40 Gy (OR 1·55 [95% CI 1·32 to 1·83], p<0·0001), with no significant difference between 26 Gy and 40 Gy (1·12 [0·94 to 1·34], p=0·20; table 4). This pattern was similar for the individual effects of breast distortion, shrinkage, induration, and breast or chest wall oedema, with significantly higher risk for 27 Gy than 40 Gy but not for 26 Gy (table 4; appendix pp 5–6). Comparing the two five-fraction schedules, 26 Gy had significantly lower risk of any moderate or marked breast or chest wall normal tissue effects (p=0·0001) and breast shrinkage (p=0·0018) compared with 27 Gy. Estimates of 5-year cumulative incidence of any moderate or marked clinician-assessed normal tissue effects in the breast or chest wall were 26·8% (95% CI 24·4 to 29·4) for 40 Gy, 35·1% (32·4 to 37·9) for 27 Gy, and 28·5% (26·0 to 31·1) for 26 Gy (appendix p 10). Results for comparison of schedules from the analyses of time to first moderate or marked effect were similar to those from the longitudinal modelling of all annual clinical assessments (appendix p 10).

**Table 4:** Longitudinal analysis of moderate or marked clinician-assessed late normal tissue effects for patients with at least one annual clinical assessment (n=3975)

		**Number of moderate or marked events/total number of assessments over follow-up**	**Odds ratio for schedule (95% CI)**	**p value for comparison with 40 Gy**	**p value for comparison between 27 Gy and 26 Gy**	**Odds ratio for years of follow-up (95% CI); p value**
Any adverse event in the breast or chest wall*		..	..	..	..	0·98 (0·96–1·00); 0·055
	40 Gy	651/6121 (10·6%)	1 (ref)	..	..	..
	27 Gy	1004/6303 (15·9%)	1·55 (1·32–1·83)	<0·0001	..	..
	26 Gy	774/6327 (12·2%)	1·12 (0·94–1·34)	0·20	0·0001	..
**Breast distortion**†		..	..	..	..	0·99 (0·95–1·02); 0·38
	40 Gy	232/5724 (4·0%)	1 (ref)	..	..	..
	27 Gy	363/5953 (6·1%)	1·51 (1·15–1·97)	0·0028	..	..
	26 Gy	299/5945 (5·0%)	1·20 (0·91–1·60)	0·19	0·083	..
**Breast shrinkage**†		..	..	..	..	1·03 (1·00–1·06); 0·023
	40 Gy	330/5728 (5·8%)	1 (ref)	..	..	..
	27 Gy	503/5944 (8·5%)	1·50 (1·20–1·88)	0·0004	..	..
	26 Gy	369/5943 (6·2%)	1·05 (0·82–1·33)	0·71	0·0018	..
**Breast induration (tumour bed)**†		..	..	..	..	1·00 (0·96–1·04); 0·95
	40 Gy	185/5713 (3·2%)	1 (ref)	..	..	..
	27 Gy	304/5948 (5·1%)	1·56 (1·19–2·05)	0·0013	..	..
	26 Gy	236/5937 (4·0%)	1·19 (0·90–1·59)	0·23	0·047	..
**Breast induration (outside tumour bed)**†		..	..	..	..	0·96 (0·90–1·02); 0·17
	40 Gy	45/5712 (0·8%)	1 (ref)	..	..	..
	27 Gy	137/5943 (2·3%)	2·79 (1·74–4·50)	<0·0001	..	..
	26 Gy	97/5930 (1·6%)	1·90 (1·15–3·14)	0·013	0·059	..
**Telangiectasia**		..	..	..	..	1·21 (1·14–1·29); <0·0001
	40 Gy	63/6087 (1·0%)	1 (ref)	..	..	..
	27 Gy	100/6272 (1·6%)	1·68 (1·07–2·65)	0·025	..	..
	26 Gy	102/6300 (1·6%)	1·53 (0·96–2·43)	0·070	0·65	
**Breast or chest wall oedema**		..	..	..	..	0·73 (0·69–0·78); <0·0001
	40 Gy	89/6097 (1·5%)	1 (ref)	..	..	..
	27 Gy	217/6287 (3·4%)	2·18 (1·57–3·03)	<0·0001	..	..
	26 Gy	155/6318 (2·4%)	1·47 (1·03–2·09)	0·032	0·0097	..
**Breast or chest wall discomfort**		..	..	..	..	0·93 (0·89–0·97); 0·0003
	40 Gy	234/6086 (3·8%)	1 (ref)	..	..	..
	27 Gy	269/6285 (4·3%)	1·10 (0·86–1·40)	0·44	..	..
	26 Gy	250/6309 (4·0%)	0·98 (0·76–1·26)	0·86	0·35	..

Results for years of follow-up show trend in normal tissue effects over follow-up across all fractionation schedules. p values are calculated by Wald test; odds ratios are estimated from the generalised estimating equations model including all follow-up data and show relative odds of moderate or marked adverse event (*vs* none or mild) for each pairwise comparison of fractionation schedules across all follow-up assessments.

* Includes shrinkage, induration, telangiectasia, or oedema.

† Patients who had breast conservation surgery or mastectomy with reconstruction.

1796 patients consented to the patient-reported outcomes substudy, 18 of whom withdrew consent immediately after randomisation or were not given the baseline booklet. Questionnaires returned from those expected (patients alive and well, not withdrawn) were 1771 (99·6%) of 1778 at baseline, 1668 (96·2%) of 1733 at 3 months, 1622 (94·2%) of 1722 at 6 months, 1599 (93·7%) of 1707 at 1 year, 1531 (91·7%) of 1669 at 2 years, and 1334 (84·0%) of 1589 at 5 years. Of the 1774 patients with at least one completed questionnaire, 1634 had breast conservation surgery and 140 had mastectomy. Change in breast appearance had the highest 5-year prevalence, with moderate or marked change reported in 140 (32·4%) of 432 for 40 Gy, 158 (35·9%) of 440 for 27 Gy, and 136 (31·7%) of 429 for 26 Gy. 5-year prevalence of patient-reported adverse effects were not significantly different between the schedules (appendix pp 5–6, 11–12). Patient-reported moderate or marked breast hardness or firmness at 5 years was not significantly increased for 27 Gy compared with 40 Gy and breast swelling was not more prevalent in both five-fraction schedules than the 40 Gy schedule, at the prespecified cutoff of p=0·005. Longitudinal analyses of all patient assessments from baseline to 5 years showed a significantly higher risk of moderate or marked breast hardness or firmness for 27 Gy compared with 40 Gy (OR 1·42, 1·17, 1·72, p=0·0003), and a lower risk of change in breast appearance for 26 Gy compared with 27 Gy (p=0·0018), but no significant differences between schedules for the other normal tissue effects (table 5; appendix pp 5–6).

**Table 5:** Longitudinal analysis of moderate or marked patient-assessed late normal tissue effects from baseline to 5 years for patients with at least one completed questionnaire (n=1774)

		Number of patients reporting moderate or marked event at baseline/total*	**Number of moderate or marked events/total number of assessments over 3–60 months follow-up**	**Odds ratio for schedule (95% CI)**	**p value for comparison with 40 Gy**	**p value for comparison between 27 Gy and 26 Gy**	**Odds ratio for years of follow-up (95% CI); p value**
**Protocol-specific items**							
Breast appearance changed		..	..	..	..	..	1·03 (1·01–1·05); 0·0010
	40 Gy	170/573 (29·7%)	778/2480 (31·4%)	1 (ref)	..	..	..
	27 Gy	177/583 (30·4%)	929/2550 (36·4%)	1·22 (1·02–1·46)	0·033	..	..
	26 Gy	155/581 (26·7%)	770/2563 (30·0%)	0·91 (0·75–1·10)	0·33	0·0018	..
Breast smaller		..	..	..	..	..	1·11 (1·09–1·13); <0·0001
	40 Gy	96/560 (17·1%)	585/2445 (23·9%)	1 (ref)	..	..	..
	27 Gy	106/576 (18·4%)	606/2520 (24·0%)	1·05 (0·85–1·29)	0·67	..	..
	26 Gy	90/574 (15·7%)	515/2542 (20·3%)	0·81 (0·65–1·00)	0·053	0·017	..
Breast harder or firmer		..	..	..	..		0·95 (0·93–0·97); <0·0001
	40 Gy	94/558 (16·8%)	499/2446 (20·4%)	1 (ref)	..	..	..
	27 Gy	105/572 (18·4%)	690/2512 (27·5%)	1·42 (1·17–1·72)	0·0003	..	..
	26 Gy	95/566 (16·8%)	626/2534 (24·7%)	1·22 (1·00–1·48)	0·048	0·1007	..
Skin appearance changed		..	..	..	..	..	0·96 (0·93–0·99); 0·0080
	40 Gy	78/577 (13·5%)	345/2505 (13·8%)	1 (ref)	..	..	..
	27 Gy	61/586 (10·4%)	392/2571 (15·2%)	1·03 (0·83–1·28)	0·77	..	..
	26 Gy	67/580 (11·5%)	338/2576 (13·1%)	0·90 (0·72–1·13)	0·37	0·23	..
**European Organisation for Research and Treatment of Cancer QLQ-BR23 items**							
Breast pain		..	..	..	..	..	0·96 (0·94–0·99); 0·011
	40 Gy	53/583 (9·1%)	338/2538 (13·3%)	1 (ref)	..	..	..
	27 Gy	42/590 (7·1%)	428/2601 (16·5%)	1·23 (0·98–1·54)	0·068	..	..
	26 Gy	53/588 (9·0%)	417/2597 (16·1%)	1·23 (0·98–1·53)	0·074	0·96	..
Breast swollen		..	..	..	..	..	0·84 (0·80–0·89); <0·0001
	40 Gy	56/583 (9·6%)	122/2538 (4·8%)	1 (ref)	..	..	..
	27 Gy	43/589 (7·3%)	236/2597 (9·1%)	1·46 (1·10–1·94)	0·0080	..	..
	26 Gy	47/589 (8·0%)	192/2599 (7·4%)	1·27 (0·95–1·69)	0·11	0·22	..
Breast oversensitive		..	..	..	..	..	0·96 (0·93–0·99); 0·0097
	40 Gy	57/579 (9·8%)	283/2528 (11·2%)	1 (ref)	..	..	..
	27 Gy	42/584 (7·2%)	334/2596 (12·9%)	1·10 (0·87–1·40)	0·43	..	..
	26 Gy	62/586 (10·6%)	319/2587 (12·3%)	1·11 (0·88–1·41)	0·37	0·91	..
Skin problems in breast		..	..	..	..	..	0·96 (0·92–1·01); 0·11
	40 Gy	26/582 (4·5%)	156/2539 (6·1%)	1 (ref)	..	..	..
	27 Gy	24/290 (4·1%)	209/2596 (8·0%)	1·25 (0·95–1·65)	0·11	..	..
	26 Gy	18/590 (3·0%)	164/2592 (6·3%)	0·98 (0·73–1·31)	0·90	0·084	..
Arm or shoulder pain		..	..	..	..	..	1·00 (0·97–1·03); >0·99
	40 Gy	66/582 (11·3%)	401/2537 (15·8%)	1 (ref)	..	..	..
	27 Gy	78/591 (13·2%)	441/2601 (17·0%)	1·12 (0·91–1·37)	0·29	..	..
	26 Gy	81/589 (13·7%)	455/2599 (17·5%)	1·14 (0·93–1·40)	0·2006	0·83	..
Arm or hand swollen		..	..	..	..	..	1·06 (1·00–1·11); 0·031
	40 Gy	24/582 (4·1%)	101/2536 (4·0%)	1 (ref)	..	..	..
	27 Gy	17/588 (2·9%)	103/2600 (4·0%)	0·95 (0·66–1·36)	0·77	..	..
	26 Gy	22/590 (3·7%)	124/2592 (4·8%)	1·14 (0·80–1·62)	0·46	0·31	..
Difficulty raising arm		..	..	..	..	..	1·04 (0·99–1·08); 0·089
	40 Gy	27/582 (4·6%)	171/2533 (6·7%)	1 (ref)	..	..	..
	27 Gy	36/589 (6·1%)	209/2599 (8·0%)	1·24 (0·94–1·63)	0·12	..	..
	26 Gy	37/587 (6·3%)	188/2596 (7·2%)	1·12 (0·85–1·48)	0·42	0·46	..

Results for years of follow-up show trend in normal tissue effects over follow-up across all fractionation schedules. p values are calculated by Wald test; odds ratios are estimated from the generalised estimating equations model including all questionnaires (baseline to 5 years) and show relative odds of moderate or marked adverse events (*vs* none or mild) for each pairwise comparison of fractionation schedules across all questionnaires.

* Total is those who completed the corresponding question.

Of the 1737 patients who consented to the photographic substudy, baseline photographs were received for 1634 (94·1%), and 2-year or 5-year photographs were available for 1385 (79·7%). 1309 (75·4%) were patients who had breast conservation surgery; for these patients, 2-year photographs were assessed in 1267 and 5-year photographs were assessed in 875 (appendix p 13). 226 patients died or withdrew from the photographic substudy by year 5; for the remainder, the most common reasons for photographs not being taken were appointments not made because of clerical errors at the centres, patients not attending clinic visits, and patients withdrawing consent from the substudy. At 2 years, mild or marked change in photographic breast appearance was reported in 35 (8·5%) of 411 for 40 Gy, 67 (15·6%) of 429 for 27 Gy, and 46 (10·8%) of 427 for 26 Gy; corresponding results at 5 years were 34 (12·0%) of 283 for 40 Gy, 83 (26·9%) of 308 for 27 Gy, and 37 (13·0%) of 284 for 26 Gy (appendix p 13). Modelling 2-year and 5-year photographic assessments together, 27 Gy had a significantly increased risk of mild or marked change in breast appearance compared with 40 Gy (OR 2·29 [95% CI 1·60 to 3·27], p<0·0001), with no significant difference between 26 Gy and 40 Gy (OR 1·26 [0·85 to 1·86], p=0·24; appendix p 13). 26 Gy had a significantly lower risk of change in photographic breast appearance than 27 Gy (p=0·0006).

The unadjusted α/β estimate for any moderate or marked clinician-assessed normal tissue effects in the breast or chest wall was 1·7 Gy (95% CI 1·2 to 2·3), giving EQD_2_ estimates of 47·1 Gy for 40 Gy in 15 fractions, 51·6 Gy for 27 Gy in 5 fractions, and 48·3 Gy for 26 Gy in 5 fractions; adjusting for prognostic factors (age, boost, and whole-breast planning treatment volume as a proxy for breast size) made very little difference. The α/β estimated from the photographic endpoint (adjusting for breast size and surgical deficit assessed from the baseline photographs) was very similar (1·8 Gy [1·1 to 2·4]). The unadjusted α/β estimate for patient-reported change in breast appearance was 2·3 Gy (1·8 to 2·9), resulting in EQD_2_ estimates of 46·1 Gy for 40 Gy, 48·2 Gy for 27 Gy, and 45·2 Gy for 26 Gy; adjusting for covariates made minimal difference.

The most common specialist referral for radiotherapy-related adverse effects during follow-up was to lymphoedema clinics (appendix p 14). Incidence of ischaemic heart disease, symptomatic rib fracture, and symptomatic lung fibrosis was very low at this stage of follow-up (appendix p 14).

## Discussion

We demonstrated non-inferiority, measured in terms of ipsilateral breast tumour relapse, of 27 Gy and 26 Gy five-fraction schedules compared with 40 Gy in 15 fractions at 5 years' follow-up for patients with early breast cancer, most of whom were treated by local tumour excision and sentinel node biopsy for node-negative disease. Normal tissue effects up to 5 years for the 26 Gy in five fractions schedule were similar to those with the 40 Gy in 15 fractions schedule. Low rates of ipsilateral breast tumour relapse and of moderate or marked late normal tissue effects can be attributed to improvements in all diagnostic and treatment modalities and to the commitment of patients to early diagnosis and randomised trials.^20^

The 10-year analyses of ipsilateral breast tumour relapse and normal tissue effects reported by earlier Canadian^5^ and UK trials^3^, ^4^, ^6^, ^7^, ^8^ confirm that although normal tissue effects continue to accumulate beyond 5 years, there is evidence that relative differences between test and control groups change very little over time.^21^ In the START-B trial, the HR for clinician-assessed breast shrinkage after 40 Gy in 15 fractions compared with 50 Gy in 25 fractions was 0·83 (95% CI 0·66–1·04) at 5 years and 0·80 (0·67–0·96) at 10 years, by which time the proportion of patients with breast shrinkage increased from 11·4% (9·5–13·6) at 5 years to 26·2% (23·2–29·6) at 10 years.^8^ The findings of FAST-Forward can be applied to different prognostic groups in view of the very low overall ipsilateral breast tumour relapse incidence, a conclusion consistent with a meta-analysis of the 5861 patients entered into the three START trials, which identified no inconsistency of effect in terms of normal tissue effects or recurrence risk across any of the prognostic or treatment subgroups investigated.^8^

The absence of a detectable dose response for local tumour control between 26 Gy and 27 Gy in five fractions is a potential limit to precision, but this feature reflects the shallowness of the dose response curve for subclinical breast cancer that is around the 98% local tumour control level, so the −0·4% estimated difference in absolute levels of ipsilateral breast tumour relapse between 27 Gy and 26 Gy probably reflects random sampling variability in the ipsilateral breast tumour relapse rate or chance imbalances in unmeasured prognostic factors between test groups. For late normal tissue effects, the dose response is much steeper, enabling detection of clinically and statistically significant differences in event rates between 26 Gy and 27 Gy in five fractions. The five-fraction schedule isoeffective with 40 Gy in 15 fractions allows direct estimation of α/β for late normal tissue effects, which is consistent with values generated from our other trials. The α/β value of 3·7 Gy (95% CI 0·3–7·1) for tumour control in FAST-Forward is similar to the 3·5 Gy (1·2–5·7) estimated from the START pilot and START-A trials.^8^ Point estimates of α/β values, assuming no effect of time, for late normal tissue effects in FAST-Forward scored by clinicians, patients, and photographic assessments are closer to 2 Gy than the 3 Gy estimated in the earlier START^8^ and FAST trials,^14^ but 95% CIs overlap for each endpoint in all trials. In FAST, 915 women were randomly assigned after breast conservation surgery for node negative disease to 50 Gy in 25 fractions versus two dose levels of a five-fraction regimen delivered once weekly, thereby ensuring complete repair between fractions and controlling for overall treatment time.^13^, ^14^ The α/β value for change in photographic breast appearance in FAST was 2·6 Gy (1·4–3·7). Uncertainty about biological processes, which include a time factor in FAST-Forward, does not interfere with clinical evaluation and decisions on implementation of FAST-Forward results in similar patient groups.

The five-fraction regimen is relevant to partial-breast radiotherapy, the preferred alternative to whole-breast radiotherapy for many women after recent phase 3 trials.^22^, ^23^, ^24^, ^25^ Beyond its safety and effectiveness, the 26 Gy FAST-Forward schedule is convenient and substantially less expensive for patients and for health services. It is also likely to be safe for patients requiring regional radiotherapy, an approach that is under formal assessment in a randomised FAST-Forward substudy comparing 40 Gy in 15 fractions and 26 Gy in five fractions. Assuming no effect of time, 26 Gy in five fractions is equivalent to 46·8 Gy and 53·7 Gy in 2-Gy fractions assuming α/β values of 2 Gy and 1 Gy, respectively, dose intensities well within the limits of tolerance for these structures.^26^, ^27^ In terms of limitations of our study, there is no reason to consider the heart more sensitive to fraction size than most other soft tissues. It is undoubtedly sensitive to total dose but the tiny number of cardiac events in FAST-Forward prevents meaningful analysis.^28^ Any heart exposure is potentially harmful even after 2 Gy fractions, so the priority is to exclude the heart from the treatment volume as far as possible using deep inspiration breath hold or a similar technique,^29^, ^30^ or partial breast radiotherapy.^23^ The size of the trial prevents reliable subgroup analyses by patient age, tumour grade, receptor status, and systemic therapies, but consistent with our 10-year analyses of almost 6000 patients in the START hypofractionation trials, there is no suggestion of heterogeneity.^8^ Finally, synchronous boost regimens were avoided despite current interest in this application of hypofractionation. It is safer to consider boost as an independent treatment variable such as tumour grade or adjuvant systemic therapy whose impacts are randomly distributed across treatment groups. This allows a pure assessment of whole-breast hypofractionation without having to consider the partial volume effects of different breast dose levels. Routine implementation of 26 Gy in five fractions can more naturally incorporate appropriate five-fraction synchronous boost regimens.

In conclusion, 5-year ipsilateral breast tumour relapse incidence after a 1-week course of adjuvant breast radiotherapy delivered in five fractions is non-inferior to the standard 3-week schedule according to the predefined inferiority threshold. The 26 Gy dose level is similar to 40 Gy in 15 fractions in terms of patient-assessed normal tissue effects, clinician-assessed normal tissue effects, and photographic change in breast appearance, and is similar to normal tissue effects expected after 46–48 Gy in 2 Gy fractions. The consistency of FAST-Forward results with earlier hypofractionation trials supports the adoption of 26 Gy in five daily fractions as a new standard for women with operable breast cancer requiring adjuvant radiotherapy to partial or whole breast.

## Data sharing

Deidentified individual participant data, together with a data dictionary defining each field in the set, will be made available to other researchers on request. Trial documentation including the protocol are available online. The Institute of Cancer Research-Clinical Trials and Statistics Unit (ICR-CTSU) supports wider dissemination of information from the research it conducts and increased cooperation between investigators. Trial data are obtained, managed, stored, shared, and archived according to ICR-CTSU standard operating procedures to ensure the enduring quality, integrity, and utility of the data. Formal requests for data sharing are considered in line with ICR-CTSU procedures, with due regard given to funder and sponsor guidelines. Requests are via a standard proforma describing the nature of the proposed research and extent of data requirements. Data recipients are required to enter a formal data sharing agreement, which describes the conditions for release and requirements for data transfer, storage, archiving, publication, and intellectual property. Requests are reviewed by the trial management group in terms of scientific merit and ethical considerations, including patients' consent. Data sharing is undertaken if proposed projects have a sound scientific or patients' benefit rationale, as agreed by the trial management group and approved by the independent data monitoring and steering committee, as required. Restrictions relating to patients' confidentiality and consent will be limited by aggregating and anonymising identifiable patients' data. Additionally, all indirect identifiers that could lead to deductive disclosures will be removed in line with ICR-CTSU data sharing guidelines.
