# Supplementary material for: Hypofractionated breast radiotherapy for 1 week versus 3 weeks (FAST-Forward): 5-year efficacy and late normal tissue effects results from a multicentre, non-inferiority, randomised, phase 3 trial
Source: Lancet. 2020 May 23;395(10237):1613–26. doi: 10.1016/S0140-6736(20)30932-6 (PMC7262592; doi:10.1016/S0140-6736(20)30932-6)
Supplement: Supplementary appendix [file mmc1.pdf]

THE LANCET

Supplementary appendix

This appendix formed part of the original submission and has been peer reviewed. We post it as supplied by the authors.

Supplement to: Murray Brunt A, Haviland JS, Wheatley DA, et al. Hypofractionated breast radiotherapy for 1 week versus 3 weeks (FAST-Forward): 5-year efficacy and late normal tissue effects results from a multicentre, non-inferiority, randomised, phase 3 trial. *Lancet* 2020; published online April 28. http://dx.doi.org/10.1016/S0140-6736(20)30932-6.

Supplementary Appendix

Current principal investigators, past principal investigators, main co-investigators and trials teams (including physicists, radiographers, nurses and data managers). Number of patients recruited in brackets; current PI in bold; past PI/PIs in bold italics.
*** Indicates member of Trial Management Group**

Addenbrookes Hospital (99), **Prof C Coles***, Dr L Hughes-Davies, ***Dr C Wilson,*** A Bates, C Philpott, C Spain, B Stratton, N Twyman, J Wilkinson; Alexandra Hospital, Redditch (12), **Dr M Churn***, Dr D Hrouda, Dr C Irwin, H Hodson, A Morgan; Basildon Hospital (25), **Dr H Swinburn**, ***Dr W Ella***, C McCormick; Beatson West of Scotland Cancer Centre (24), **Dr A Al-Hasso***, Prof P Canney, Dr G Fraser, Dr G Lumsden, Dr M Rizwanullah, A Armstrong, J Fleming, T Ibotoye, A Leonard, M McJury, C Seager, V Withers; Bedford Hospital (2), **Dr S Smith,** V Bastion, G Lubimbi, A Willis, J Valentine; Belfast City Hospital (9), **Dr H McCarty**, Dr J Clarke, ***Dr G Hanna***, Dr L Mulholland, D Irvine, O Stewart, G Totton; Blackpool Victoria Infirmary (38), **Dr F Danwata**, ***Dr A Hindley***, Dr S Susnerwala, E Davies, S Lancaster, L Smith; Borders General Hospital (5), **Dr C Bedi**, M Tolson; Bristol Haematology and Oncology (47), **Dr C Comins**, ***Dr A Bahl***, Dr S Masson, Dr N Thanvi, H Appleby, A Lowe, H Saldanha, V Lee; Charing Cross Hospital (62), **Dr S Cleator***, ***Dr C Lowdell***, P Dunn, S McInerney; Cheltenham General Hospital (48), **Dr J Bowen***, Dr K Benstead, Dr R Counsell, Dr S Elyan, R Bakawala, N Bulmer, J Chittock, J Kukielska, A Skelton, M Tan; Churchill Hospital, Oxford (1), **Dr S Oliveros**, ***Dr B Lavery***, N Ann, C Hector, M Flavin, G Samkange; City Hospital. Birmingham (31), **Dr D Spooner**, D Devonport, B Gammon; Clatterbridge Cancer Centre (22), **Dr S Tolan**, ***Dr I Syndikus****, Dr N Thorp, S Green, K Hughes, H Mayles; Countess of Chester (12), **Dr A Hall**, S Bennett, E Gallimore, M Moffit, J Prince; Crosshouse Hospital, Kilmarmock (30), **Dr G Lumsden**, K Bain, C Burns, P Cannon; Derriford Hospital, Plymouth (144), **Dr U Panwar**, Dr S Dubey, ***Dr S Kelly*** (RIP), N Blacker, L Cadmore, H Congdon, I Harvey; Dumfries and Galloway Hospital (3), **Dr A Hennessy**, ***Dr T Evans***, J Duignan; Ealing Hospital (7), **Dr O Hatcher**, ***Dr C Lewanski***, S Magwaro, D Murati, K Watson; Eastbourne Hospital (35), **Dr S Westwell**, K Jones-Skipper; Essex County Hospital (19), **Dr M Mukesh**, Dr V Loo, ***Dr P Murray***, K Cooke, C Driscoll; Forth Valley Hospital (13), **Dr H Marashi**, Dr I Rabnawaz, F Johnston, L Prentice, A Scott; George Eliot Hospital, Nuneaton (35), **Dr S Lupton**, Dr L Fresco, J Lake; Glan Clwyd Hospital (31), **Dr J Bishop,** G Davies, S Owen, V Saul; Guys Hospital (25), **Prof E Sawyer***, Dr L Brazil, Dr S Harris, Prof A Tutt, G Keunzig, C Thomas; Hairmyres Hospital (1), **Dr J Hicks**, ***Dr G Dunn***, L Devlin, L Glass; Hereford County Hospital (18), **Dr D Nelmes**, ***Dr S Guglani***, J Birch, M Evans, G Horsfield; Hinchingbrooke Hospital (17), **Dr S Russell**, V Goss, R Kurian, S Miller; Ipswich Hospital (18), **Dr R Venkitaraman**, ***Dr L Sherwin***, C Mackenzie, P Ridley, M Riley; James Cook University Hospital (22), **Dr B Sethugavalar**, ***Dr J Hardman***, Dr JCM Van der Voet, H Curtis, E Thompson; James Paget Hospital (33), **Dr S Down**, ***Dr A Harnett****, A Brooks, J Harman; Kidderminster Hospital (47), **Dr M Churn***, S Stringer, H Tranter; Leicester Royal Infirmary (5), **Dr K Kancherla**, C Belcher, J Potterton, S Wright; Lincoln County Hospital (50), **Dr A Chaudhuri**, ***Dr E Murray***, Dr T Sreenivasan, O Francis, V Longdon, A Sloan; Luton and Dunstable University Hospital (23), **Dr A Vinayan**, ***Dr M Ah-See***, A Rafiq, M Sarte; Manor Hospital, Walsall (6), **Dr S Yaha**, ***Dr MS Anwar***, J Fletcher; Mount Vernon Hospital (108), **Dr C Westbury**, ***Dr M Ah-See***, Dr A Makris, Dr P Ostler, Dr N Shah, Dr N Thanvi, T Chalk, S Hasan, E Windmill; Musgrove Park, Taunton (39), **Dr M Varughese**, ***Dr J Graham***, S Mahoney; New Cross Hospital (67), **Dr R Allerton**, Dr C Brammer, ***Dr M Churn****, Dr L Pettit, Dr P Ramachandran, R Horton, M James-King; New Victoria ACH (7), **Dr D Ritchie**, Dr J Ansari, E Moody; Norfolk and Norwich Hospital (100), **Dr D Geropantas**, Dr A Bulman, ***Dr A Harnett****, S Barber, M Bloomfield, E Malone, J Platt; North Devon District Hospital (46), **Dr J Forrest**, ***Dr D Hwang***, R Holbrook, S Ley, L Van Koutrik; Northampton General Hospital (19), **Dr R Agrawal**, Prof H Eldeeb, ***Dr C Macmillan***, A Kempa, M Polnik, R Tighe, N Whilde; Peterborough Hospital (36), **Dr C Jephcott**, Dr C Round, Dr S Treece, K Cavanagh, C Chisenga, M Cowen; Pilgrim Hospital, Boston (9), **Dr A Chaudhuri**, ***Dr E Murray***, A Kirkby; Poole Hospital (8), **Dr J Brady**, Dr A Chakrabarti, Dr P Crellin, N Gurung, D Forster, F Mellor, B Troke, L Varnham; Queen Alexandra Hospital, Portsmouth (92), **Dr K Bradley**, Dr JD Dubois, Dr A Suovuori, R Baker, K Haselip, N Rivington; Queen Elizabeth Hospital, Birmingham (66), **Dr A Stevens**, ***Dr MS Anwar***, Dr D Spooner, S Manolopoulos, R Shingler; Queen Elizabeth Hospital, Kings Lynn (5), **Dr M Daly**, R Lee, H Webb; Queen Margaret Hospital, Dunfermline (4), **Dr M MacLennan***, Dr A Stillie, ***Dr T Evans***, F Adam; Queens Hospital, Romford (52), **Dr E Sims**, Dr C Bridgewater, ***Dr M Quigley***, Dr E Staples, T Mills-Baldock, J Cook; Royal Alexandra Hospital (41), **Dr A Al-Hasso***, P Eaddy, E MacLeod; Royal Berkshire (91), **Dr R Davis**, Dr J Adams, ***Dr J Barrett***, Dr C Charlton, J Jones, P Pabari, E Vowell; Royal Cornwall (248), **Dr D Wheatley***, Prof D Radstone, Dr A Thomson, n Ashley, S Eloi, A Griffiths, J Kingston, N Simpson; Royal Devon + Exeter Hospital (70), **Dr J Forrest**, Dr A Goodman*, ***Dr A Hong***, Dr D Hwang, K Baines, A Betts, T Lawless, S Scrutton; Royal Hampshire County Hospital (10), **Dr S Raj**, J Conti, V Corner, J Smith; Royal Lancaster Infirmary (92), **Dr D Williamson**, ***Dr G Skailes***, C Bartlett, A Fielding; Royal Marsden Hospital Sutton (185), **Dr N Somaiah***, Dr A Kirby*, Dr I Locke, Dr D Tait, ***Prof J Yarnold****, R Colgan, L Gothard, C Lucy; Royal Shrewsbury Hospital (126),

Dr L Pettit, ***Dr R Agrawal***, Dr H Abel Gadir, ***Dr S Khanduri***, S Jose, S Potts, A Welsh; Royal Surrey County Hospital (40), **Dr R Laing**, Dr A Franklin, Dr A Neal, Dr S Whittaker, L Adams, M Flavin, B Moloney-Oates, A Tindall; Royal Sussex County Hospital (116), **Dr D Bloomfield***, Dr S Mitra, Dr A Nikapota, Dr R Simcock, Dr S Westwell, P Frattaroli, A Hewines, J Tremlett; Royal United Hospital, Bath (45), **Dr M Beresford**, Dr A Jenner, Dr S Mancero, Dr S Manson, Dr H Newman, T Allen, C Milsom, T Tylee, S Whittle; Russell's Hall (21), **Dr R Allerton**, Dr G Georgiev, Dr P Ramachandra, K Kanyi, K McGarry, A Watts; Salisbury Hospital (21), **Dr C Crowley**, J Attlee, S Strong-Sheldrake; Sandwell Hospital (4), **Dr D Spooner**, D Devonport; Singleton Hospital, Swansea (72), **Dr M Rolles**, Dr C Askill, ***Dr D Pudney***, Dr R Taylor, E Brinkworth, H Cheley, S Foyle, E Harris, N Viney, J Williams; Southampton General Hospital (50), **Dr C Crowley**, Dr J Marshall, Dr S Raj, C Britton, E Cooper, K Meeking, K Stevens; Southend University Hospital (37), **Dr H Algurafi**, Dr W Ella, ***Dr A Robinson***, T Davies, A McPherson, L Romero, S Shibu-Thomas; St Bartholomew's Hospital (55), **Dr V Wolstenholme**, Dr C Cottrill, Dr N Patel, Dr K Tipples, F Bibi, H Payne, A Pena-Remorin, A Sivajothi, E Tutor; St Helens + Whiston (4), **Dr R Sripadam**, N Hornby; St James' University Hospital, Leeds (24), **Dr S Kumar**, Dr I Chaudhuri, S Hartup, A Henson, J Lilley, P Shuttleworth; St John's Hospital, Livingstone (7), **Dr F Yuille**, R Allen, A Clark; Stafford General Hospital (87), **Dr A Jegannathen**, Dr L Pettit, Dr C Brammer, C Harvey, A Myatt, L Verueco; Torbay District General Hospital (62), **Dr A Goodman***, Dr P Bliss, M Allison, P Bowen, S Chamberlain, I Koehler; University College London Hospitals (16), **Dr G Blackman**, Dr A Cassoni, Dr M Gaze, ***Dr M McCormack***, Dr J Tobias, R Patel, S Wickers; University Hospital of Coventry and Warwickshire (40), **Dr D Hrouda**, Dr L Fresco, Dr C Irwin, ***Dr N Walji***, Dr J Wordling, S Manolopoulos, K Sanders; University Hospital of North Staffordshire (298), **Prof AM Brunt***, Dr D Gahir, Dr A Jegannathen, L Contoret, M Evans, K Glover, A Myatt, R Smith; University Hospital of North Tees (8), **Dr E Thompson**, ***Dr N Storey***, Dr A Rathmell, L Poole; University Hospital, Aintree (8), **Dr P Robson**, L Beresford; Velindre Cancer Centre (167), **Dr H Passant***, Dr J Abraham, ***Prof P Barrett-Lee***, Dr A Borley, Dr T Howe, Dr R Stevens, M Jenkins, S Slade, A Weaver, O Woodley; Warrington Hospital (27), **Dr I Syndikus***, L Lee, C Lowthian, R Madew; Warwick Hospital (56), **Dr N Walji**, J Harris, L Maher; West Middlesex Hospital (17), **Dr P Riddle**, Dr R Ahmad, J Swallow; West Suffolk Hospital (10), **Dr M Moody**, Dr C Woodward, S Hale; Western General Hospital, Edinburgh (13), **Dr C Bedi**, Dr T Evans, Prof I Kunkler, Dr F Yuille, R Allan, L Carruthers, L Primrose; Weston General Hospital (22), **Dr T Wells**, ***Dr M Tomlinson***, K Owens, H Lloyd-Jones, G Saunders; Weston Park Hospital, Sheffield (86), **Dr M Hatton**, Dr O Din, Dr C Lee, G Brown, J Conway, L Fiorentino, J Swinscoe; Wexham Park Hospital (12), **Dr R Davis**, ***Dr J Adams***, Dr S Inayat, N Barnes, S Das, D Mciver, J Weerasinghe; Wishaw Hospital (25), **Dr J Hicks**, ***Dr M Rizwanullah***, K Douglas; Worcester Royal Infirmary (19), **Dr M Churn***, ***Dr J Bowen****, Dr R Counsell, D Bak, A Holdsworth, J Tyler; Worthing Hospital (59), **Dr SY Sham**, ***Dr A Nikapota***, ***Dr S Mitra***, S Funnell, J Gilbert; Wrexham Maelor Hospital (24), **Dr W Soe**, Dr J Bishop, Dr N Ghosal, J Stockport; Yeovil District Hospital (8), **Dr U Barthakur**, J McCrory, K Rennie; Ysbyty Gwynedd Hospital (10), **Dr J Bishop**, D Thomas, L Williams.

Non-recruiting centres: Royal Preston Hospital, **Dr M Hogg**, A Glendinning, R Hall; Pinderfields Hospital, Wakefield, **Dr S Kumar** , S Buckley; Ninewells Hospital, Dundee, **Douglas Adamson**, A Black, S Souliman, S Unit; Nottingham City Hospital, **Patricia Lawton**, S Dennis, S Fleet, E Harron; Kings Mill Hospital, Sutton-in-Ashfield, **Matthew Griffin**, D Nash.

Other Trial Management Group members not listed above: Dr A Alhasso, A Armstrong, Dr J Bowen, H Chantler, Dr E Donovan, Dr S Griffin, J Kirk, Dr M MacLennan, C Rawlings, Prof M Sculpher, J Sinclair, J Tremlett, Dr K Venables, M Wilcox, (past members: Dr R Agrawal, Dr J Barrett, Prof P Barrett-Lee, Dr P Bliss, Ms L Ciurlionis, Professor J Dewar, Dr P Dyson, Dr S Guglani, Dr H Mayles, D Megias, Dr H Passant, Dr A Robinson, Dr E Staples, R Zotova)

Institute of Cancer Research - Clinical Trials and Statistics Unit staff: J Adkins, S Atkins, J Barnett, L Colby, L Courtney, G Dower, M Emson, C Griffin, J Haviland, R Kaggwa, J Kidd, L Lloyd, K Mertens, J Mills, J Morden, A Obabumoye, D Patel, J Prince, S Simmons, E Simms, L Stones, G Sumo, M Sydenham, J Bliss

Radiotherapy Quality Assurance team: D Eaton, D Megias, Z Nabi, R Simoes, Y Tsang, K Venables, R Zotova

Independent Trial Steering Committee members: Professor M Mason (Chair), Dr D Gilbert, Dr V Cosgrove, Professor P Poortmans, Professor D Sebag-Montefiore, (past members Dr J Barrett, Professor S Bentzen)

Independent Data Monitoring Committee members: Professor M Sydes (Chair), Professor S Bentzen, Professor J Staffurth, (past member Professor I Turesson)

Figure A1a: Disease-free survival, by fractionation schedule

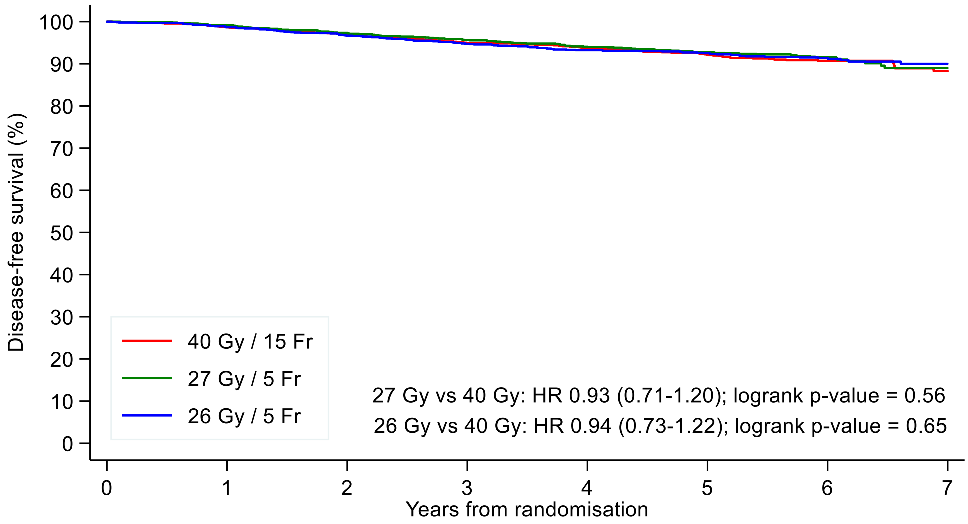

No. at risk (40Gy)	1361	1335	1292	1253	1204	1016	475	87
censored	0	8	29	40	74	242	771	1155
event	0	18	40	68	83	103	115	119
No. at risk (27Gy)	1367	1348	1313	1277	1229	1038	493	88
censored	0	7	18	31	58	233	768	1167
event	0	12	36	59	80	96	106	112
No. at risk (26Gy)	1368	1336	1305	1270	1220	1044	513	89
censored	0	14	18	27	57	224	745	1165
event	0	18	45	71	91	100	110	114

Figure A1b: Cumulative risk of any breast cancer-related event*, by fractionation schedule

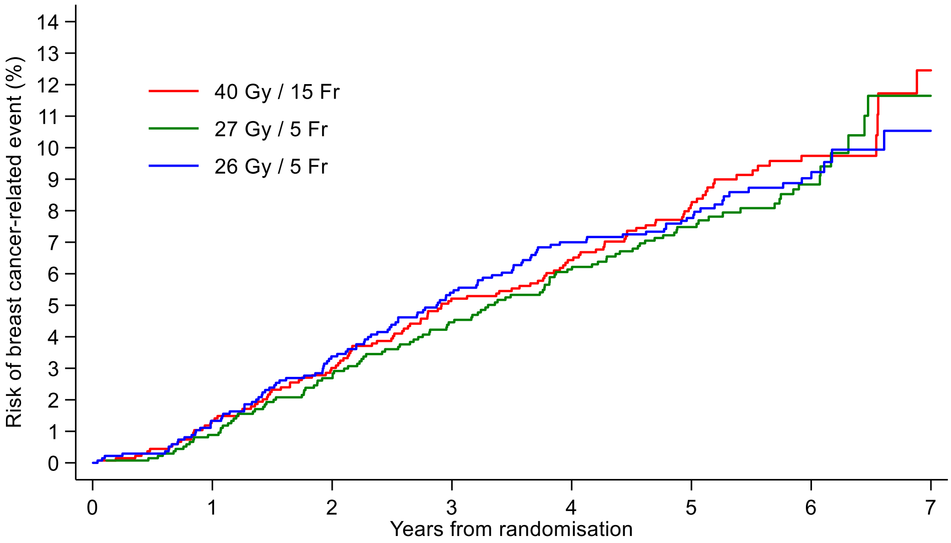

No. at risk (40Gy)	1361	1335	1292	1253	1204	1016	475	87
censored	0	8	29	40	74	242	771	1155
event	0	18	40	68	83	103	115	119
No. at risk (27Gy)	1367	1348	1313	1277	1229	1038	493	88
censored	0	7	18	31	58	233	768	1167
event	0	12	36	59	80	96	106	112
No. at risk (26Gy)	1368	1336	1305	1270	1220	1044	513	89
censored	0	14	18	27	57	224	745	1165
event	0	18	45	71	91	100	110	114

* Any breast cancer-related event includes local, regional or distant relapse, breast cancer death, contralateral breast cancer

Figure A2a: Overall survival, by fractionation schedule

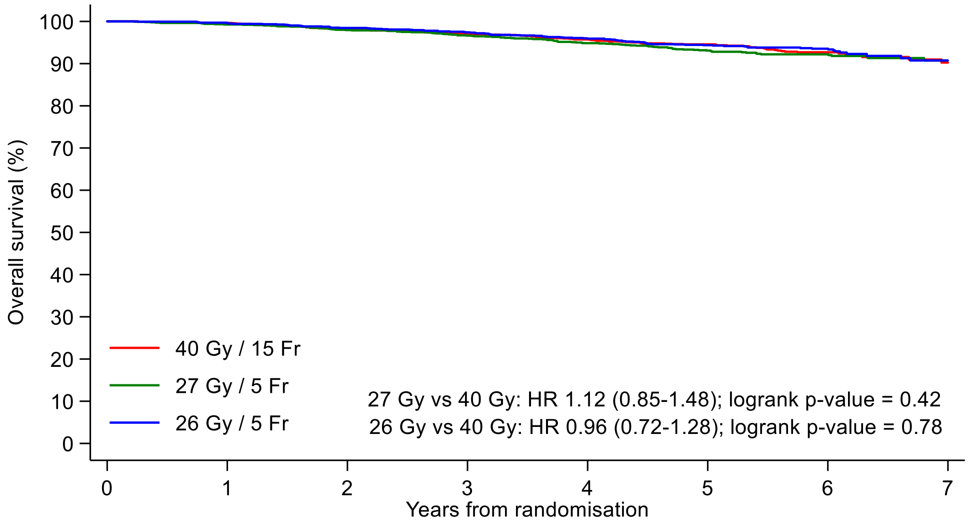

No. at risk (40Gy)	1361	1348	1312	1291	1245	1061	494	91
censored	0	8	22	31	59	228	780	1178
event	0	5	27	39	57	72	87	92
No. at risk (27Gy)	1367	1356	1337	1314	1271	1075	511	91
censored	0	1	2	7	26	199	755	1171
event	0	10	28	46	70	93	101	105
No. at risk (26Gy)	1368	1351	1332	1310	1266	1077	531	90
censored	0	12	15	23	47	216	755	1188
event	0	5	21	35	55	75	82	90

Figure A2b: Cumulative risk of death from any cause, by fractionation schedule

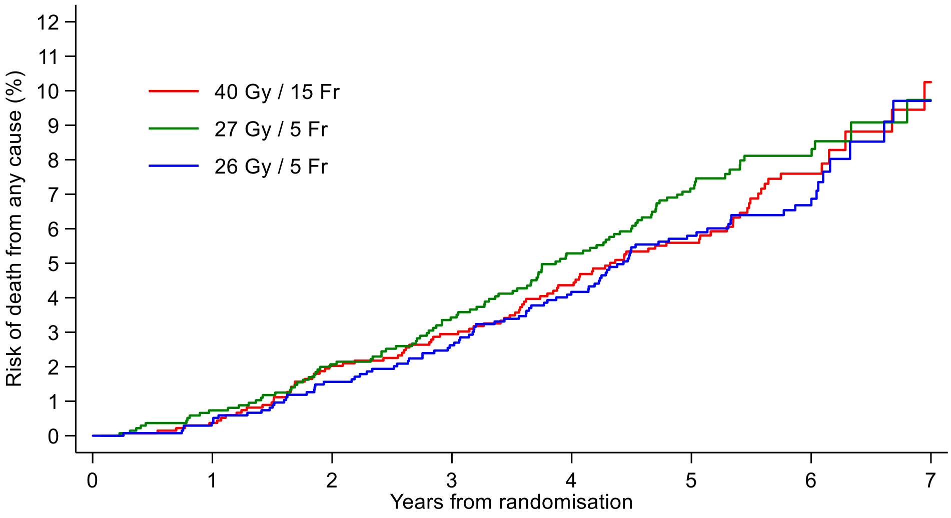

No. at risk (40Gy)	1361	1348	1312	1291	1245	1061	494	91
censored	0	8	22	31	59	228	780	1178
event	0	5	27	39	57	72	87	92
No. at risk (27Gy)	1367	1356	1337	1314	1271	1075	511	91
censored	0	1	2	7	26	199	755	1171
event	0	10	28	46	70	93	101	105
No. at risk (26Gy)	1368	1351	1332	1310	1266	1077	531	90
censored	0	12	15	23	47	216	755	1188
event	0	5	21	35	55	75	82	90

Figure A3: Clinician and patient assessments of late normal tissue effects up to 5 years by fractionation schedule

Clinician assessments | **Patient assessments**

(a) Breast distortion

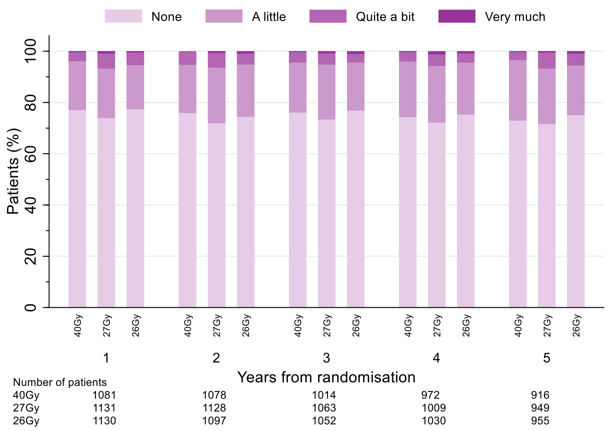

(b) Change in breast appearance

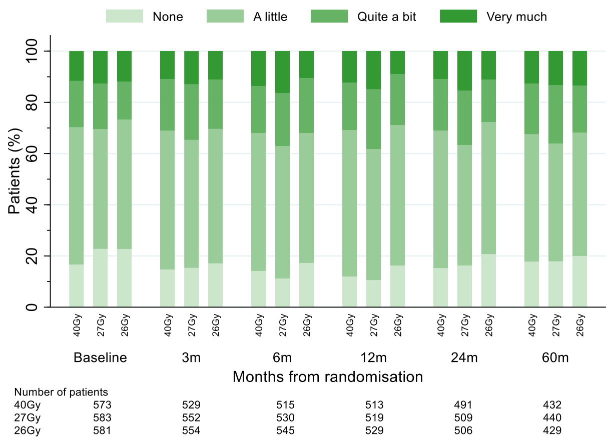

(c) Breast shrinkage

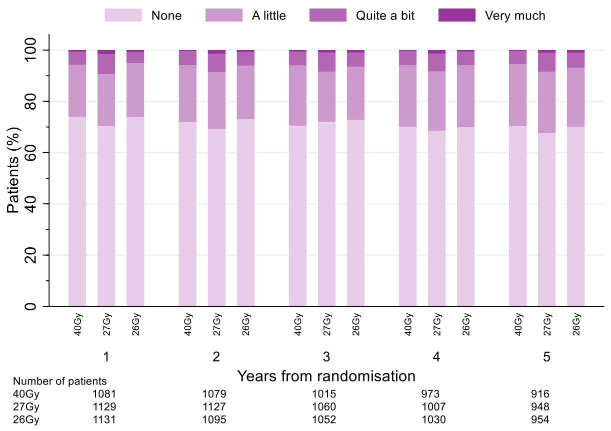

(d) Breast smaller

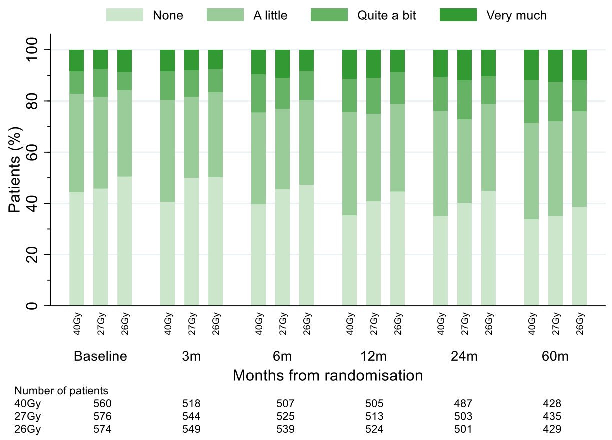

(e) Breast induration

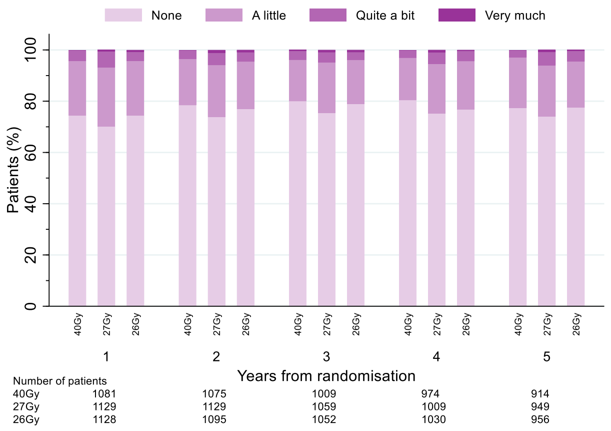

(f) Breast harder / firmer

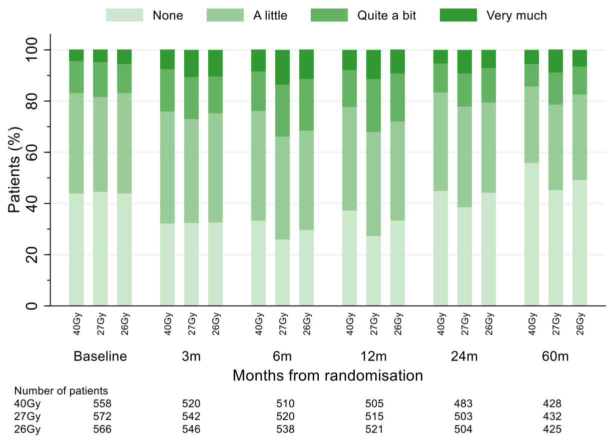

(g) Telangiectasia

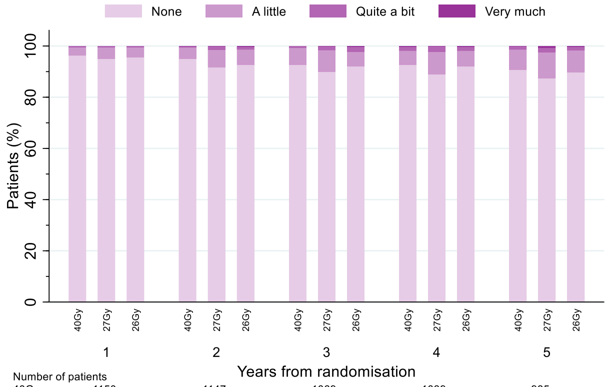

Number of patients	1	2	3	4	5
40Gy	1150	1147	1069	1039	985
27Gy	1189	1190	1117	1069	1004
26Gy	1196	1158	1113	1087	1018

(h) Change in skin appearance

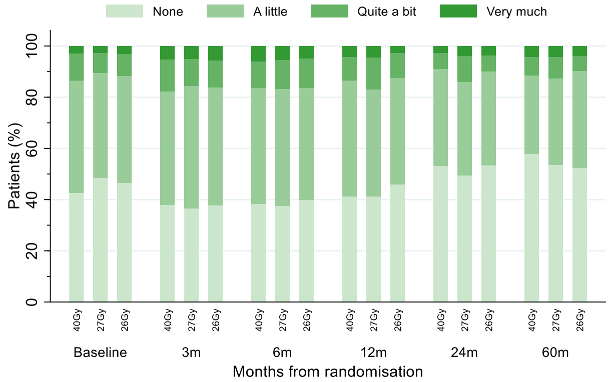

Number of patients	Baseline	3m	6m	12m	24m	60m
40Gy	577	535	523	518	495	434
27Gy	586	556	533	526	512	444
26Gy	580	558	546	534	507	431

(i) Breast oedema

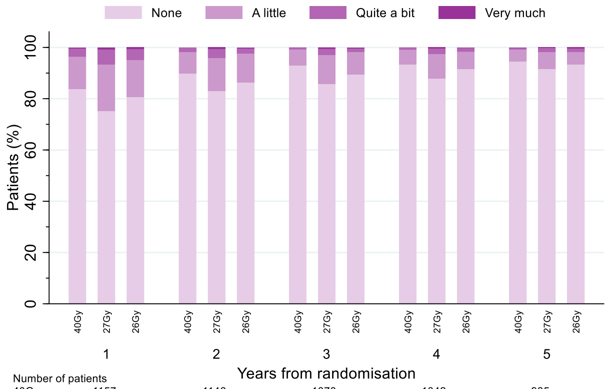

Number of patients	1	2	3	4	5
40Gy	1157	1146	1070	1042	985
27Gy	1196	1197	1122	1068	1002
26Gy	1202	1163	1118	1088	1018

(j) Breast swollen

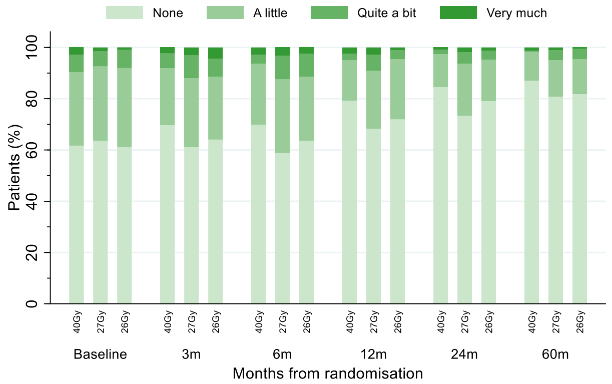

Number of patients	Baseline	3m	6m	12m	24m	60m
40Gy	583	538	527	527	506	440
27Gy	589	562	542	530	516	447
26Gy	589	560	552	539	513	435

(k) Breast discomfort

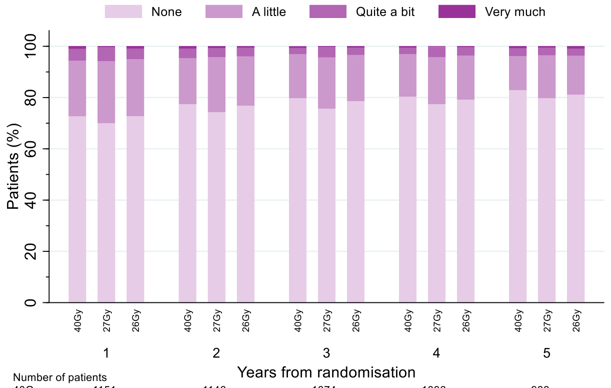

Number of patients	1	2	3	4	5
40Gy	1151	1146	1074	1036	983
27Gy	1186	1195	1127	1070	1004
26Gy	1194	1164	1120	1089	1015

(l) Breast pain

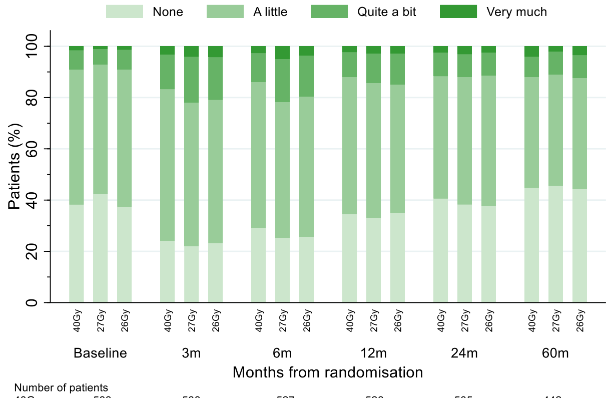

Number of patients	Baseline	3m	6m	12m	24m	60m
40Gy	583	538	527	526	505	442
27Gy	590	562	542	531	517	449
26Gy	588	560	552	538	511	436

Table A1: Locoregional and distant relapses by patient subgroups at randomisation and fractionation schedule

Subgroup	Ipsilateral breast tumour relapse			Regional relapse			Distant relapse		
	40 Gy / 15 Fr N=31	27 Gy / 5 Fr N=27	26 Gy / 5 Fr N=21	40 Gy / 15 Fr N=13	27 Gy / 5 Fr N=11	26 Gy / 5 Fr N=10	40 Gy / 15 Fr N=59	27 Gy / 5 Fr N=69	26 Gy / 5 Fr N=76
**Age at randomisation**									
**<50**	3	7	4	6	4	2	8	20	18
**≥50**	28	20	17	7	7	8	51	49	58
**Grade**									
**1**	2	3	4	1	0	0	2	2	2
**2**	9	9	9	7	3	4	20	24	30
**3**	20	15	8	5	8	6	37	43	44
**ER / HER2 status**									
**ER+ / HER2+**	3	4	1	0	2	1	6	7	9
**ER+ / HER2-**	17	15	16	9	3	4	36	44	42
**ER- / HER2+**	1	3	1	0	3	0	0	5	5
**ER- / HER2-**	10	5	3	3	3	5	16	13	18
**Unknown**	0	0	0	1	0	0	1	0	2

Table A2: Cross-sectional analysis of clinician-assessed late normal tissue effects at 5 years according to fractionation schedule for 3024 patients with 5-year assessments

	40 Gy N=990 (%)	27 Gy N=1008 (%)	26 Gy N=1026 (%)	Moderate/Marked vs. None/Mild								
				27Gy vs. 40Gy			26Gy vs. 40Gy			27Gy vs. 26Gy		
				Risk ratio (95%CI)	Risk difference (95%CI), %	p-value[1]	Risk ratio (95%CI)	Risk difference (95%CI), %	p-value[1]	Risk ratio (95%CI)	Risk difference (95%CI), %	p-value[1]
**Any AE in breast / chest wall[2*]**				1.55 (1.22,1.97)	5.5 (2.6, 8.4)	0.0003	1.19 (0.93,1.53)	1.9 (-0.8, 4.6)	0.17	1.30 (1.04,1.62)	3.6 (0.6, 6.5)	0.020
None	504 (51)	464 (46)	535 (52)									
Mild	384 (39)	386 (38)	364 (36)									
Moderate	93 (9)	132 (13)	105 (10)									
Marked	5 (<1)	23 (2)	16 (2)									
**Breast distortion+**				1.90 (1.25,2..88)	3.1 (1.2, 5.1)	0.0022	1.59 (1.03,2.44)	2.1 (0.2, 3.9)	0.035	1.20 (0.84,1.70)	1.0 (-1.0, 3.2)	0.34
None	669 (73)	680 (72)	717 (75)									
Mild	215 (23)	206 (22)	185 (19)									
Moderate	30 (3)	58 (6)	45 (5)									
Marked	2 (<1)	5 (<1)	8 (<1)									
**Breast shrinkage+**				1.51 (1.07,2.12)	2.8 (0.5, 5.0)	0.022	1.25 (0.87,1.78)	1.3 (-0.8, 3.5)	0.25	1.21 (0.88,1.66)	1.4 (-0.9, 3.8)	0.26
None												
Mild	645 (70)	642 (68)	670 (70)									
Moderate	221 (24)	228 (24)	219 (23)									
Marked	48 (5)	69 (7)	56 (6)									
	2 (<1)	9 (1)	9 (1)									
**Breast induration (tumour bed)+**				2.08 (1.31,3.31)	2.9 (1.1, 4.8)	0.0018	1.49 (0.91,2.44)	1.3 (-0.3, 3.0)	0.13	1.39 (0.93,2.08)	1.6 (-0.3, 3.5)	0.11
None	717 (78)	712 (75)	754 (79)									
Mild	172 (19)	183 (19)	163 (17)									
Moderate	24 (3)	49 (5)	35 (4)									
Marked	1 (<1)	5 (<1)	4 (<1)									
**Breast induration (outside tumour bed)+**				19.2 (2.58,142.9)	2.0 (1.1, 2.9)	<0.0001	19.1 (2.57, 141.9)	2.0 (1.0, 2.9)	<0.0001	1.01 (0.55,1.86)	0.0 (-1.3, 1.3)	>0.99
None	859 (94)	878 (93)	879 (92)									
Mild	51 (6)	50 (5)	56 (6)									
Moderate	1 (<1)	14 (1)	17 (2)									
Marked	0	6 (<1)	3 (<1)									
**Telangiectasia***				1.68 (0.87,3.23)	1.0 (-0.2, 2.2)	0.14	1.17 (0.58,2.37)	0.2 (-0.8, 1.3)	0.72	1.43 (0.77,2.65)	0.7 (-0.5, 1.9)	0.27
None	893 (91)	878 (87)	913 (90)									
Mild	78 (8)	102 (10)	88 (9)									
Moderate	13 (1)	18 (2)	15 (1)									
Marked	1 (<1)	6 (<1)	2 (<1)									
**Breast / chest wall oedema***				2.53 (1.06,6.02)	1.1 (0.1, 2.1)	0.042	2.35 (0.98,5.64)	1.0 (0.0, 1.9)	0.063	1.08 (0.56,2.07)	0.1 (-1.0, 1.3)	0.87

	40 Gy N=990 (%)	27 Gy N=1008 (%)	26 Gy N=1026 (%)	Moderate/Marked vs. None/Mild								
				27Gy vs. 40Gy			26Gy vs. 40Gy			27Gy vs. 26Gy		
				Risk ratio (95%CI)	Risk difference (95%CI), %	p-value[1]	Risk ratio (95%CI)	Risk difference (95%CI), %	p-value[1]	Risk ratio (95%CI)	Risk difference (95%CI), %	p-value[1]
None	931 (94)	918 (92)	950 (93)									
Mild	47 (5)	66 (6)	51 (5)									
Moderate	6 (<1)	16 (2)	13 (1)									
Marked	1 (<1)	2 (<1)	4 (<1)									
**Breast / chest wall discomfort***				0.95 (0.60,1.50)	-0.2 (-1.8, 1.5)	0.90	0.99 (0.63,1.56)	0.0 (-1.7, 1.6)	>0.99	0.96 (0.61,1.50)	-0.2 (-1.8, 1.5)	0.90
None	816 (83)	802 (80)	825 (81)									
Mild	131 (13)	167 (17)	153 (15)									
Moderate	29 (3)	31 (3)	29 (3)									
Marked	7 (<1)	4 (<1)	8 (<1)									
**Other RT-related***				1.12 (0.41,3.07)	0.0 (-0.7, 0.8)	>0.99	1.38 (0.53,3.60)	0.3 (-0.5, 1.1)	0.63	0.81 (0.32,2.05)	-0.2 (-1.0, 0.6)	0.81
None	967 (98)	984 (98)	996 (97)									
Mild	12 (1)	17 (2)	17 (2)									
Moderate	6 (<1)	4 (<1)	7 (<1)									
Marked	1 (<1)	4 (<1)	3 (<1)									

[1] p-value for Fisher's exact test; [2] Any AE in breast /chest wall includes breast distortion, breast shrinkage, breast induration (in & outside tumour bed), telangiectasia, breast / chest wall oedema

* BCS and mastectomy patients; + BCS patients and mastectomy patients with reconstruction

Table A3: Survival analyses of moderate/marked clinician-assessed late normal tissue effects by fractionation schedule for 3975 patients with at least one annual clinical assessment

Normal tissue effect	Moderate/ Marked events / total[2] (%)	KM estimate (95%CI) of cumulative incidence (%) of moderate/marked events by 5 years[3]	Hazard ratio (95%CI)	Comparison with 40Gy; p-value[4]	Comparison between 27Gy & 26Gy; p-value[4]
**Any NTE in the breast / chest wall[1]**					
40Gy	344/1307 (26.3)	26.8 (24.4, 29.4)	1		
27Gy	468/1339 (34.9)	35.1 (32.4, 37.9)	1.41 (1.23, 1.62)	<0.0001	
26Gy	380/1326 (28.7)	28.5 (26.0, 31.1)	1.09 (0.95, 1.27)	0.22	0.0002
**Breast distortion[5]**					
40Gy	126/1225 (10.3)	10.8 (9.1, 12.7)	1		
27Gy	190/1265 (15.0)	15.2 (13.2, 17.4)	1.50 (1.20, 1.88)	0.0004	
26Gy	159/1249 (12.7)	12.8 (11.0, 14.9)	1.25 (0.99, 1.57)	0.066	0.083
**Breast shrinkage[5]**					
40Gy	185/1227 (15.1)	14.9 (12.9, 17.1)	1		
27Gy	247/1265 (19.5)	19.1 (16.9, 21.5)	1.34 (1.11, 1.62)	0.0026	
26Gy	189/1249 (15.1)	146 (12.7, 16.9)	0.99 (0.81, 1.21)	0.95	0.0018
**Breast induration (tumour bed)[5]**					
40Gy	125/1225 (10.2)	10.3 (8.6, 12.2)	1		
27Gy	178/1266 (14.1)	14.0 (12.1, 16.2)	1.42 (1.13, 1.78)	0.0027	
26Gy	133/1249 (10.6)	9.9 (8.3, 11.8)	1.04 (0.81, 1.32)	0.78	0.0062
**Breast induration (outside tumour bed)[5]**					
40Gy	36/1225 (2.9)	2.9 (2.1, 4.1)	1		
27Gy	87/1266 (6.9)	6.7 (5.4, 8.3)	2.40 (1.63, 3.54)	<0.0001	
26Gy	52/1249 (4.2)	4.3 (3.2, 5.7)	1.42 (0.93, 2.17)	0.11	0.0024
**Telangiectasia**					
40Gy	36/1305 (2.8)	3.0 (2.1, 4.2)	1		
27Gy	59/1337 (4.4)	4.8 (3.7, 6.2)	1.61 (1.06, 2.44)	0.023	
26Gy	52/1324 (3.9)	3.5 (2.6, 4.8)	1.41 (0.92, 2.16)	0.11	0.49
**Breast / chest wall oedema**					
40Gy	72/1306 (5.5)	5.5 (4.3, 6.9)	1		
27Gy	140/1339 (10.5)	10.5 (8.9, 12.3)	1.95 (1.47, 2.59)	<0.0001	
26Gy	99/1326 (7.5)	7.5 (6.2, 9.2)	1.36 (1.01, 1.85)	0.045	0.0060
**Breast / chest wall discomfort**					
40Gy	156/1306 (11.9)	12.2 (10.5, 14.3)	1		
27Gy	178/1337 (13.3)	13.4 (11.6, 15.5)	1.12 (0.90, 1.39)	0.308	
26Gy	159/1327 (12.0)	11.8 (10.1, 13.8)	0.99 (0.79, 1.24)	0.94	0.27

KM = Kaplan-Meier, 95%CI = 95% confidence interval;

1 Any AE in the breast /chest wall = breast distortion, breast shrinkage, breast induration (in & outside tumour bed), telangiectasia and breast / chest wall oedema

2 Follow-up AE data available for 3978 patients (40Gy: 1309, 27Gy: 1340, 26Gy: 1329), denominators may vary due to missing clinician assessments for some events

3 Rate estimated at 5 years and 3 months to allow for visits occurring up to 3 months after the due date

4 p-value for pairwise logrank test

5 Not applicable after mastectomy (unless reconstructive surgery done)

Table A4: Cross-sectional analysis of patient-assessed late normal tissue effects at 5 years according to fractionation schedule for 1338 patients with 5-year questionnaire data available

	40 Gy N=444 (%)	27 Gy N=450 (%)	26 Gy N=444 (%)	Moderate/Marked vs. None/Mild								
				27Gy vs. 40Gy			26Gy vs. 40Gy			27Gy vs. 26Gy		
				Risk ratio (95%CI)	Risk difference (95%CI), %	p-value[1]	Risk ratio (95%CI)	Risk difference (95%CI), %	p-value[1]	Risk ratio (95%CI)	Risk difference (95%CI), %	p-value[1]
**Protocol-specific items**												
**Breast appearance changed**				1.11 (0.92, 1.33)	3.5 (-2.8, 9.8)	0.28	0.98 (0.80, 1.19)	-0.7 (-6.9, 5.5)	0.83	1.13 (0.94, 1.36)	4.2 (-2.1, 10.5)	0.20
None												
Mild	77 (17.8)	79 (18.0)	86 (20.1)									
Moderate	215 (49.8)	203 (46.1)	207 (48.2)									
Marked	86 (19.9)	100 (22.7)	79 (18.4)									
	54 (12.5)	58 (13.2)	57 (13.3)									
**Breast smaller**				0.98 (0.79, 1.21)	-0.7 (-6.7, 5.3)	0.88	0.84 (0.67, 1.05)	-4.5 (-10.4, 1.4)	0.14	1.16 (0.92, 1.45)	3.9 (-2.0, 9.6)	0.21
None	145 (13.9)	153 (35.2)	166 (38.7)									
Mild	161 (37.6)	161 (37.0)	160 (37.3)									
Moderate	72 (16.8)	67 (15.4)	52 (12.1)									
Marked	50 (11.7)	54 (12.4)	51 (11.9)									
**Breast harder/firmer**				1.49 (1.11, 2.01)	7.0 (2.0, 12.1)	0.0075	1.22 (0.89, 1.67)	3.2 (-1.7, 8.0)	0.22	1.22 (0.93, 1.61)	3.9 (-1.4, 9.2)	0.17
None	239 (55.8)	196 (45.4)	209 (49.2)									
Mild	128 (29.9)	144 (33.3)	142 (33.4)									
Moderate	38 (8.9)	54 (12.5)	47 (11.1)									
Marked	23 (5.4)	38 (8.8)	27 (6.3)									
**Skin appearance changed**				1.09 (0.77, 1.57)	1.1 (-3.2, 5.4)	0.68	0.85 (0.57, 1.25)	-1.8 (-5.9, 2.3)	0.44	1.29 (0.89, 1.89)	2.9 (-1.3, 7.0)	0.20
None	251 (57.8)	238 (53.6)	226 (52.4)									
Mild	133 (30.6)	150 (33.8)	163 (37.8)									
Moderate	32 (7.4)	37 (8.3)	26 (6.0)									
Marked	18 (4.1)	19 (4.3)	16 (3.7)									
**EORTC QLQ-BR23**												
**Breast pain**				0.91 (0.63, 1.31)	-1.1 (-5.3, 3.1)	0.67	1.03 (0.72, 1.47)	0.4 (-3.9, 4.7)	0.92	0.88 (0.61, 1.27)	-1.5 (-5.7, 2.8)	0.53
None	198 (44.8)	205 (45.7)	193 (44.3)									
Mild	191 (43.2)	195 (43.4)	189 (43.4)									
Moderate	35 (7.9)	40 (8.9)	39 (8.9)									
Marked	18 (4.1)	9 (2.0)	15 (3.4)									
**Breast swollen**				3.09 (1.33, 7.17)	3.3 (1.0, 5.6)	0.0072	2.75 (1.17, 6.46)	2.8 (0.5, 5.0)	0.017	1.13 (0.62, 2.05)	0.5 (-2.2, 3.3)	0.75
None	383 (87.1)	362 (81.0)	356 (81.8)									
Mild	50 (11.4)	63 (14.1)	60 (13.8)									
Moderate	2 (0.4)	18 (4.0)	17 (3.9)									
Marked	5 (1.1)	4 (0.9)	2 (0.5)									

	40 Gy N=444 (%)	27 Gy N=450 (%)	26 Gy N=444 (%)	Moderate/Marked vs. None/Mild								
				27Gy vs. 40Gy			26Gy vs. 40Gy			27Gy vs. 26Gy		
				Risk ratio (95%CI)	Risk difference (95%CI), %	p-value[1]	Risk ratio (95%CI)	Risk difference (95%CI), %	p-value[1]	Risk ratio (95%CI)	Risk difference (95%CI), %	p-value[1]
**Breast oversensitive**				0.95 (0.62, 1.46)	-0.4 (-4.2, 3.3)	0.90	1.21 (0.81, 1.81)	1.9 (-2.1, 5.8)	0.37	0.79 (0.52, 1.18)	-2.3 (-6.2, 1.6)	0.26
None												
Mild	253 (58.0)	252 (56.4)	252 (57.9)									
Moderate	144 (33.0)	157 (35.1)	136 (31.3)									
Marked	23 (5.3)	24 (5.4)	31 (7.1)									
	16 (3.7)	14 (3.1)	16 (3.7)									
**Skin problems on breast**				1.35 (0.76, 2.40)	1.5 (-1.4, 4.4)	0.36	1.12 (0.61, 2.06)	0.5 (-2.2, 3.3)	0.75	1.20 (0.68, 2.10)	1.0 (-2.0, 3.9)	0.55
None	347 (78.7)	338 (75.5)	351 (80.9)									
Mild	75 (17.0)	84 (18.7)	62 (14.3)									
Moderate	9 (2.0)	19 (4.2)	18 (4.1)									
Marked	10 (2.3)	7 (1.6)	3 (0.7)									
**Arm/shoulder pain**				0.81 (0.58, 1.12)	-3.0 (-7.6, 1.6)	0.21	1.02 (0.75, 1.39)	0.4 (-4.5, 5.2)	0.93	0.79 (0.57, 1.09)	-3.4 (-8.0, 1.3)	0.18
None												
Mild	230 (52.3)	230 (51.2)	236 (54.1)									
Moderate	141 (32.0)	162 (36.1)	130 (29.8)									
Marked	51 (11.6)	43 (9.6)	50 (11.5)									
	18 (4.1)	14 (3.1)	20 (4.6)									
**Arm/hand swollen**				0.76 (0.41, 1.42)	-1.2 (-3.9, 1.5)	0.42	1.01 (0.57, 1.80)	0.0 (-2.8, 2.9)	>0.99	0.75 (0.41, 1.40)	-1.2 (-4.0, 1.5)	0.41
None	370 (84.1)	375 (84.1)	365 (83.9)									
Mild	48 (10.9)	54 (12.1)	48 (11.0)									
Moderate	18 (4.1)	11 (2.5)	16 (3.7)									
Marked	4 (0.9)	6 (1.3)	6 (1.4)									
**Difficulty raising arm**				0.93 (0.59, 1.46)	-0.6 (-4.1, 3.0)	0.80	0.84 (0.53, 1.34)	-1.3 (-4.8, 2.2)	0.52	1.10 (0.69, 1.77)	0.7 (-2.7, 4.1)	0.70
None	326 (73.9)	336 (75.2)	321 (73.6)									
Mild	79 (17.9)	77 (17.2)	85 (19.5)									
Moderate	23 (5.2)	24 (5.4)	21 (4.8)									
Marked	13 (3.0)	10 (2.2)	9 (2.1)									

Denominators may vary due to missing items on the questionnaire; percentages are calculated out of totals with data available for each item; [1] p-value for Fisher's exact test

Table A5: Change in photographic breast appearance at 2 and 5 years (breast conservation surgery patients) by fractionation schedule: results of longitudinal analysis for 1309 patients with photographic assessments at 2 and/or 5 years

	2 years				5 years				OR for mild / marked change vs 40Gy (95%CI)	Comparison with 40 Gy; p-value[1]	Comparison between 27 Gy & 26 Gy; p-value[1]
	N	None (%)	Mild (%)	Marked (%)	N	None (%)	Mild (%)	Marked (%)			
**40 Gy / 15Fr**	411	376 (91.5)	33 (8.0)	2 (0.5)	283	249 (88.0)	33 (11.7)	1 (0.3)	1	-	-
**27 Gy / 5Fr**	429	362 (84.4)	48 (11.2)	19 (4.4)	308	225 (73.1)	70 (22.7)	13 (4.2)	2.29 (1.60, 3.27)	<0.0001	-
**26 Gy / 5Fr**	427	381 (89.2)	33 (7.7)	13 (3.0)	284	247 (87.0)	28 (9.9)	9 (3.2)	1.26 (0.85, 1.86)	0.24	0.0006

[1] p-value from Wald test; OR = odds ratio (estimated from GEE model including 2 and 5-year data); 95%CI=95% confidence interval

Table A6: Specialist referral for radiotherapy-related late adverse effect during follow-up, by fractionation schedule

Specialist referral type*	40 Gy N=1361 (%)	27 Gy N=1367 (%)	26 Gy N=1368 (%)
Lymphoedema	90 (6.6)	122 (8.9)	106 (7.7)
Breast surgery / breast surgeon	13 (0.9)	13 (0.9)	17 (1.2)
Cardiology	5 (0.4)	10 (0.7)	7 (0.5)
Pulmonary/Respiratory	4 (0.3)	6 (0.4)	3 (0.2)
Dermatology	4 (0.3)	2 (0.1)	3 (0.2)
Pain	6 (0.4)	5 (0.4)	2 (0.1)
Other	3 (0.2)	6 (0.4)	4 (0.3)

* Where patients had more than one type of referral, each is listed separately

Table A7: Incidence of other late adverse effects, by fractionation schedule

	40 Gy N=1361 (%)	27 Gy N=1367 (%)	26 Gy N=1368 (%)
**Symptomatic rib fracture**			
Reported[1]	14 (1.0)	25 (1.8)	20 (1.5)
Confirmed[2]			
Total	6 (0.4)	13 (1.0)	12 (0.9)
Ipsilateral side	*5 (0.4)*	*11 (0.8)*	*8 (0.6)*
**Symptomatic lung fibrosis**			
Reported[3]	9 (0.7)	10 (0.7)	10 (0.7)
Confirmed[2]			
Total	6 (0.4)	9 (0.7)	7 (0.5)
Ipsilateral side	*4 (0.3)*	*8 (0.6)*	*5 (0.4)*
**Ischaemic heart disease**			
Reported[4]	13 (1.0)	17 (1.2)	24 (1.7)
Confirmed[2]			
Total	12 (0.9)	11 (0.8)	10 (0.7)
Left-sided	*6 (0.4)*	*8 (0.6)*	*3 (0.2)*

[1] Reported cases of symptomatic rib fracture include 8 not radiotherapy-related (5 trauma, 1 metastases, 1 osteopenia, 1 reason not given but stated to be not due to radiotherapy)
[2] After imaging and further investigations; excluding cases not radiotherapy-related
[3] Reported cases of symptomatic lung fibrosis include 2 not radiotherapy-related (1 secondary to infection and 1 GI COPD)
[4] Reported cases of ischaemic heart disease include 17 patients with pre-existing heart disease at randomisation

FAST-Forward

Randomised clinical trial testing a 1-week course of curative whole breast radiotherapy against a standard 3-week schedule in terms of local cancer control and late adverse effects in patients with early breast cancer

**Chief Clinical Coordinators:**	Dr Murray Brunt (University Hospital of North Staffordshire)<br>Dr Duncan Wheatley (Royal Cornwall Hospital)
**Chief Investigator:**	Professor John Yarnold (The Institute of Cancer Research/<br>The Royal Marsden NHS Foundation Trust)
**Sponsor:**	The Institute of Cancer Research
**Funders:**	National Institute for Health Research - Health Technology Assessment programme.
**Coordinating Trials Unit:**	ICR Clinical Trials and Statistics Unit (ICR-CTSU)<br>The Institute of Cancer Research, Sutton.

PROTOCOL

Version 2.3: 11th Nov 2013

**ICR-CTSU Protocol Number:**	**ICR-CTSU/2010/10026**
**Main REC Reference Number:**	**11/LO/0958**
**Funder Reference Number:**	**09/01/47**

ISRCTN19906132

The FAST-Forward trial has been scientifically approved and funded by National Institute for Health Research - Health Technology Assessment programme.
The FAST-Forward trial is part of the National Institute for Health Research Clinical Research Network Trial Portfolio

This protocol is a controlled document and should not be copied, distributed or reproduced without the written permission of the ICR-CTSU

ADMINISTRATION

1. Clinical Coordination

Chief Clinical Co-ordinators	Chief Investigator
Dr Murray Brunt<br>University Hospital of North Staffordshire<br>Stoke-on-Trent ST4 6QG<br>Tel: 01782 672565<br>Email: murray.brunt@uhns.nhs.uk<br><br>Dr Duncan Wheatley<br>Royal Cornwall Hospital<br>Truro TR1 3LJ<br>Tel: 01872 258303<br>Email: duncan.wheatley@rcht.cornwall.nhs.uk	Professor John Yarnold<br>The Institute of Cancer Research<br>Royal Marsden Hospital<br>Downs Road<br>Sutton, Surrey SM2 5PT<br>Tel: 020 8661 3388<br>Fax: 020 8643 8809<br>Email: john.yarnold@icr.ac.uk

2. Biological Co-ordination

Blood Sample Collection	Tissue Sample Collection
Mark Sydenham<br>The Institute of Cancer Research<br>Sutton, Surrey SM2 5NG<br>Tel: 020 8722 4104<br>Email: mark.sydenham@icr.ac.uk	Mark Sydenham<br>The Institute of Cancer Research<br>Sutton, Surrey SM2 5NG<br>Tel: 020 8722 4104<br>Email: mark.sydenham@icr.ac.uk

3. Trial Coordination

ICR Clinical Trials & Statistics Unit (ICR-CTSU),
Division of Clinical Studies, The Institute of Cancer Research,
Sir Richard Doll Building, Cotswold Road, Sutton, Surrey SM2 5NG

ICR-CTSU (a UKCRC registered and NCRI accredited clinical trials unit) is responsible for the day to day conduct of the trial

ICR-CTSU Scientific Lead : Judith Bliss
Tel: 020 8722 4297
Email: judith.bliss@icr.ac.uk

PROMS co-ordinator: Wing Nip
Tel: 020 8722 4039
Email: wing.nip@icr.ac.uk

FAST-Forward Trial Manager: Mark Sydenham
Tel: 020 8722 4104
Email: mark.sydenham@icr.ac.uk

FAST-Forward Statistician: Clare Griffin
Tel: 020 8722 4062
Email: clare.griffin@icr.ac.uk

Any questions relating to this protocol should be addressed in the first instance to the FAST-Forward Trial Manager within ICR-CTSU:

Email: fastforward-icrctsu@icr.ac.uk

General enquiries: 0208 722 4104
Fax: 0208 770 7876

Protocol Development Group

Rajiv Agrawal, Royal Shrewsbury Hospital; Abdulla Alhasso, Beatson Oncology Centre; Peter Barrett-Lee, Velindre Hospital; Jane Beety, Norfolk and Norwich Hospital; Judith Bliss, ICR-CTSU, Sutton; Peter Bliss, Torbay Hospital; David Bloomfield, Royal Sussex County Hospital; Jo Bowen, Cheltenham General Hospital; Murray Brunt, University Hospital of North Staffordshire; Mark Churn, New Cross Hospital, Wolverhampton; Laura Ciurlionis, NCRI QA Team, Mount Vernon Hospital; Suzy Cleator, St Mary's Hospital, Paddington; Charlotte Coles, Addenbrooke's Hospital; John Dewar, Ninewells Hospital; Ellen Donovan, RMH Sutton; Paul Dyson, Cumberland Infirmary; Marie Emson, ICR-CTSU, Sutton; Andrew Goodman, Torbay Hospital; Susan Griffin, Centre for Health Economics, York; Sam Guglani, Cheltenham General Hospital; Adrian Harnett, Norfolk and Norwich University Hospital; Jo Haviland, ICR-CTSU, Sutton; Penny Hopwood, ICR-CTSU, Sutton; Judith Mills, ICR-CTSU, Sutton; Sarah McKenna, Independent Cancer Patients Voice; Carolyn Morris, Independent Cancer Patients Voice; Helen Passant, Velindre Hospital, Cardiff; Christine Rawlings, Torbay District General Hospital; Anne Robinson, Southend General Hospital; Elinor Sawyer, Guy's and St Thomas' Hospital; Mark Sculpher, Centre for Health Economics, York; Judith Sinclair, Charing Cross Hospital; Navita Somaiah, Radiobiology Institute, University of Oxford; Emma Staples, Queens Hospital, Romford; Georges Sumo, ICR-CTSU, Sutton; Mark Sydenham, ICR-CTSU, Sutton; Isabel Syndikus, Clatterbridge Centre for Oncology; Jean Tremlett , Royal Sussex County Hospital; Yat Tsang, NCRI QA Team, Mount Vernon Hospital; Karen Venables, NCRI QA Team, Mount Vernon Hospital; Duncan Wheatley, Royal Cornwall Hospital, Treliske; John Yarnold, Royal Marsden Hospital, Sutton

The Trial Management Group (TMG) will be constituted from members of the Protocol Development Group and principal investigators from a subset of participating centres. A copy of the current membership of the TMG can be obtained from the FAST-Forward Trial Manager within ICR-CTSU.

Protocol Authorised by:

**Name and Role**	**Date**	**Signature**
Professor John Yarnold (Chief Investigator)	**02/05/2013**	

This protocol describes the FAST-Forward trial and provides information about procedures for entering patients. The protocol should not be used as a guide for the treatment of other patients. Every care has been taken in the preparation of this protocol, but corrections or amendments may be necessary. These will be circulated to investigators in the trial, but centres

entering patients for the first time are advised to contact ICR-CTSU to confirm they have the most recent version. Protocol amendments will be circulated to participating centres as they occur.

This study will adhere to the principles outlined in the NHS Research Governance Framework for Health and Social Care and the principles of good clinical practice. It will be conducted in compliance with the protocol, the Data Protection Act and other regulatory requirements as appropriate.

This protocol is a controlled document and should not be copied, distributed or reproduced without the written permission of the ICR-CTSU.

TABLE OF CONTENTS

1. TRIAL SUMMARY 23

2. BACKGROUND 25

3. AIM 27

4. TRIAL DESIGN 27

4.1 Trial Schema 28

5. ENDPOINTS 29

5.1 Primary Endpoint 29
5.2 Secondary Endpoints 29

6. PATIENT SELECTION AND ELIGIBILITY 29

6.1 Patient Selection 29
6.2 Number of Patients 29
6.3 Inclusion Criteria 30
6.4 Exclusion Criteria 30

7. RANDOMISATION 30

7.1 Randomisation Procedure 30
7.2 Treatment allocation 31

8. TRIAL EVALUATIONS 31

8.1 Tumour-related Endpoints 31
8.2 Treatment-related Endpoints 31
8.2.1. Early adverse effects (only in centres taking part in the sub-study) 31
8.2.2. Late adverse effects 33

9. FOLLOW-UP 34

9.1 Withdrawal of Patients from Study Treatment and follow up 35
9.2. Schedule of assessments 36

10. RADIOTHERAPY 37

10.1 Dose Prescriptions 37
10.1.1 Whole breast/chest wall 37
10.1.2 Tumour bed boost 37

11. RADIOTHERAPY TARGET VOLUMES, LOCALISATION AND OUTLINING 38

11.1 Target Volume Definition 38
11.2 Patient Position 39
11.3 Acquisition of Outlines 39

12. RADIOTHERAPY PLANNING 39

12.1 Dose Constraints for Organs at Risk (OAR) 40
12.2 Bolus 40
12.3 Beam Energy 41
12.4 Tumour bed radiotherapy 41

13. TREATMENT SCHEDULING AND GAPS 41

14. RADIOTHERAPY VERIFICATION 41

14.1 Treatment Set-up Verification – Breast and Chest Wall 41
14.2 Treatment Set-up Verification - Boost 42
14.3 In-vivo Dosimetry 43

15. RADIOTHERAPY QUALITY ASSURANCE 43

16. SERIOUS ADVERSE EVENT REPORTING 43

16.1 Definitions 43
16.2 Reporting Serious Adverse Events 44
16.3 Reporting Related and Unexpected SAEs 44

17. STATISTICAL CONSIDERATIONS 44

17.1 Choice of Principal Outcomes 44
17.2 Methods of Analysis 45
17.3 Sample Size 46
17.3.1. Main Trial 46
17.3.2. Acute toxicity study I 47
17.3.3. Acute toxicity study II 47
17.3.4. Photographic, patient reported outcome measures (PROMS) and health economics (HE) sub-studies 47
17.4 Interim analyses and Data Monitoring 48

18. ASSOCIATED STUDIES 49

18.1 Molecular Correlates of Normal Tissue Injury 49
18.2 Molecular Correlates of Fractionation Sensitivity and Local Tumour Relapse 49
18.3 Patient Reported Outcome Measures (PROMS) Study 50
18.4 Health Economics (HE) 51

19. TRIAL MANAGEMENT 52

19.1 Trial Management Group 52
19.2 Trial Steering Committee 52
19.3 Independent Data Monitoring Committee 52

20. RESEARCH GOVERNANCE 53

20.1 Sponsor Responsibilities 53

21. TRIAL ADMINISTRATION AND LOGISTICS 54

21.1 Protocol Compliance 54

21.2 Protocol Amendments 54
21.3 Investigator Training 54
21.4 Data Acquisition...55
21.5 Central Data Monitoring...55
21.6 On site Monitoring..56
21.7 End of Study 56
21.8 Archiving..56

22. PATIENT PROTECTION AND ETHICAL CONSIDERATIONS 57

22.1 Risk Assessment 57
22.2 Patient Confidentiality..57
22.3 Ethical Considerations 57
22.4 Data Sharing..58
22.5 Data Protection Act (DPA)...58
22.6 Liability/Indemnity/Insurance 59

23. FINANCIAL MATTERS..59

24. PUBLICATION POLICY 59

25. REFERENCES...60

APPENDIX 1: ACUTE SKIN REACTIONS SCORING SCALE...62

APPENDIX 2: SELECTION OF TEST DOSE LEVELS FOR FAST-FORWARD...................63

APPENDIX 3: QUALITY ASSURANCE PROGRAMME...66

APPENDIX 4: PATIENT REPORTED OUTCOME MEASURES (PROMS) STUDY............69

APPENDIX 5: HEALTH ECONOMICS (HE)...76

1. TRIAL SUMMARY

**Title**	Randomised clinical trial testing a 1-week course of curative whole breast radiotherapy against a standard 3-week schedule in terms of local cancer control and late adverse effects in patients with early breast cancer.
**Aim**	To identify a 5-fraction schedule of curative radiotherapy delivered in 1 week that is at least as effective and safe as the UK standard 15-fraction regimen after primary surgery for early breast cancer.
**Eligibility Criteria**	*Inclusion criteria (all the following must be met):*<br>• age ≥18 years<br>• female or male<br>• invasive carcinoma of the breast<br>• breast conservation surgery or mastectomy (reconstruction is allowed)<br>• axillary staging &/or dissection<br>• complete microscopic excision of primary tumour<br>• pT1-3 pN0-1 M0 disease<br>• written informed consent<br>• able to comply with long-term follow up<br>N.B. Concurrent trastuzumab and/or endocrine therapies are allowed<br>*Exclusion criteria (the patient is ineligible if any of the following are met):*<br>• age ≥65 years and pT1G1/G2 ER+HER2-pN0 M0 invasive disease<br>• ipsilateral microinvasive disease and/or non-gradeable tumours<br>• past history of malignancy except (i) basal cell skin cancer, (ii) CIN cervix uteri or (iii) non-breast malignancy allowed if treated with curative intent and at least 5 years disease free<br>• contralateral and/or previous ipsilateral breast cancer, including DCIS, irrespective of date of diagnosis<br>• concurrent cytotoxic chemotherapy (sequential neoadjuvant or adjuvant cytotoxic therapy allowed as long as there is ≥ 2 weeks between therapy and radiotherapy)<br>• radiotherapy to any regional lymph node areas (excepting lower axilla included in standard tangential fields to breast/chest wall)
**Study Design**	Prospective randomised controlled clinical trial.
**Trial Treatment**	Patients are randomised to 15 or 5 daily fractions (Fr) to the whole breast or post-mastectomy chest wall/reconstructed breast. A sequential tumour bed boost may be added after breast conservation surgery, but dose level (10 Gy or 16 Gy in 2.0 Gy Fr) must be declared before randomisation. Each patient will be allocated to one of the following groups:<br>**Control Group**: 40.05 Gy in 15 Fr of 2.67 Gy<br>**Test Group 1**: 27.0 Gy in 5 Fr of 5.4 Gy<br>**Test Group 2**: 26.0 Gy in 5 Fr of 5.2 Gy
**Endpoints**	**Primary endpoint:** ipsilateral local tumour control<br>**Secondary endpoints:** early and late adverse effects in normal tissues, patient reported outcome measures of late adverse effects and quality of life, health economics, relapse free survival, disease free survival, time to distant metastases and overall survival.

**Sample Size**	The sample size is 4,000 patients, with numbers balanced equally in each randomised group. This provides 80% power (1-sided $\alpha = 0.025$ to allow for 1-sided hypothesis and multiple testing) to exclude an increase of 1.6% in the 5-year local relapse rate between each test group and the control, assuming a 5-year rate of 2% in the 40.05 Gy schedule. Stratification will be by centre and risk group (high- < 50 years or grade 3 vs. low - ≥ 50 years and grade 1 or 2).<br><br>For the photographic and patient reported outcome studies, 2196 patients will provide 80% power to detect an 8% difference in the prevalence of late adverse effects at 5 years between the test groups (assuming a 5-year rate of 35%). The health economics sub-study will involve the same 2196 patients in the photographic and patient reported outcome studies.<br><br>Acute toxicity was monitored in the first 190 patients in the trial, to exclude a rate of RTOG grade ≥3 acute skin reactions (using a modified RTOG scoring criteria) of over 11% (89% power and 7.9% significance). A second confirmatory acute toxicity study will monitor the acute skin reactions in a further 150 patients using the CTCAE scoring criteria.
**Follow Up**	Assessment of late toxicities and recurrence by clinical assessment will be incorporated into the annual follow up visits for all patients, with data collected for 10 years from the date of randomisation.
**Sub-Studies**	
**Acute Toxicity Study (in selected centres)**	One hundred and ninety patients were entered into a sub-study assessing acute toxicity in which a healthcare professional assessed acute skin reactions using a modified RTOG grading system that generated a combined score for moist desquamation and oedema.<br><br>A second acute toxicity study will be conducted in a further 150 patients using the CTCAE scoring criteria.
**Photographic AND**<br><br>**Patient Reported Outcome Measures (PROMS)**<br><br>**Sub-studies**<br><br>**(in the same subset of centres)**	Centres will be expected to take part in both the photographic and PROMS sub-studies and to offer both to all their patients until the sub-studies target accrual is reached.<br><br>Photographs will be taken at baseline and at 2, 5 and 10 years post randomisation in centres with photographic facilities.<br><br>Patient Report Outcome Measures (PROMS) will be assessed using the EORTC QLQ-C30 v3.0, the EORTC BR23 breast cancer module, the Body Image Scale (BIS), post-radiotherapy questions and the EORTC FA-13 fatigue module. PROMS questionnaires will be completed at baseline, 3 and 6 months post treatment and 1, 2, 5 and 10 years post randomisation.<br><br>NB: Patients can consent to both, either or neither of these sub-studies and still take part in the trial
**Health Economics (all PROMS patients)**	Health Economics (HE) will be assessed using the EQ-5D-5L questionnaire and additional health resource use questions completed at baseline, 3 and 6 months post treatment and 1, 2, 5 and 10 years post randomisation. The HE questions will be administered in the PROMS booklets
**Translational Studies**<br><br>**(in all centres)**	All patients will be asked to consent to donate a single blood sample and complete a family history questionnaire. This can be collected at any point during the trial.<br><br>All patients will be asked to consent to the donation of a tissue sample from their original tumour. They will also be asked to consent to the donation of a tissue sample should a recurrence occur.

2. BACKGROUND

The international standard regimen for whole breast radiotherapy delivers a total dose of 50 Gy in 25 fractions (daily doses) over 5 weeks following surgical resection of primary tumour in women with early breast cancer. Attempts to reduce the number of fractions in the 1970s made inadequate downward adjustments to total dose, resulting in unacceptable rates of late complications [1]. These miscalculations inhibited further research in breast radiotherapy fractionation for decades, but interest in fewer larger fractions delivered over a shorter overall treatment time has been rekindled by randomised clinical trials based on a better understanding of normal tissue and tumour responses. Four randomised trials involving a total of >8000 women have compared a lower total dose in fewer larger fractions against 50 Gy in 25 fractions, and all have reported favourable results in terms of local tumour control and late adverse effects [2-6].

The Royal Marsden Hospital/Gloucestershire Oncology Centre and Ontario trials totalling 2644 women with mainly axillary node negative tumours < 5 cm diameter were the subject of a 2008 Cochrane review of altered radiotherapy fractionation in early breast cancer [7]. Radiotherapy fractions larger than 2.0 Gy did not appear to affect: a) local-recurrence free survival (absolute difference 0.4%, 95% CI -1.5% to 2.4%), b) breast appearance (risk ratio (RR) 1.01, 95% CI 0.88 to 1.17; p = 0.86), c) survival at five years (RR 0.97, 95% CI 0.78 to 1.19; p = 0.75), d) late skin toxicity at five years (RR 0.99, 95% CI 0.44 to 2.22; p = 0.98, or e) late radiation toxicity in subcutaneous tissue (RR 1.0, 95% CI 0.78 to 1.28; p = 0.99). The review concluded that the use of unconventional fractionation regimens did not affect breast appearance or toxicity, nor appear to affect local cancer relapse. The results of the UK START trials (N = 4451) were published too late to be included in the overview, but were consistent with the findings. The UK START A trial (N=2236) showed that the estimated absolute differences in 5-year local-regional relapse rates compared with the control schedule of 50 Gy in 2.0 Gy fractions were 0.2% (95% CI -1.3% to 2.6%) after 41.6 Gy and 0.9% (95% CI -0.8% to 3.7%) after 39 Gy. In START A, photographic and patient self-assessments suggested lower rates of late adverse effects after 39 Gy than with 50 Gy, with a hazard ratio for late change in photographic breast appearance of 0.69 (95% CI 0.52 to 0.91, p=0.01). In the UK START B trial (N = 2215) the estimated absolute difference in 5-year local-regional relapse rates for 40.05 Gy compared with 50 Gy was -0.7% (95% CI -1.7% to 0.9%), and the hazard ratio for late change in photographic breast appearance was 0.83 (95% CI 0.66 to 1.04). i.e. the START trials reported similar local tumour control with some evidence of lower

rates of late adverse effects after schedules with fraction sizes larger than 2.0 Gy compared with the international standard 25-fraction regimen [6].

A 15-fraction schedule is now the UK standard recommended by the National Institute for Health and Clinical Excellence (NICE), but it is unlikely to represent the useful limits of hypofractionation for whole breast radiotherapy. There is a history of prescribing once-weekly fractions of whole breast radiotherapy for women too frail or otherwise unable to attend for conventional schedules. In a French series of 115 patients undergoing primary radiotherapy without surgery for non-metastatic breast cancer from 1987 to 1999, the whole breast was treated with 2 tangential fields and received 5 once-weekly fractions of 6.5 Gy [8]. 101 were given additional tumour bed boost doses, 7 with 1 fraction, 69 with 2 fractions and 25 with 3 once-weekly fractions of 6.5 Gy using electrons. Kaplan-Meier estimates of late effects in the breast were 24% grade 1, 21% grade 2 and 6% grade 3 at 48 months. The 5-year local progression-free rate was 78% (95% CI: 66.6-88.4). In a separate French series, 5 once-weekly fractions of 6.5 Gy to the whole breast with no boost were given to 50 women after local tumour excision [9]. Grade 1 or 2 induration was reported in 33% of the patients at a median follow up of 93 months (range 9-140). The 7-year local relapse free survival was 91%. Five fractions of 6.5 Gy are equivalent to 62 Gy in 31 fractions assuming α/β = 3.0 Gy, a significantly higher dose intensity than conventional schedules deliver.

The UK FAST Trial (N = 915) tested two dose levels of a 5-fraction regimen delivering 1 fraction per week against a control schedule of 50 Gy in 25 fractions, defining radiotherapy adverse effects as the primary endpoint [10]. The two test dose levels delivered 5 fractions of 5.7 Gy or 6.0 Gy (total dose 28.5 Gy or 30 Gy), estimated to be iso-effective with the control regimen assuming α/β values of 3.0 Gy or 4.0 Gy, respectively. 915 patients were recruited from October 2004 - March 2007. Mean age was 62.7 years. Only 17 patients (5.2%) developed moist desquamation (12 after 50 Gy, 3 after 30 Gy, 2 after 28.5 Gy) out of 327 with RTOG skin toxicity data available. At a median follow up of 28.3 months (IQR 24.1-33.6), 729 patients had 2-year photographic assessments available, with mild and marked change in breast appearance in 19.3% and 1.7% after 50 Gy, 26.2% and 9.3% after 30 Gy, and 20.3% and 3.7% after 28.5 Gy. Risk ratios for mild and marked change for 30 Gy vs. 50 Gy were 1.48 (95%CI 1.06 -2.05) and 6.06 (2.14 -17.20), $p<0.001$ for trend, favouring 50 Gy; and for 28.5 Gy vs. 50 Gy were 1.07 (0.75 -1.54) and 2.25 (0.70 -7.18), $p=0.26$ for trend, favouring 50 Gy. Any clinically-assessed moderate/marked adverse effects in the breast were increased for 30 Gy compared with 50 Gy (hazard ratio, HR 2.19, 95%CI 1.46 - 3.29, $p<0.001$), but similar

for 28.5 Gy (HR 1.33, 95%CI 0.86 -2.08, p=0.19). At a median follow-up of 37.3 months 2 local tumour relapses had been recorded.

A gain in local tumour control due to shortening treatment time to 1 week is possible. Evidence based on retrospective studies for an influence of treatment time on local tumour control is conflicting with recent systematic reviews drawing different conclusions [11, 12]. Even without a gain in tumour control, accelerated radiotherapy is likely to be more convenient for patients, and may ease scheduling with other treatment modalities. A pilot study (N = 30) tested 30 Gy in 5 fractions of 6.0 Gy in 15 days to the whole breast in terms of acute adverse effects and late effects at 2 years [13]. In this series, 23/30 (77%) patients scored no change in post-operative breast appearance at 2 years, 7/30 (23%) scored mild change and none scored marked change. The acute skin reactions were mild, with no reaction more severe than grade 2 erythema, scored in 9/30 (27%) patients. If the results of the proposed randomised trial support a 5-fraction schedule delivered in 1 week, these will transform international breast radiotherapy practices. In conclusion, it is fair to say that after decades of resistance to evaluating larger radiotherapy fraction sizes in breast cancer, expert opinion is responding to an accumulating body of evidence supporting the safety and effectiveness of this approach.

Against this background, a phase III randomised trial is described with the primary aim of testing local tumour control in women with early breast cancer following a 5-fraction schedule of adjuvant radiotherapy delivered in 1 week. Stratification by treatment centre and by local relapse risk will ensure balanced trial groups (high risk defined as patient age <50 and/or grade 3 tumour; low risk defined as age ≥50 and grade 1 or 2 tumour) [14]. From Version 2 of the protocol (13th Feb 2013) the population of patients with a very low risk of local relapse after breast conservation surgery comprising those aged at least 65 with pT1 G1/2 ER+ HER2- pN0 M0 invasive carcinomas are excluded from the trial following updated analyses by the Early Breast Cancer Collaborative Group [15].

3. AIM

To identify a 5-fraction schedule of curative radiotherapy delivered in once-daily fractions, that is at least as effective and safe as the current UK standard 15-fraction regimen after primary surgery for early breast cancer, in terms of local tumour control, adverse effects, patient reported outcome measures (PROMS) and health economic (HE) consequences.

4. TRIAL DESIGN

FAST-Forward is a multicentre phase III randomised controlled trial.

4.1 Trial Schema

FAST-Forward Trial Schema

Patient Population
Female or male, age ≥ 18; primary breast conservation surgery or mastectomy of invasive carcinoma (pT1-3 pN0-1 M0) with complete microscopic resection; whole breast/chest wall radiotherapy +/- tumour bed boost dose.

↓

Exclusion Criteria
- Patients aged 65 and over with pT1 G1/G2 ER+HER2- pN0 M0 invasive disease.
- Ipsilateral microinvasive disease and/or non-gradeable tumours
- Previous malignancy (other than basal cell skin cancer and CIN cervix uteri), or 5 years disease free following treatment with curative intent.
- Contralateral and/or previous ipsilateral breast cancer, including DCIS, irrespective of date of diagnosis.
- Concomitant chemotherapy (sequential chemotherapy allowed).
- Radiotherapy to lymphatic pathways.

↓

Patient eligible for FAST-Forward and consents to participate

↓

*Baseline Patients Reported Outcome Measures questionnaires – **prior to randomisation**
*Baseline health economics questionnaires – **prior to randomisation**
*Baseline photographs of breast/chest wall following surgery – **prior to radiotherapy**
Blood sample collection/family history questionnaire – **at any time**

↓

Randomise to one of three treatments

**Control Group**	**Test Group 1**	**Test Group 2**
40.05Gy / 15 Fr	27.0 Gy / 5 Fr	26.0 Gy / 5 Fr
3 weeks	1 week	1 week
2.67 Gy/Fr	5.4 Gy/Fr	5.2 Gy/Fr

16 Gy or 10 Gy in 2 Gy fractions sequential electron or photon boost to the tumour bed is allowed in all 3 treatment arms (boost decision to be declared before randomisation for each individual patient)

↓

Follow up
Acute toxicity study I – (completed) assessed the first 190 patients weekly during and for 4 weeks post radiotherapy, then weekly until RTOG ≤1 if symptoms persist
Acute toxicity study II – assess 150 patients, who are not receiving a boost dose, weekly during and for 4 weeks post radiotherapy. If moist desquamation persists then continue weekly assessments until CTCAE (moist desquamation) ≤1
Annual clinical assessment follow up for 10 years post radiotherapy
* Patient Reported Outcome Measures questionnaires at 3 & 6 months post radiotherapy and 1, 2, 5 and 10 years post randomisation
* Health economics questionnaires at 3 & 6 months post radiotherapy and 1, 2, 5 and 10 years post randomisation
* Photographs of breast/chest wall at 2, 5 and 10 years post radiotherapy
Tissue collection from primary and recurrence/new primary in either breast

*** - to start after the first acute toxicity study has completed**

5. ENDPOINTS

5.1 Primary Endpoint

- Ipsilateral local tumour control.

5.2 Secondary Endpoints

- Acute adverse effects
- Late adverse effects in normal tissues assessed by physicians and patients and from photographs
- Late adverse effects on quality of life assessed by patient reported outcome measures
- Health economics
- Contralateral primary tumours
- Relapse free survival
- Disease free survival
- Time to distant metastases
- Overall survival.

6. PATIENT SELECTION AND ELIGIBILITY

6.1 Patient Selection

Women and men with complete microscopic resection of early invasive breast cancer following breast conservation surgery or mastectomy for whom local radiotherapy is recommended (patients undergoing reconstruction are eligible provided the port of a tissue expander is positioned outside the breast).

6.2 Number of Patients

A total of 4000 patients will be recruited. The proportions of patients accrued in subgroups defined by risk of local recurrence will be monitored during the trial, to ensure reasonable representation of low risk (age $\geq$50 and grades 1 or 2) and high risk (age <50 and/or grade 3). From Version 2 of the protocol (13 Feb 2013) the population of patients with a very low risk of local relapse after breast conservation surgery comprising those age 65 and over with pT1 G1/2 ER+ HER2- pN0 M0 invasive carcinomas are excluded from the trial following updated analyses by the Early Breast Cancer Collaborative Group [15]

6.3 Inclusion Criteria

To be eligible, all of the following inclusion criteria must be met:

- age ≥18 years
- female or male
- invasive carcinoma of the breast
- breast conservation surgery or mastectomy (reconstruction is allowed)
- axillary staging &/or dissection
- complete microscopic excision of primary tumour
- pT1-3 pN0-1 M0 disease
- written informed consent
- able to comply with follow up

N.B. concurrent trastuzumab and/or endocrine therapies are allowed

6.4 Exclusion Criteria

The patient is ineligible if any one of the following exclusion criteria is met:

- age ≥65 years with pT1 G1/2 ER+ve / HER2-ve pN0 M0 invasive disease
- ipsilateral microinvasive disease and/or non-gradeable tumours
- past history of malignancy except (i) basal cell skin cancer, (ii) CIN cervix uteri or (iii) non-breast malignancy allowed if treated with curative intent and at least 5 years disease free
- contralateral and/or previous ipsilateral breast cancer, including DCIS, irrespective of date of diagnosis
- concurrent cytotoxic chemotherapy (sequential neoadjuvant or adjuvant cytotoxic therapy allowed as long as there is ≥ 2 weeks between therapy and radiotherapy)
- radiotherapy to any regional lymph node area (excepting lower axilla included in standard tangential fields to breast/chest wall)

7. RANDOMISATION

7.1 Randomisation Procedure

An eligibility checklist must be completed and patient consent obtained prior to randomisation.

To randomise a patient, the appropriate centre staff should telephone the ICR-CTSU randomisation line (see below).

Randomisation telephone: 020 8643 7150

Office Hours: 09:00 – 17:00 Monday-Friday

The following information will be required at randomisation:

- name of centre, consultant and person randomising the patient
- patient's full name, hospital number, date of birth, post code and NHS number
- confirmation that an eligibility checklist has been completed and written informed consent has been obtained
- whether the patient has consented to:
    - the acute toxicity study
    - photographic assessments
    - PROMS study
    - blood sample donation and family history questionnaire completion
    - tissue sample donation
    - the use of information held by the NHS and national databases.
- whether a boost is to be given and what dose level

The caller will be given the patient's unique randomisation number (Trial ID) and the treatment allocation. The Trial ID together with the patient's initials, date of birth and hospital number should be used on all Case Report Forms (CRFs).

7.2 Treatment allocation

Treatment allocation will be 1:1:1 and will use computer-generated random permuted blocks. Randomisation will be stratified by centre and risk group.

A fax will be sent to the randomising centre to confirm the trial number and treatment allocation.

8. TRIAL EVALUATIONS

8.1 Tumour-related Endpoints

Ipsilateral tumour relapse and contralateral primary tumour must be confirmed by cytological/histological assessment. Metastases will be determined by an appropriate combination of clinical, haematological, imaging and pathological assessment, recognising that pathological confirmation is not always possible. Patients will have annual clinical assessments for 10 years and annual mammograms for 5 years or until screening age if younger (as per NICE guidelines).

8.2 Treatment-related Endpoints

8.2.1. Early adverse effects (only in centres taking part in the sub-study)

Early adverse effects will be assessed in two acute toxicity studies.

Acute toxicity study I

190 patients were entered into a sub-study between November 2011 and April 2012 in order to assess the acute reactions of the skin of the treated breast. This sub- study used a modified RTOG scale (Appendix 1) in which effects for oedema and desquamation were reported in a combined outcome scale. The assessments were carried out weekly during treatment and for 4 weeks following the end of radiotherapy by a health care professional at each centre. Assessments were to continue weekly until any reaction was modified RTOG grade 1 or less.

In addition, patients were asked to report their own acute toxicity of breast radiotherapy (breast soreness, reddening, swelling and blistering) by completing a diary card weekly during treatment and for 4 weeks after the end of radiotherapy. The scores were recorded as "none", "a little", "quite a bit" or "very much". If symptoms persisted then patients were asked to continue scoring their adverse effects on a weekly basis until all scores were graded as "none" or "a little".

This sub-study was completed in summer 2012 with a review of the data by the Independent Data Monitoring Committee (IDMC) and Trial Steering Committee (TSC). The review highlighted that the data had been collected using a modified RTOG scoring criteria that had not allowed the prospective differentiation between moist desquamation and moderate oedema for those classified with a "grade 3" acute skin reaction and recognised the need to differentiate between these two reactions due to their differential potential to be dose-limiting. Furthermore, the adherence to the intensive weekly follow-up schedule was not as complete as expected with assessments missed both during and after treatment for some patients.

Acute toxicity study II (150 evaluable patients)

The IDMC/TSC requested that a second sub-study be conducted using the CTCAE v4.03 scoring criteria which separately records incidences of moist desquamation and oedema.

The acute toxicity sub-study II will be conducted in a subset of centres which have the infrastructure necessary to carry out the weekly toxicity assessments.

The acute reactions of the skin of the treated breast will be graded for erythema and moist desquamation using standard CTCAE criteria (Appendix 1), and will be assessed by a healthcare professional at each centre. The assessments will be carried out weekly during treatment and for 4 weeks following the end of radiotherapy. If moist

desquamation outside skin folds or creases is seen during this time then weekly assessments will continue until the reaction has resolved to CTCAE grade 1 or less. If any assessment is missed then the centre will be asked to contact the patient by telephone to ascertain the reason for the missed assessment and ask about any acute skin reactions. Patients receiving a boost will be excluded from acute toxicity sub-study II, since the objective of this sub-study is to quantify the toxicity of 5-fraction schedules relative to control, effects that are independent and additive to those of the boost.

8.2.2. Late adverse effects

The late adverse effects include a range of symptoms and signs, including breast swelling and/or oedema, breast shrinkage, hardness, telangiectasia, pigmentation, skin atrophy, subcutaneous fat necrosis, skin necrosis, pain and tenderness, cardiac injury and lung fibrosis. Late adverse effects will be measured in all patients at the annual clinical assessment and in a subset of patients using photographic assessments and patient-reported outcome measures (PROMS) questionnaires.

Clinical assessments of late adverse effects (for all patients)

At annual visits for 10 years (from date of randomisation into study) physicians will record the development of breast shrinkage/distortion (including reconstructed breasts), breast induration (outside and inside tumour boost volume), breast pain and breast oedema (for patients receiving radiotherapy following breast conserving surgery) and telangiectasia (tumour boost site only), shoulder stiffness (compared with other side), ischaemic heart disease, rib fracture, costochondritis, symptomatic lung fibrosis, persistent cough and any other severe late event, including any specialist referral for investigation or management of late toxicity.

Photographic and PROMS assessments of late adverse effects (in the same patients)

Photographic assessments

Digital photographs will be taken at baseline (post-surgery but pre-RT) and at years 2, 5 and 10 after randomisation. Timing of assessments is based on experience from the START trial, with the aim to maximise the information collected whilst minimising the assessment burden. Two frontal views of the chest will be taken, one with hands on the hips and the other with hands raised as far as possible above the head. Both photographs will exclude the patient’s head.

All photographs will be taken and retained locally in the first instance. Digital images will be coded and stored on a CD to be kept in a secure location. Periodically all CDs will be collected by ICR-CTSU and the images assessed blind by a select group of observers and/or using computer software adapted for the purpose. Change in breast/reconstructed breast/chest wall appearance and distortion compared with the post-surgical baseline will each be scored on a graded scale. Breast size and surgical deficit will each be assessed from the baseline photographs. Reliability and repeatability of the assessments will be verified. The feasibility of and procedures for this scoring mechanism have been established for breast conserving surgery patients in the START trial [16] and assessments for FAST-Forward will build on these existing methods, including validating the method in chest wall patients.

PROMS assessments

Patients will be asked to complete self-assessments of radiotherapy adverse effects and other PROMS at baseline, 3 and 6 months after radiotherapy and 1, 2, 5 and 10 years from randomisation. These will include the EORTC QLQ-C30 core questionnaire [17], the EORTC BR-23 Breast Cancer module [18], the Body Image Scale [19], the EORTC FA-13 questionnaire [20] and a number of protocol-specific items relating to radiotherapy adverse effects as used in the START and IMPORT trials[21]. Of particular interest will be patient self-reporting of symptoms and impact on body image and functioning subscales. The aim will be to seek a patient-derived notion of 'radiation tolerance' that can be compared with physician and photographic endpoints, including interpolated estimates of isoeffect.

9. FOLLOW-UP

After treatment clinical follow up should follow local guidelines.

For the purpose of the study, assessment of acute toxicities will be performed in the patients consenting to the acute toxicity sub-studies only (see section 8.2.1.). Assessments of late toxicities using photographs and PROMS will be performed in patients consenting to these sub-studies only (according to the schedule outlined in section 8.2.2.)

Assessment of late toxicities and recurrence by clinical assessment will be incorporated into the annual follow up visits for all patients, with data collected for 10 years from the date of randomisation.

9.1 Withdrawal of Patients from Study Treatment and follow up

Patients who do not receive their allocated treatment for any reason should be treated at the discretion of their clinician. Unless the patient requests otherwise, all CRFs, including long term follow up, should be completed, regardless of treatment actually received. A trial deviation form should be completed to record details of deviation from treatment allocation. Analyses of all outcome data will be on the basis of intention to treat. As this is a non-inferiority trial if there is high non-compliance with the test treatment groups then an analysis of only those compliant with the protocol will also be conducted.

Patients are asked prior to randomisation to consent to follow up should they withdraw from the treatment allocation (see patient information sheet and consent form), and any patient unwilling to give that assurance prior to trial entry should not be randomised. Patients are however free to reverse that decision at any time without giving a reason. If a patient withdraws consent for further follow-up and for PROMS data to be collected, the appropriate form in the CRF should be completed and returned to ICR-CTSU. In the extremely unlikely event that the patient wishes to have their data removed from the trial completely the implications of this should be discussed with the patient to ensure that this is their intent and this should be recorded on the withdrawal of consent CRF.

Should a patient become incapacitated at any point during the trial they will be withdrawn for their own protection. If this were to happen during the course of the patient's radiotherapy their treatment should be reviewed as a clinical decision by the Principal Investigator at their centre. No further trial procedures will be carried out and only data that is routinely collected i.e. disease status, vital status, cause of death will be used on behalf of the trial. Any samples already donated, i.e. blood and tissue, will be retained and used for the original research purpose. These procedures are fully explained in the patient information sheet, and patients are asked to consent to this prior to randomisation. A trial deviation form should be completed for any patient withdrawn from the trial for this reason.

9.2. Schedule of assessments

Event		Prior to randomisation	Post randomisation pre RT	Treatment: wk 1	Treatment: wk 2	Treatment: wk 3	Follow up (all taken from date of randomisation except where shown): weekly for 4 weeks post RT	mth 3 (Post RT)	mth 6 (Post RT)	yr 1	yr 2	yr 3	yr 4	yr 5	yr 10
Eligibility checklist		x													
Informed consent		x													
Randomisation checklist		x													
Radiotherapy QA		**Prior to centre initiation and throughtout the trial recruitment period**													
3D radiotherapy planning			x												
Radiotherapy treatment				x	x[1]	x[1]									
Radiotherapy verification				**Up to daily during treatment**											
Serious Adverse Event (if applicable)				x	x	x	x	x							
Acute toxicity assessments	Study I (first 190 patients)		x	x	x[1]	x[1]	x								
	Study II (150 patients, no boost)		x	x	x[1]	x[1]	x								
Follow up - annual clinical assessment (all patients)										x	x	x	x	x	x (Annually yrs 6-10)
**Sub studies**															
PROMS [2]		x (baseline*)						x	x	x	x			x	x
Photographic assessment [2]			x								x			x	x
Health economics - annual assessment		x (baseline*)						x	x	x	x	x	x	x	x (Annually yrs 6-10)
Blood sample collection and family history questionnaire			**At any time during the trial, ideally by the end of RT**												
CT scan if recurrence				**At the time of recurrence**											
Tissue collection<br>- 1° tumour<br>- recurrence/new 1° tumour			**As requested during the trial**												

*Follow up booklets will be sent by post from the ICR-CTSU office; [1] Control group only; CRFs to be completed throughout the trial as indicated in the Trial Guidance Notes; [2] PROMS and photographic assessments are to be offered to the same set of patients

10. RADIOTHERAPY

Patients are randomised to 15 or 5 daily fractions (Fr) to the whole breast or post-mastectomy chest wall. A sequential tumour bed boost may be added after breast conservation surgery, but dose level (10.0 Gy or 16.0 Gy in 2.0 Gy Fr) must be declared before randomisation. Each patient will be allocated to one of the following groups:

Control Group: 40.05 Gy in 15 Fr of 2.67 Gy
Test Group 1: 27.0 Gy in 5 Fr of 5.4 Gy
Test Group 2: 26.0 Gy in 5 Fr of 5.2 Gy

10.1 Dose Prescriptions

10.1.1 Whole breast/chest wall

Trial group	Total dose (Gy)	Dose per fraction (Gy)	Number of fractions	Fractions per week	Treatment time (weeks)
***Control Group**	40.05	2.67	15	5	3
**#Test Group 1**	27.0	5.4	5	5	1
**#Test Group 2**	26.0	5.2	5	5	1

Justification for choice of these regimens is found in Appendix 2

10.1.2 Tumour bed boost

If a tumour bed boost dose is recommended, this needs to be declared before randomisation for each patient, together with the dose to be used. The dose prescription is either 10.0 Gy in 5 fractions or 16.0 Gy in 8 fractions to the 100% isodose, treating once-daily, and the boost must follow whole breast radiotherapy without a break. A boost is suggested for all patients under 40 years and for patients aged 40-49 years with either grade 3 tumours and/or lymphovascular invasion. A boost is also suggested for patients aged 50-59 years with one or more adverse prognostic factor, such as grade 3 tumours or lymphovascular invasion. There are no suggested indications for a boost in patients aged ≥60 years. No bolus should be used for boosts.

11. RADIOTHERAPY TARGET VOLUMES, LOCALISATION AND OUTLINING

11.1 Target Volume Definition

Whole Breast Clinical Target Volume (WBCTV)

This is based on the recommendations in the START trial protocol [22]. The CTV includes the soft tissues of the whole breast from 5 mm below the skin surface down to the deep fascia, excluding muscle and underlying rib cage.

Chest Wall Clinical Target Volume (CWCTV)

The clinical target volume encompasses the skin flaps and includes the soft tissues down to the deep fascia, excluding the underlying muscle and rib cage.

Tumour bed

Delineation of the tumour bed is recommended for all patients who had breast conserving surgery as this facilitates appropriate placement of the tangential breast field to maximise target coverage whilst and minimising dose to organs at risk (OAR). Examples are shown in the planning pack.

To assist the delineation, it is strongly advised that titanium clips or gold seeds are implanted into the walls of the tumour excision cavity (tumour bed) at the time of breast conserving surgery as per British Association of Surgical Oncology (BASO) guidelines [23]. The tumour bed may be localised if there is a well-defined seroma in the absence of implanted markers. Either of these localisation methods will be necessary if the boost radiotherapy is to be delivered with a conformal photon plan.

Planning Target Volumes (PTV)

A margin should be added to whole breast and tumour cavity CTV, taking into account set-up error, breast swelling and breathing; a typical PTV margin is 10 mm for both whole breast and tumour bed. A field-based whole breast PTV can be used and this method is illustrated in the planning pack.

Organs at Risk (OAR)

It is mandatory to contour ipsilateral lung and heart for dose volume histogram assessment. The heart should be outlined from the inferior aspect above the diaphragm, to the superior aspect below the pulmonary arch. Volumes are recorded for the purposes of the trial.

11.2 Patient Position

The patient must lie supine in a stable and reproducible position. The same position must remain for simulation, CT scanning and treatment. An immobilisation device, such as a breast board with arm and wrist supports, an arm pole and/or vac-fix bag should be used. Ideally, the immobilisation should allow daily reproducibility of +/- 5 mm. The patient must not be moved between tangential fields.

11.3 Acquisition of Outlines

A full 3D set of outlines covering the whole breast and the organs at risk must be collected with a slice separation of no more than 5 mm. The imaging technology to be used must be x-ray CT only to provide accurate dose-volume histogram (DVH) data for plan assessment.

12. RADIOTHERAPY PLANNING

It is compulsory to outline target volumes and the relevant organs at risk for radiotherapy planning of FAST-Forward patients. All computer planning must be carried out on a 3D dataset, and correction for tissue heterogeneity must be applied. Usually, a tangential pair beam arrangement is used to encompass the whole breast PTV, minimising the ipsilateral lung and heart in the fields. The treatment plan must be optimised with 3D dose compensation aiming to fulfil the criteria in Table 2 below.

Upper and lower dose limits for whole breast PTV

Lower dose limit	Prescription dose	Upper dose limit
>95% of the volume should receive 95% of the prescribed dose	Use a clinical relevant normalisation point for tangents, seek QA advice for inverse-planned	<5% of the volume should receive ≥105%<br><2% of the volume should receive ≥107%<br>global max <110% of the prescribed dose

Table 2: upper and lower dose limits for whole breast PTV

12.1 Dose Constraints for Organs at Risk (OAR)

The dose constraints for whole breast radiotherapy using tangential field arrangements are listed below. If non-tangential fields are used, e.g. inverse planned IMRT for patients with pectus excavatum or very medial tumour bed, then the planner must seek advice of the QA team. These constraints do not take into account the tumour bed boost dose (please refer to the planning pack for conformal photon boost organ at risk dose constraints). Although maximum dose constraints are stated for the heart, the planner should aim to keep any dose to the heart as low as possible.

Control Group

- The volume of ipsilateral lung receiving 12.0 Gy should be less than 15%
- The volume of heart receiving 2.0 Gy and 10.0 Gy should be less than 30% and 5% respectively.

Test Group 1 and 2

- The volume of ipsilateral lung receiving 8.0 Gy should be less than 15%
- The volume of heart receiving 1.5 Gy and 7.0 Gy should be less than 30% and 5% respectively.

Dose per fraction (Gy)	Keep 30 % of dose to < 15 % of ipsilateral lung volume	Keep 25 % of dose to < 5 % of heart volume	Keep 5 % of dose to < 30 % of heart volume
2.67	12.0 Gy	10.0 Gy	2.0 Gy
5.2/5.4	8.0 Gy	7.0 Gy	1.5 Gy

12.2 Bolus

Centres should specify prior to randomisation whether or not post-mastectomy (+/- reconstruction) bolus is to be applied, and if so, whether it is to be applied a) to part (e.g. the scar area) or all of the chest wall, b) for all or a specified number of fractions and c) thickness of bolus used for a given photon energy. Either composite plans, or plans with and without bolus are to be sent to the QA team for DVH assessment. Bolus is not applied after breast conservation surgery.

12.3 Beam Energy

Beam energies for treatment as for local practice, usually 6 MV, but a mixture of energies e.g. 6 MV and 15 MV can be used for larger patients.

12.4 Tumour bed radiotherapy

The tumour bed boost treatments can be either delivered by electron or photon beams. Either 10.0 Gy in 5 fractions or 16.0 Gy in 8 fractions is prescribed to the 100% isodose. Centres should aim to contour the boost volume and, where possible, produce dose distributions on their planning system and send boost plans to the QA team. If clinical mark up is used for planning, CT information must be used to guide localisation of the tumour bed, for example, using the information on clip position and the use of surface rendered views (if these can be produced from the planning system). Details on minimum requirements for tumour bed boost radiotherapy can be found in the planning pack.

13. TREATMENT SCHEDULING AND GAPS

Treatment can start on any day of the week.

A gap of up to 3 days is acceptable in the event of machine service or breakdown. This is preferable to transferring the patient to a machine on which daily verification imaging is not available. If the treatment machine is unavailable for more than 3 days, please contact the QA team.

14. RADIOTHERAPY VERIFICATION

14.1 Treatment Set-up Verification – Breast and Chest Wall

Verification is carried out using electronic portal imaging of the treatment beam. This can be either MV or kV.

Control Group: Treatment verification is required for at least three fractions in the first week of treatment to determine and correct for any systematic error*. Correction is carried out following local practice as long as this has been approved by the QA team. This correction is applied on fraction 4, and a further image may be taken to confirm the move. A suitable tolerance for the check of the correction is 5 mm. Verification is then once weekly throughout the remaining treatment with a tolerance of 5 mm.

Test Group 1 and 2: Verification imaging is required for each fraction to check for a gross error. A tolerance of not more than 5 mm should be used. Local policy is followed if the check is out of tolerance. A further image may be taken to confirm the correction and this also applies where daily imaging is used to correct couch position before treatment. Best practice is to correct all measured displacements.

If MV tangential fields are used for verification imaging, the method to derive the couch correction follows local practice as long as this has been approved by the QA team.

14.2 Treatment Set-up Verification - Boost

Electron Boost

The electron boost set up is verified daily by visual matching to marks on the skin and checks on the gantry and collimator angles required for matching.

Photon Boost

If photon mini-tangent fields are used, the first 2 or 3 fractions are imaged (as appropriate for the fractionation scheme). A correction for the systematic error is made for the remaining fractions*. A check of the correction may be made on fraction 6 for the 16.0 Gy in 8 fraction schedule; a suitable tolerance is 5mm.

Alternatively, as the fractionation schedules are short, daily imaging maybe used as described for the whole breast/chest wall Test Groups 1 and 2.

If a conformal photon boost is used, then daily imaging and correction is recommended for both fractionation options (10.0 Gy in 5 fractions or 16.0 Gy in 8 fractions). Best practice is to correct all measured displacements.

*All systematic errors should be corrected and this is recommended, but if a centre wishes to use a correction tolerance on systematic error it should not be greater than 5 mm, and preferable not more than 3 mm and reported to the QA team

Where the need for more complex treatment planning (e.g. inverse planning or tomotherapy) requires a verification method not described here, centres are requested to discuss this on an individual basis with the QA Team. Similarly, if a centre wishes to use a tighter PTV margin with a more stringent verification protocol, this should be discussed with the QA Team.

14.3 In-vivo Dosimetry

In line with current UK guidelines, all FAST-Forward patients should have in-vivo dosimetry within the first week of treatment. This may be performed using diodes or thermo-luminescent dosimetry (TLD). Other methods may be appropriate for individual centres and should be discussed with the QA team.

15. RADIOTHERAPY QUALITY ASSURANCE

A comprehensive quality assurance programme is planned for all centres involved with FAST-Forward (see Appendix 3).

16. SERIOUS ADVERSE EVENT REPORTING

16.1 Definitions

Adverse Event (AE): any untoward medical occurrence in a patient or clinical trial subject administered a research procedure; events do not necessarily have a causal relationship with the procedure.

Related Adverse Event: an adverse event assessed by the Principal Investigator or Chief Investigator as reasonably likely to be related to the administration of a research procedure.

Serious Adverse Event (SAE): an untoward occurrence that:

1. results in death
2. is life-threatening
3. requires hospitalisation or prolongation of existing hospitalisation
4. results in persistent or significant disability or incapacity
5. consists of a congenital anomaly or birth defect
6. is otherwise considered medically significant by the Principal Investigator

Related Unexpected Serious Adverse Events: an adverse event that meets the definition of serious and is assessed by the CI or nominative representative as:

- "Related" – that is, it resulted from administration from the research procedure, and "Unexpected" – that is, the type of event is not listed as an expected occurrence

16.2 Reporting Serious Adverse Events

All SAEs should be reported within 24 hours of the investigator becoming aware of the event, by completing the FAST-Forward SAE form and faxing it to the FAST-Forward Trial Manager, Clinical Trials and Statistics Unit, 020 8722 4369 (Monday – Friday 09.00-17.00). The SAE form must be completed, signed and dated by the Principal Investigator or nominated person identified on the centre delegation log. ICR-CTSU will send a fax back to the centre to acknowledge receipt of the SAE.

The Chief Investigator (or a nominated representative) will review all SAEs to assess relatedness and expectedness.

Any relevant follow up information, including final resolution of the event, should be completed on the relevant part of the original SAE form and faxed to the ICR-CTSU, within 15 days of the local investigator becoming aware of this information.

The centre SAE log should be completed and the SAE form filed in the Site Investigator File.

SAEs will be collected during the patient’s radiotherapy and for three months following treatment.

16.3 Reporting Related and Unexpected SAEs

If an SAE is defined as related and unexpected by the Chief Investigator, ICR-CTSU will report the SAE to the main REC within 15 days from the date the ICR-CTSU became aware of the event. Any subsequent reporting will be carried out as appropriate.

N.B. Patients showing unexpectedly severe late normal tissue responses will be identified on the Follow-up Forms and are not reported as SAEs. These late-occurring reactions include unexpectedly severe late subcutaneous fibrosis, ischaemic heart disease (after both right- and left-sided radiotherapy), rib fracture and symptomatic lung fibrosis.

17. STATISTICAL CONSIDERATIONS

17.1 Choice of Principal Outcomes

Primary outcome is ipsilateral local tumour control, since this is the justification for treatment. Other endpoints include normal tissue effects, PROMS and health economic considerations. It is intended that each endpoint will be analysed separately. If there is

discordance between the endpoints in terms of treatment outcome this will allow discussion of clinical trade-offs.

17.2 Methods of Analysis

Survival analysis methods (i.e. Kaplan-Meier analysis and Cox proportional hazards regression) will be used to compare rates of local recurrence between allocated treatments for all randomised patients (i.e. intention to treat). Normal tissue effects will be analysed using methodology developed for the START Trials; i.e. survival analyses of time to occurrence of moderate or marked effects, as appropriate. Analysis of the PROMS data will follow algorithms developed for the PROMS forms (i.e. calculation of standardised sub-scale scores), and will compare treatment groups at individual time points, as well as longitudinal changes from baseline. A generalised linear modelling approach will be used to describe the longitudinal PROMS data, taking into account important prognostic factors such as age, stage of disease, treatment received and other socio-demographic and clinical characteristics. Appropriate adjustments will be made for multiple comparisons in the analysis of the PROMS data by adopting a more stringent cut-off for statistical significance.

The sample size calculations have been based on survival analysis methods. The 5-year figure has been used as the clinically relevant time point and assumes that recurrences before and after five years will be included in the analysis accordingly (i.e. patients will be followed from randomisation until it becomes impractical to do so further, and patients will only be censored in the analysis upon death or if lost to follow-up). Analyses will incorporate the time to an event as well as the occurrence of that event.

As this is a non-inferiority trial if there is high non-compliance with the test treatment groups then an analysis of only those compliant with the protocol will also be conducted.

The incidence of uncommon serious complications will be monitored.

Analyses of local tumour recurrence and of normal tissue effects adjusting for adjuvant therapy (chemotherapy, hormonal therapy) will be performed. Analyses of normal tissue effects will also be adjusted for breast size and surgical deficit.

Analyses will estimate the size of treatment effect with a confidence interval for the estimated difference between schedules. Information will be provided on both the

absolute and relative treatment effect. Each Test group will be compared with the Control group and treatment effects estimated separately. The inclusion of two test dose levels (Test 1 & 2) allows minor adjustment, for example by interpolation, between test dose levels to identify the fraction size most closely resembling the control schedule in terms of late change in breast/chest wall appearance and other adverse effects. The primary comparison is the rate of local tumour control at this 5-fraction dose level compared to the 15-fraction control. Since local relapse rates are so low, and no measurable difference in local relapse between the two test schedules is expected, interim analyses will also combine the test schedules for comparison with the control for the primary endpoint.

The primary outcome measure for the health economic evaluation will be the cost per quality-adjusted life year (QALY) gained from health resource usage and EQ-5D-5L health status. A decision analytic model will be used to extrapolate the trial results in order to estimate the QALYs and health resource utilisation over a lifetime time horizon, and to express the uncertainty in the estimates of cost-effectiveness. Information from published studies will be incorporated with the trial data to compare the trial regimens with the current UK standard regimen. In addition the economic evaluation will consider the impact of data relating to convenience of the treatment schedules (e.g. days of work missed, travel time and cost).

17.3 Sample Size

17.3.1. Main Trial

The target sample size is 4000 patients, with numbers balanced equally in each randomised group. This provides 80% power (1-sided $\alpha = 0.025$ to allow for 1-sided hypothesis and multiple testing) to exclude an increase of 1.6% in the 5-year local relapse rate between each test group and the control, assuming a 5-year rate of 2% in the 40.05 Gy schedule (using START data and allowing for reduction in local relapse due to recent adoption of aromatase inhibitors and trastuzumab). As local relapse rates after radiotherapy are low, there is limited potential for reducing this even further when comparing different regimens in a trial. Therefore the aim is to test whether the local relapse rate in the test groups is at least as effective, and not more than 1.6% higher than in the control group. Since no measurable difference in local relapse between the two test schedules is expected, interim analyses will combine the test schedules for comparison with the control for the primary endpoint. This combined analysis will enable an excess of 1.3% in the 5-year local relapse rate of the test groups relative to the control

to be excluded (80% power). As follow-up continues and more events accrue, the statistical power to compare each test schedule separately with the control will be higher. The calculations allow for up to 10% loss to follow-up / unevaluable.

17.3.2. Acute toxicity study I

The first 190 patients were entered into the acute toxicity sub-study to be assessed by a healthcare professional for acute skin toxicity up to settling of reaction to modified RTOG grade 1 and at least 4 weeks post radiotherapy. The patients were also asked to complete the Radiotherapy Breast Symptoms Diary Cards for self-assessment of acute toxicity. This would enable a rate of modified RTOG grade≥3 acute skin reactions of 10.9% to be excluded, based on the data from the 50 Gy in 25 fractions control schedule of the FAST trial. From the FAST trial 5-fraction test schedules, the rate of acute skin reactions was expected to be around 2.3% in the test groups of FAST-Forward. Using the Simon single stage design (using exact p-values) with power 89.2% and one-sided alpha of 7.9%, 50 patients per group were required (total 150). In each test group, if 3 or more patients developed grade ≥3 acute skin reactions using the modified RTOG scale, the IDMC may advise the Trial Steering Committee to consider a change in the test schedule.

This study was completed in summer 2012 and no grounds for undue clinical concern were reported. However, the data were collected in a way that would not allow the prospective differentiation between moist desquamation and oedema.

17.3.3. Acute toxicity study II

A second acute toxicity study will be conducted using standard CTCAE criteria to score erythema and moist desquamation (see section 8.2.1.). Fifty evaluable patients will be required for each treatment group (total approximately 150). An evaluable patient will be defined as receiving at least one fraction of radiotherapy and with complete or at most one missing toxicity assessment. This sample size will provide sufficient data to estimate the true incidence of acute skin reactions in the control and test schedules.

17.3.4. Photographic, patient reported outcome measures (PROMS) and health economics (HE) sub-studies

For the sub-studies (photographic assessments, PROMS and HE), 732 patients per group (2196 in total) will provide 80% power to detect an 8% difference in the prevalence

of late adverse effects at 5 years between the test groups (assuming a 5-year rate of 35%). PROMS and HE evaluation will be collected as part of the same booklet. Accrual will continue until there are 2196 evaluable patients in both the photographic and PROMS/HE sub-studies. It is also preferred that the patients in the photographic sub-study are the same subgroup as in the PROMS/HE studies, for data comparison. For the HE evaluation, it is expected that the majority of differences between the schedules in terms of quality-adjusted-life-years will be due to the late adverse effects, and so the estimated sample size will be sufficient. The uncertainty in HE outcomes will be reflected using probabilistic sensitivity analysis. The calculations allow for up to 10% loss to follow-up / unevaluable.

17.4 Interim analyses and Data Monitoring

The Independent Data Monitoring Committee (IDMC) reviewed the data on acute skin reactions on the first 190 patients. Following completion of the acute toxicity study I, the IDMC, together with the TSC, requested a second confirmatory study using the CTCAE scale. This study requires 150 evaluable patients (50 in each group), to provide a robust estimate of the true incidence of radiotherapy dose-dependent acute skin reactions in each of the treatment groups using a standard toxicity scale.

Interim analyses of local tumour control, normal tissue responses, radiotherapy side effects and the other endpoints will be conducted at yearly intervals and presented to the IDMC for confidential review. In the light of the interim analyses, the IDMC will advise the TSC if, in their view, the trial has indicated 'proof beyond reasonable doubt' that one of the schedules is clearly indicated or contraindicated in terms of local tumour control and/or normal tissue responses. In reviewing the evidence, the IDMC will also consider any available data from other randomised trials involving similar comparisons. The TSC may then consider modification or termination of the study. Unless such a situation arises, the Trial Management Group (TMG), the collaborators and the central administrative staff (except the statistician who prepares the analyses) will remain unaware of the interim results. The IDMC may recommend continuation beyond the planned number of patients in the main trial, the PROMS and Health Economics study or in the number of patients having photographic assessments, if it is felt that further information is required to address reliably the hypothesis in question.

18. ASSOCIATED STUDIES

At the time of randomisation all patients will be asked to consent to gift a whole blood sample which may be taken at any routine follow up visit, and a formalin-fixed paraffin-embedded (FFPE) diagnostic tumour tissue sample.

Sites will be notified by ICR-CTSU to when the sample collection will commence and no samples should be collected prior to this notification.

18.1 Molecular Correlates of Normal Tissue Injury

It is thought that part of the inter-patient variation in the incidence and severity of late normal tissue responses reflects inter-patient differences in tissue responsiveness to radiotherapy. Common DNA sequence variations (single nucleotide polymorphisms) account for differences in protein expression between individuals that may explain an important component of the variation between individuals. Genome-wide approaches offer scope to identify patterns of single nucleotide polymorphisms, DNA copy number and methylation status that may distinguish patients at lower and higher than average annual risk of late adverse effects.

Up to 20 ml of whole blood will be collected by venesection into blood tubes and sent to the Institute of Cancer Research, Sutton, Surrey, where it will be stored for future research, in accordance with the Human Tissue Act 2004. The research may be carried out at other centres, including those outside the UK. An aliquot of this blood may also be requested for comparison of genomic DNA with tumour DNA extracted from donated tissue samples (see 18.2). Blood will be collected at the treating hospital. Patients will also be asked to complete a family history questionnaire.

18.2 Molecular Correlates of Fractionation Sensitivity and Local Tumour Relapse

Local tumour relapse remains a clinical problem in a minority of women. The likelihood of local relapse may be influenced by genetically regulated factors, including the extent of intraductal spread and radiation resistance. Genome-wide approaches offer scope to identify DNA sequence differences (mutations and polymorphisms) between tumours that discriminate between patients who suffer a local relapse and those who remain disease-free. Relapses that occur close to the site of the primary tumour are assumed to be true local recurrences (sharing the same gene mutations), whereas those occurring elsewhere in the breast and often at a later point in time are assumed to be new primaries

(with differences in mutations compared to the primary tumour). Genomics offer scope for investigating the genetic relationships between ipsilateral and contralateral tumour relapse and primary tumour in a systematic way that may guide future local therapies. It is also possible to investigate loss of heterozygosity (LOH) in breast cancer by comparing DNA extracted from the tumour samples with DNA extracted from the blood samples (see 18.1). For LOH studies, a sample of the donated blood stored at the Institute of Cancer Research, Sutton, Surrey will be requested. It is proposed to establish tissue arrays and to extract DNA and RNA from paraffin blocks of primary tumours and ipsilateral and contralateral relapses/new primaries. Paraffin blocks containing the primary tumour and any subsequent recurrence/new primary from either breast will be sent to KCL/Guy's and St. Thomas' Hospital Breast Tissue Bank, London, where they will be stored for future analysis. In some centres, samples described above will be fresh frozen and sent to KCL/Guy's and St. Thomas' Hospital Breast Tissue Bank for the same analyses. The KCL/Guy's and St. Thomas' Breast Tissue Bank is a Human Tissue Authority licensed facility. After tissue cores and sections have been taken, the tumour paraffin blocks will be returned to the relevant pathology laboratory.

It is likely that breast cancers are heterogeneous in their sensitivity to fraction size. If so, it may be possible to distinguish subgroups of patients suited to treatment with large or small fractions based on examination of the tumour phenotype. Immunohistochemistry provides measures of tumour proliferation, hypoxia and DNA damage response status and other factors postulated to influence fractionation sensitivity. It is proposed to create tissue arrays from the primary tumour for future analysis of factors predicting sensitivity to radiotherapy fraction size.

18.3 Patient Reported Outcome Measures (PROMS) Study

The original protocol stipulated that the PROMS study would not be implemented until the acute toxicity study (I) had finished. PROMS is an umbrella term given to any data that are reported directly by the patient without an intermediary such as a family member or a healthcare professional [24]. In the present study, the PROMS measures of interest are the late-occurring normal tissue effects, quality of life and fatigue.

There is evidence that radiotherapy causes long-term effects on quality of life in terms of altered breast appearance, breast, arm and shoulder symptoms, as well as a possible impact on some general aspects such as fatigue. Results from the START trial have highlighted the value of patients' self-reported post–radiotherapy symptoms in

discriminating between radiotherapy (RT) regimens in favour of hypofractionation [21]. Experience of the START trials showed that patient-rated cancer specific PROMS data, obtained with the EORTC QLQ-C30 [16] provided useful data at baseline (for example concerning the effects of surgery) [25] and also made a small contribution to a comparison of the regimens up to 2 years, from which it was found that fewer changes in parameters were observed from 2-5 years (unpublished data 2010). The FAST-Forward PROMS sub-study is planned to provide subjective views of key breast symptoms and body image over 10 years of follow-up, thus to add supportive data in the comparison of a trade-off between local tumour control and adverse effects of treatment. The key effects of radiotherapy on PROMS are hypothesised to be on a range of breast symptoms as reported for the START trial [21] and potentially on body image plus short term general effects such as fatigue. Patient-reported arm and shoulder symptoms associated with RT are expected to be minimal since lymphatic RT is not allowed in the FAST-Forward trial. In the START trials these symptoms largely related to prior surgery [21].

The PROMS study is detailed in Appendix 4.

18.4 Health Economics (HE)

Rationale for HE measurement

The health economic analysis will make use of a generic, preference-based measure of HRQoL (health-related quality of life). The objective is to have an index measure of HRQoL where quality of life and absence of morbidity are valued on the same scale as quantity or length of life. This enables the calculation of quality adjusted survival where duration of time spent experiencing certain health states (e.g. receiving radiotherapy, experiencing a local recurrence) is weighted according to the HRQoL value associated with that health state [26,27]. The health benefits can then be combined with information on health resource usage in order to establish the cost-effectiveness of a 5-fraction schedule of curative radiotherapy in comparison to current UK practice. This is measured by using health resource usage questions and EQ-5D-5L (http://www.euroqol.org/about-eq-5d/valuation-of-eq-5d/eq-5d-5l-value-sets.html).

Timing of assessments

EQ-5D-5L and resource usage questions will be collected at: baseline, 3 and 6 months post radiotherapy and 1, 2, 5 and 10 years post randomisation.

The Health Economics study is detailed in Appendix 5.

19. TRIAL MANAGEMENT

19.1 Trial Management Group

A Trial Management Group (TMG) will be set up and will include the Chief Investigator, Chief Clinical Co-ordinators, ICR-CTSU Scientific Lead and identified collaborators, the Trial Statistician and the Trial Managers. Principal Investigators and key study personnel will be invited to join the TMG as appropriate to ensure representation from a range of centres and professional groups. Notwithstanding the legal obligations of the Sponsor and Chief Investigator, the TMG have operational responsibility for the conduct of the trial. Where possible membership will include at least one lay/consumer representative. The Committee's terms of reference, roles and responsibilities will be defined in a charter issued by ICR-CTSU and based on MRC Good Clinical Practice (MRC GCP).

19.2 Trial Steering Committee

A Trial Steering Committee (TSC) will be set up and will include an independent Chairman (not involved directly in the trial other than as a member of the TSC), not less than two other independent members, the Chief Investigator and one or two Principal Investigators. It is the role of the TSC to monitor progress of the trial and to ensure there is adherence to the protocol and the principles of Good Clinical Practice. The Committee's terms of reference, roles and responsibilities will be defined in a charter issued by ICR-CTSU and based on MRC GCP.

19.3 Independent Data Monitoring Committee

An IDMC will be instigated to monitor the progress of the trial. Membership of the IDMC will be proposed by the TMG and approved by the TSC. The Committee's terms of reference, roles and responsibilities will be defined in a charter issued by ICR-CTSU and based on MRC GCP. The IDMC should meet in confidence at regular intervals, and at least annually. A report of the findings and recommendations will be produced following each meeting and a summary of the minutes will be submitted to the TMG and TSC, and if required, the main REC.

The IDMC reserve the right to release any data on outcome or side-effects through the TSC to the TMG (and if appropriate to participants) if it determines at any stage that the combined evidence from this and other studies justifies it.

20. RESEARCH GOVERNANCE

20.1 Sponsor Responsibilities

The Institute of Cancer Research (ICR) is the agreed Sponsor of this study in accordance with the Research Governance Framework for Health and Social Care and the principles of Good Clinical Practice (GCP).

The following responsibilities have been delegated to:

The Chief Investigator:

- selection of Investigators
- taking appropriate urgent safety measures

The Chief Investigator or a named deputy delegated in his absence:

- prompt decision as to which related adverse events are related unexpected SAEs and prompt reporting of that decision to ICR-CTSU for onward reporting to the main REC

The Institute of Cancer Research (ICR-CTSU)

ICR-CTSU has overall responsibility for facilitating and coordinating the conduct of the trial and is also responsible for collating data obtained, and undertaking and reporting interim and final analyses.

The responsibilities of ICR-CTSU for the day-to-day management of the trial will include the following.

- ensuring an appropriate ethics opinion has been sought, and any amendments have been approved
- giving notice of amendments to protocol, make representations about amendments to the Main REC
- giving notice that the trial has ended
- randomising patients
- raising and resolving queries with local investigators
- issuing and collating PROMS questionnaires returned by post
- logging clinical and PROMS data received; raising queries
- keeping records of all serious adverse events (SAEs) reported by investigators
- notifying the Main REC and Investigators of related Serious Adverse Events

The Participating Centres

- putting and keeping in place arrangements to adhere to the principles of GCP
- keeping a copy of all 'essential documents' (as defined under the principles of GCP) and ensuring appropriate archiving and destruction of documentation once the trial has ended
- taking appropriate urgent safety measures

Centres wishing to recruit to this study will be asked to provide evidence that they can deliver protocol treatment. This will include the successful completion of the FAST-Forward QA programme (see Appendix 3).

Responsibilities are defined in an agreement between an individual participating centre and The Institute of Cancer Research, which must be signed and in place before recruitment can commence.

21. TRIAL ADMINISTRATION AND LOGISTICS

21.1 Protocol Compliance

The FAST-Forward trial is being conducted in accordance with the professional and regulatory standards required for non-commercial research in the NHS under the Research Governance Framework for Health and Social Care and the principles of GCP. Before activating the trial, participating centres are required to sign an agreement between an individual participating centre and The Institute of Cancer Research. Centres may commence recruitment once centre agreements have been signed by both parties, trial documentation is in place and a site initiation (visit or teleconference) has taken place. Site initiation visits will be conducted at centres where the Principal Investigator has requested one or where ICR-CTSU deems it is appropriate.

21.2 Protocol Amendments

Proposed protocol amendments will be submitted to the TMG by the Chief Investigator. The TMG will agree protocol amendments prior to acceptance and submission to the Main REC. Once approved the Principal Investigator at each centre will be informed of the change and sent all the associated documentation. It is the Principal Investigator's responsibility to submit amendments to their R&D department for approval. Confirmation that this has been done must be provided to ICR-CTSU.

21.3 Investigator Training

Training and advice will be provided via a trial launch meeting, training workshops, site initiation and QA feedback to identified key individuals in each participating centre by members of the Trial Management Group. Participating centres will be asked to maintain

a screening log to monitor randomisation acceptance rates, and additional support/training will be offered when lower than anticipated rates are encountered.

21.4 Data Acquisition

The clinical data should be recorded on the FAST-Forward case report forms (CRFs) and the relevant pages forwarded to ICR-CTSU in a timely manner. The Trial Management Group reserves the right to amend or add to the CRFs as appropriate. Such changes do not constitute a protocol amendment, and revised or additional forms should be used by centres in accordance with the guidelines provided by ICR-CTSU. Where appropriate, data may need to be collected retrospectively if an additional question has been added to the CRF.

By participating in the FAST-Forward trial, the Principal Investigators at each centre are confirming agreement with his/her local NHS Trust to ensure that

- sufficient data is recorded for all participating patients to enable accurate linkage between hospital records and CRFs
- source data and all trial related documentation are accurate, complete, maintained and accessible for monitoring and audit visits
- original consent forms are dated and signed by both patient and investigator and are kept together in a central log together with a copy of the specific patient information sheet(s) given at the time of consent
- all essential documents must be retained after the trial ends to comply with current legislation
- staff will comply with the protocol and Trial Guidance Notes for FAST-Forward

On receipt at ICR-CTSU, CRFs will be recorded as received and any missing forms will be reported to the originating centre. Illegible forms may be returned to the centre for clarification.

21.5 Central Data Monitoring

ICR-CTSU will review incoming CRFs for compliance with the protocol, and for inconsistent or missing data. Should any missing data or data anomalies be found, queries will be sent to the relevant centre for resolution. Following initial review, the CRF data items will be entered into the clinical study database held at ICR-CTSU.

Data will be further reviewed for data anomalies / missing data, by central statistical monitoring. Any systematic inconsistencies identified may trigger monitoring visits to centres.

21.6 On site Monitoring

If a monitoring visit is required, ICR-CTSU will contact the centre to discuss dates of proposed visit. Once a date has been confirmed, the centre should ensure that the relevant patient notes are available for monitoring.

If any problems are detected in the course of the monitoring visit, ICR-CTSU will work with the Principal Investigator to resolve issues and, if necessary, to determine the centre's future participation in the study.

ICR-CTSU staff conducting on-site monitoring will review essential documentation and carry out source data verification to confirm compliance with the centre agreement and trial protocol to ensure the protection of patients' rights as detailed in the Declaration of Helsinki 1964 as amended October 1996.

21.7 End of Study

The study end date is deemed to be the date of the last data capture and is expected to be at least 10 years after the last patient is entered.

21.8 Archiving

Essential documents are documents that individually and collectively permit evaluation of the conduct of the trial and substantiate the quality of the data collected. Essential documents will be maintained at ICR-CTSU in a way that will facilitate the management of the trial, audit and inspection. They should be retained for a sufficient period (at least 15 years) for possible audit. Documents should be securely stored and access restricted to authorised personnel.

Essential documents should also be archived at each participating centre in accordance with current legislation.

22. PATIENT PROTECTION AND ETHICAL CONSIDERATIONS

22.1 Risk Assessment

This study has been formally assessed for clinical risk using the ICR-CTSU risk assessment tool.

22.2 Patient Confidentiality

Patients will be asked to consent to their full name being collected at randomisation in addition to their date of birth, hospital number, postcode and NHS number (CHI in Scotland). This will allow tracing through the GP and national records to assist with long term follow up and to permit linkage with routinely collected NHS data. The personal data recorded on all documents will be regarded as confidential, and any information which would allow individual patients to be identified will not be released into the public domain.

Patients consenting to the PROMS and HE study are asked to provide their name, address and telephone number as well as the address and phone number of their GP to ICR-CTSU. These details will only be used for the purposes of the PROMS and HE sub-studies. The Principal Investigator must keep a separate log of patients’ trial numbers, names, and hospital numbers. The Principal Investigator must maintain in strict confidence trial documents, which are to be held in the local centre (e.g. patients' written consent forms). The Principal Investigator must ensure the patient's confidentiality is maintained.

ICR-CTSU will maintain the confidentiality of all patients and will not reproduce or disclose any information by which patients could be identified. Representatives of ICR-CTSU and the Radiotherapy QA team will be required to have access to patients notes for quality assurance purposes but patients should be reassured that their confidentiality will be respected at all times. In the case of special problems, it is also necessary to have access to the complete study records provided that patient confidentiality is protected.

22.3 Ethical Considerations

This trial has been approved by the South East Coast Kent Research Ethics Committee. Before entering patients, the Principal Investigator at each centre is responsible for gaining Site Specific Assessment and Research and Development approval for this study.

It is the responsibility of the Principal Investigator to give each patient, prior to inclusion in the trial, full and adequate verbal and written information regarding the objective and procedures of the trial and the possible risks involved. Sufficient time (a minimum of 24 hours) should be allowed for the patient to decide on trial entry. Patients must be informed about their right to withdraw from the trial at any time. Written patient information must be given to each patient before enrolment. The written patient information is an approved patient information sheet (PIS) according to national guidelines.

There are 4 separate PIS in this study

i) for centres who are taking part in all sub-studies

ii) for centres taking part in acute toxicity study I

iii) for centres taking part in acute toxicity study 2

iv) for centres who are not taking part in the PROMS and photographic sub-studies.

All PIS contain details of the collection of biological samples.

Patients will be encouraged to participate in the associated studies but if they decline, this will not exclude them from the trial.

All consent forms must be countersigned by the Principal Investigator or a designated individual. A record listing the designated individuals and the circumstances under which they may countersign consent forms must be clearly documented at the centre as part of the Delegation of Responsibilities Log. This log, together with original copies of all signed patient consent forms, must be available for inspection.

22.4 Data Sharing

Data arising from this research will be managed and made available to maximise public benefit. Data sharing will be in a timely and responsible manner. Appropriate regulatory permissions relating to the ethical use of data must be in place before the data can be shared.

22.5 Data Protection Act (DPA)

ICR-CTSU will comply with all aspects of the DPA 1998. Any requests from patients for access to data about them held at ICR-CTSU should be directed to the Trial Manager in the first instance who will refer the request to the Data Protection Officer at The Institute of Cancer Research.

22.6 Liability/Indemnity/Insurance

Indemnity for participating hospitals is provided by the usual NHS indemnity arrangements.

23. FINANCIAL MATTERS

The trial is investigator designed and led and has been approved by National Institute for Health Research Health Technology Assessment programme (NIHR-HTA) and meets the criteria for R&D support as outlined in the Statement of Partnership on Non-Commercial R&D in the NHS in England.

The trial has received funding from the NIHR-HTA. If further funding is received from any other source this will be made apparent in the patient information sheet and to the approving Main REC and NIHR-HTA, but will not require a protocol amendment.

The trial is part of the NIHR portfolio and NCRN (or regional equivalent) network resources should be made available for FAST-Forward specific research costs.

24. PUBLICATION POLICY

The main trial results will be published in the name of the trial in a peer-reviewed journal, on behalf of all collaborators. The manuscript will be prepared by a writing group, appointed from amongst the Trial Management Group and participating clinicians. All participating centres and clinicians will be acknowledged in this publication together with staff from the ICR-CTSU. All presentations and publications relating to the trial must be authorised by the Trial Management Group, on whose behalf publications should usually be made. Authorship of any secondary publications will reflect the intellectual and time input into these studies, and will not be the same as on the primary publication. No investigator may present or attempt to publish data relating to the FAST-Forward trial without prior permission from the Trial Management Group.

25. REFERENCES

1. Bates, T.D., *The 10-year results of a prospective trial of post-operative radiotherapy delivered in 3 fractions per week versus 2 fractions per week in breast carcinoma.* Br J Radiol, 1988. **61**(727): p. 625-30.
2. Whelan, T., et al., *Randomized trial of breast irradiation schedules after lumpectomy for women with lymph node-negative breast cancer.* J Natl Cancer Inst, 2002. **94**(15): p. 1143-50.
3. Yarnold, J., et al., *Fractionation sensitivity and dose response of late adverse effects in the breast after radiotherapy for early breast cancer: long-term results of a randomised trial.* Radiother Oncol, 2005. **75**(1): p. 9-17.
4. Owen, J.R., et al., *Effect of radiotherapy fraction size on tumour control in patients with early-stage breast cancer after local tumour excision: long-term results of a randomised trial.* Lancet Oncol, 2006. **7**(6): p. 467-71.
5. Bentzen, S.M., et al., *The UK Standardisation of Breast Radiotherapy (START) Trial A of radiotherapy hypofractionation for treatment of early breast cancer: a randomised trial.* Lancet Oncol, 2008. **9**(4): p. 331-41.
6. Bentzen, S.M., et al., *The UK Standardisation of Breast Radiotherapy (START) Trial B of radiotherapy hypofractionation for treatment of early breast cancer: a randomised trial.* Lancet, 2008. **371**(9618): p. 1098-107.
7. James, M.L., et al., *Fraction size for radiation treatment for breast conservation in early breast cancer.* Cochrane Database of Systematic Reviews, 2008(3).
8. Courdi, A., et al., *Long-term results of hypofractionated radiotherapy and hormonal therapy without surgery for breast cancer in elderly patients.* Radiother Oncol, 2006. **79**(2): p. 156-61.
9. Kirova, Y.M., et al., *Breast-conserving treatment in the elderly: long-term results of adjuvant hypofractionated and normofractionated radiotherapy.* Int J Radiat Oncol Biol Phys, 2009. **75**(1): p. 76-81.
10. Brunt, A.M., et al., *A 5-fraction regimen of adjuvant radiotherapy for women with early breast cancer: first analysis of the randomised UK FAST trial (ISRCTN62488883, CRUKE/04/015).* EJC Supplements. Presidential sessions late breaking and best of ECCO 15-ESMO 34 Abstracts, 2009. **7**(3): p. 2.
11. Huang, J., et al., *Does delay in starting treatment affect the outcomes of radiotherapy? A systematic review.* J Clin Oncol, 2003. **21**(3): p. 555-63.
12. Hebert-Croteau, N., et al., *Delay in adjuvant radiation treatment and outcomes of breast cancer--a review.* Breast Cancer Res Treat, 2002. **74**(1): p. 77-94.
13. Martin, S., et al., *Acute toxicity and 2-year adverse effects of 30 Gy in five fractions over 15 days to whole breast after local excision of early breast cancer.* Clin Oncol (R Coll Radiol), 2008. **20**(7): p. 502-5.
14. Bartelink, H., et al., *Impact of a higher radiation dose on local control and survival in breast-conserving therapy of early breast cancer: 10-year results of the randomized boost versus no boost EORTC 22881-10882 trial.* J Clin Oncol, 2007. **25**(22): p. 3259-65.
15. Early Breast Cancer Trialists' Collaborative Group (EBCTCG), Darby S, McGale P, Correa C, Taylor C, Arriagada R, Clarke M, Cutter D, Davies C, Ewertz M, Godwin J, Gray R, Pierce L, Whelan T, Wang Y, Peto R. *Effect of radiotherapy after breast-conserving surgery on 10-year recurrence and 15-year breast cancer death: meta-analysis of individual patient data for 10,801 women in 17 randomised trials.* Lancet. 2011 Nov 12;**378**(9804):1707-16. Epub 2011 Oct 19. Review.

16 Haviland JS et al., Evaluation of a method for grading late photographic change in breast appearance after radiotherapy for early breast cancer. Clin Oncol (R Coll Radiol). 2008 Sep;**20**(7):497-501.

17 Aaronson NK, Ahmedzai S, Bergman B, Bullinger M, Cull A, Duez NJ, Filiberti A, Flechtner H, Fleishman SB, de Haes JC, et al. *The European Organization for Research and Treatment of Cancer QLQ-C30: a quality-of-life instrument for use in international clinical trials in oncology.* J Natl Cancer Inst.1993 Mar 3;85(5):365-76.

18 Sprangers, M.A. and et al, *The European Organisation for Research and Treatment of Cancer Breast Cancer-Specific Quality-of Life Questionnaire Module: First results from a three-country field study.* J Clin Oncol, 2001. **5**(Classic Papers and Current Comments): p. 917-929.

19 Hopwood, P., et al., *A body image scale for use with cancer patients.* Eur J Cancer, 2001. **37**(2): p. 189-97.

20 Weis J, Arraras JI, Conroy T, Efficace F, Fleissner C, Görög A, Hammerlid E, Holzner B, Jones L, Lanceley A, Singer S, Wirtz M, Flechtner H, Bottomley A. *Development of an EORTC quality of life phase III module measuring cancer-related fatigue (EORTC QLQ-FA13).* Psychooncology. 2012 May 4. doi: 10.1002/pon.3092. [Epub ahead of print]

21 Hopwood P, Haviland JS, Sumo G, Mills J, Bliss JM, Yarnold JR; START Trial Management Group. *Comparison of patient-reported breast, arm, and shoulder symptoms and body image after radiotherapy for early breast cancer: 5-year follow-up in the randomised Standardisation of Breast Radiotherapy (START) trials*. Lancet Oncology 2010 Mar;11(3):231-40. Epub 2010 Feb 6.

22. *START-Standardisation of Breast Radiotherapy, trial protocol*. 1998.

23. *Surgical guidelines for the management of breast cancer.* Eur J Surg Oncol, 2009. **35 Suppl 1**: p. 1-22.

24. Patient Reported Outcome Measurement Group. *A structured review of patient-reported outcome measures (PROMs) for breast cancer.* Report to the Department of Health 2009 (University of Oxford)

25. Hopwood P, Haviland J, Mills J, Sumo G, Bliss J on behalf of the START Trial Management Group: *The impact of age and clinical factors on quality of life in early breast cancer: An analysis of 2200 women recruited to the UK START (Standardisation of Breast Radiotherapy) Trial*. Breast 2007;16: 241-51

26. Stein K et al. *Putting the 'Q' in quality adjusted life years (QALYs) for advanced ovarian cancer – An approach using data clustering methods and the internet.* European Journal of Cancer 2007; 43(1): 104-113

27. Kimman M et al. *Responsiveness of the EQ-5D in breast cancer patients in their first year after treatment.* Health and Quality of Life Outcomes 2009,7:11

APPENDIX 1: Acute skin reactions scoring scale

Modified RTOG scale (acute toxicity study I)

Grade	Description
Grade 0	No visible change
Grade 1	Faint/dull erythema
Grade 2	Tender/bright erythema +/- dry desquamation
Grade 3	Patchy moist desquamation, moderate oedema
Grade 4	Confluent moist desquamation, pitting oedema

CTCAE version 4.03 (acute toxicity study II)

Grade	Description
Grade 1	Faint erythema or dry desquamation
Grade 2	Moderate to brisk erythema; patchy moist desquamation, mostly confined to skin fold and creases; moderate oedema
Grade 3	Moist desquamation in areas other than skin folds and creases; bleeding induced by minor trauma or abrasion
Grade 4	Life threatening consequences; skin necrosis or ulceration of full thickness dermis; spontaneous bleeding from involved site; skin graft indicated
Grade 5	Death

APPENDIX 2: Selection of Test Dose Levels for FAST-Forward

Assuming i) that the fractionation sensitivity of late normal tissue effects (NTE) is well-described by an α/β value of 2.8, based on the results of the START A & FAST trials, ii) the slope of the dose response for NTE is well described by a γ value of 1.4, based on the START A trial and iii) complete repair of sublethal damage between daily fractions, the estimated equivalent total doses delivered in 2.0 Gy fractions assuming an α/β value of 2.8 Gy ($EQD_{2.8Gy}$) are shown below in a table that includes 50.0 Gy in 25 fractions as a reference schedule:

Fractionation regimen	$EQD_{2.8Gy}$ (Gy)	*ΔNTE (%)
50 Gy/25Fr/5Wk (2.0 Gy/Fr)	50.0	reference
40.05 Gy/15Fr/3Wk (2.67 Gy/Fr)	45.6	-12.3
27 Gy/5Fr/1Wk (5.4 Gy/Fr)	46.1	-11.1
26 Gy/5Fr/1Wk (5.2 Gy/Fr)	43.3	-18.8

* Negative values indicate estimated NTE rates lower than after 50 Gy in 25 fractions

Where tumour response is concerned, applying an α/β value of 4.6 Gy generated by the START pilot and START A trials and γ = 0.2 based on START A, the estimated equivalent total doses delivered in 2.0 Gy fractions ($EQD_{4.6Gy}$) are shown below in a table that includes 50 Gy in 25 fractions as a reference schedule:

Fractionation regimen	$EQD_{4.6Gy}$ (Gy)	*ΔTumour Relapse (%)
50 Gy/25Fr/5Wk (2.0 Gy/Fr)	50.0	reference
40.05 Gy/15Fr/3Wk (2.67 Gy/Fr)	44.1	+2.4
27 Gy/5Fr/1Wk (5.4 Gy/Fr)	41.0	+3.6
26 Gy/5Fr/1Wk (5.2 Gy/Fr)	38.6	+4.6

* Positive values indicate higher estimated levels of tumour relapse than after 50 Gy in 25 fractions

Note that a 2.4% excess tumour relapse rate was excluded with >97% confidence in START B, where the HR for local relapse after 40.05 Gy in 15 fractions compared to 50.0 Gy in 25 fractions was 0.79 (95% CI=0.48-1.29). In other words, the local relapse rate was, if anything, slightly lower, not higher, after 15 compared to 25 fractions [1]. If treatment time explains part

or all of this effect, local relapse in the 1-week schedules will be lower than those estimated above.

Acute skin reactions

Data on acute skin reactions in humans suggest that acute skin reactions will be milder in the test groups, since acute reactions are much less sensitive to fraction size than to total dose (which is reduced from 40.05 Gy to <30 Gy in the test groups). A 1 week schedule is too short to stimulate repopulation in the epidermis and radiosensitisation due to re-assortment. This expectation is consistent with the results of a pilot study in 30 patients receiving 30 Gy to whole breast in 5 fractions of 6.0 Gy over 15 days, in which there were 3 cases of grade 1 and 1 case of grade 2 moist desquamation (no cases of grade 3 or 4) [2].

Incomplete repair during a 24-hour inter-fraction interval

Turesson showed that a 24-hour inter-fraction interval is more sparing of late damage (telangiectasia) than a 4-hour interval. The difference was equivalent to 11% difference in fraction size [3]. Estimates of recovery half-time ($T_{½}$) for late endpoints in humans are based on the CHART head and neck trial: $T_{½}$ for telangiectasia was 3.8 hours and for fibrosis was 4.4 hours [4]. It is likely that repair beyond a 24-hours is very limited, and that no adjustment is needed to fraction size when moving from a 7-day to 1-day inter-fraction interval. The 2-year results of the FAST pilot study raised no concerns that an inter-fraction interval of 2 or 3 days leads to excessive late effects [2]. The $EQD_{2.8Gy}$ of the 5-fraction regimen delivered as one fraction per week in the FAST trial was estimated to be 54 Gy, but no marked change in breast appearance at 2 years was recorded in any of 30 patients treated with 30 Gy in 5 fractions over 15 days [2].. An element of incomplete repair at 24 hours (relative to 7 days) after 4 out of 5 test group fractions might lead to an estimated 1% increase in NTE, as illustrated for Test group 1 below if the second, third, fourth and fifth fractions follow on consecutive days and each deliver 5.5 Gy of absorbed dose instead of prescribed 5.4 Gy.

Fractionation regimen	$EQD_{2.8Gy}$ (Gy)	*ΔNTE (%)
5.4 Gy x 1	9.2	-
5.5 Gy x 4	38.0	-
5.4 Gy x 1 plus 5.5 Gy x 4	47.2	-10
** 5.4 Gy x 5	46.1	-11

* Estimated NTE rates relative to 50 Gy in 25 fractions
** Test group 2, assuming 100% repair between fractions

Despite a lack of evidence suggesting need for dose modification taking account of incomplete repair and/or a lower α/β value for late NTE than that estimated in the START A and FAST trials, a second test dose level (26.0 Gy in 5 fractions of 5.2 Gy) is included, as applied in START A. This allows interpolation, if required, in order to identify a 5-fraction schedule iso-effective with 40.05 Gy in 15 fractions.

Appendix 2 references

1. Bentzen, S.M., et al., *The UK Standardisation of Breast Radiotherapy (START) Trial B of radiotherapy hypofractionation for treatment of early breast cancer: a randomised trial.* Lancet, 2008. **371**(9618): p. 1098-107.
2. Martin, S., et al., *Acute toxicity and 2-year adverse effects of 30 Gy in five fractions over 15 days to whole breast after local excision of early breast cancer.* Clin Oncol (R Coll Radiol), 2008. **20**(7): p. 502-5.
3. Turesson, I. and G. Notter, *Accelerated versus conventional fractionation. The degree of incomplete repair in human skin with a four-hour-fraction interval studied after postmastectomy irradiation.* Acta Oncol, 1988. **27**(2): p. 169-79.
4. Bentzen, S.M., M.I. Saunders, and S. Dische, *Repair halftimes estimated from observations of treatment-related morbidity after CHART or conventional radiotherapy in head and neck cancer.* Radiother Oncol, 1999. **53**(3): p. 219-26.

APPENDIX 3: Quality Assurance Programme

1. Background

The complex nature of modern radiotherapy carries inherent problems both in ensuring reproducibility and accuracy within a radiotherapy unit and, more particularly, when carried out on a multi-centre basis. Specific issues in the treatment of the breast arise from the geometry of the treatment volume which varies in contour in all three planes with important radiation sensitive structures underlying the breast and chest wall including the lung and myocardium. Careful localisation, computerised planning, accurate verification of beam position and meticulous attention to alignment and matching during treatment are essential

A quality assurance programme is “a mandatory prerequisite when aiming at high dose, high precision radiotherapy” and is an integral component of any radiotherapy trial as defined by the EORTC guidelines for trial protocols in radiotherapy [1, 2].

In this multi-centre randomised trial the quality assurance programme will enable confirmation that technical guidelines within the protocol have been understood and implemented correctly by participants and that the dose prescription is delivered according to protocol together with appropriate documentation of technique and patient related data. This will ensure that clinical observations in terms of tumour control and normal tissue damage reflect differences in the randomised schedules rather that departures from trial protocol. Techniques used will be documented, this data will be available should differences in observed outcomes emerge.

In this way the definition of quality assurance as “all those planned and systematic actions necessary to provide adequate confidence that a produce will satisfy given requirements of quality” [3] can be satisfied and the scientific worth of the parent trial be validated.

The QA programme will build on that developed for the START and IMPORT trials. This has provided an element of consensus in radiotherapy technique amongst radiotherapy centres. FAST-Forward will necessitate the implementation of new technology in some centres where the use of intensity-modulated radiotherapy or image-guided radiotherapy has not been used previously.

2. Plan of investigation

The quality assurance programme will follow the guidelines set out by the EORTC [2] and will be co-ordinated by an experienced QA team based at Mount Vernon Hospital [4, 5]. It is based on anticipated accrual to around 20 centres over a three and a half to four year period. The programme will proceed as follows:

2.1 An initial questionnaire establishing precise details of technique to be used within the centre, together with specimen patient outlines to be used for ideal plans to be produced by each centre, where not already assessed for another trial.

2.2 A visit by the quality assurance team may be performed prior to a centre entering the study to validate independently the technique in use against the information given in the questionnaire. In particular, the following parameters will be assessed:

i) Target volume and treatment technique used.

ii) Confirmation of IMRT/compensator implementation.

iii) Planning of radiation distributions across the treatment volume for homogeneity and prescription points.

vi) Routine QC performed by the centre will be assessed and compared with current IPEM guidelines [6].

vii) Measurements across the treatment volume within a purpose-made phantom, if not performed for the same technique within the last 3 years.

viii) The imaging verification technique and protocol will be assessed.

2.3 All plans together with corresponding CT data sets will be collected electronically. Data should be anonymised with the patient's trial number and initials prior to sending to the QA team. Verification images will also be collected for the first 3 patients.

3. Quality control by department for IMRT

Where a centre has an established IMRT programme which has been previously credentialed by members of the NCRI trials QA team for another trial, some aspects of the FAST-Forward QA programme may be omitted. Where an established IMRT programme is not set up, additional QC may be required such as verification of fluence maps for each field.

4. Analysis of QA programme

The data from the quality assurance programme will be analysed separately from the main trial. Major discrepancies from trial protocol will be notified to participating centres. These will include:

i) Discrepancies in documentation, dose prescription and dose recording.
ii) Failure to meet upper and lower dose limits for treatment volumes.
iii) Systematic errors of technique in any stage of treatment from planning through to implementation.

The detailed analysis of the quality assurance data will produce quality information covering the following areas:

i) Variations in breast radiotherapy practice in participating centres
ii) A comparison of methods used for IMRT (multiple static fields, dynamic fields)
iii) An assessment of the emerging technologies and their quality control
iv) Quantification of dose uniformity during the treatment period
v) Correlation of physical parameters of radiation with trial outcomes:
    - The association between dose variation across the treatment volumes and tumour control.
    - Dose variation, machine energy and skin surface doses in relation to moderate/severe fibrosis and breast shrinkage.
    - Variations in dose homogeneity with rib pain, fracture and necrosis.

Appendix 3 references

1. Horiot, J.C., et al., *The programme of quality assurance of the EORTC radiotherapy group. A historical overview.* Radiother Oncol, 1993. **29**(2): p. 81-4.
2. Bolla, M., et al., *EORTC guidelines for writing protocols for clinical trials of radiotherapy.* Radiother Oncol, 1995. **36**(1): p. 1-8.
3. SMAC, *Quality Assurance in Radiotherapy*. 1991, Standing Committee on Cancer of the Standing Medical Advisory Committee.
4. Aird, E.G., et al., *Quality assurance in the CHART clinical trial.* Radiother Oncol, 1995. **36**(3): p. 235-44.
5. Venables, K., et al., *A survey of radiotherapy quality control practice in the United Kingdom for the START trial.* Radiother Oncol, 2001. **60**(3): p. 311-8.
6. IPEM, *81 Physics aspects of Quality Control in Radiotherapy*. IPEM Report, ed. W.P.M. Mayles, et al. 1999, York: IPEM.

APPENDIX 4: Patient Reported Outcome Measures (PROMS) Study

Patient reported outcome has become an important measure in breast cancer research over the past decade. It is an umbrella term coined for any subjective report from the patients on outcomes such as quality of life, self-perceived functional well-being and satisfaction of treatment received. There is evidence that radiotherapy causes long-term effects on quality of life in terms of altered breast appearance, breast, arm and shoulder symptoms, as well as a possible impact on some general aspects such as fatigue. Results from the START trial have highlighted the value of patients' self-reported post–radiotherapy symptoms in discriminating between radiotherapy (RT) regimens in favour of hypofractionation [1]. The START trials also showed that patient-rated cancer specific PROMS data, obtained with the EORTC QLQ-C30 [2] provided useful insight of patient experience at baseline (for example concerning the effects of surgery) [3] and made a small contribution to a comparison of the regimens up to 2 years, with fewer changes in parameters observed from 2-5 years (unpublished data 2010). The FAST-Forward PROMS sub-study is planned to provide subjective views of key breast symptoms and body image over 10 years of follow-up, with the aim to add supportive data in the comparison of a trade-off between local tumour control and adverse effects of treatment. The key effects of radiotherapy on PROMS are hypothesised to be on a range of breast symptoms as reported for the START trial [1] and potentially on body image plus short term general effects such as fatigue. Patient-reported arm and shoulder symptoms associated with RT are expected to be minimal since lymphatic RT is not allowed in the FAST-Forward trial. In the START trials these symptoms largely related to prior surgery [1].

Rationale for PROMS measurement

The evaluation strategy is based on standardised measures that will provide data and allow comparison with other relevant trials. The scales selected include specific measures for evaluating breast cancer therapies, body image, protocol-specific post RT symptoms, fatigue and psychological distress together with a general cancer health related quality of life scale; all have been used in the START and IMPORT radiotherapy trials. Assessment will be carried out over at least 5 years of follow-up.

Measures

The EORTC QLQ-C30 [2] comprises 5 functional sub-scales, 2 symptoms subscales and additional symptoms items and questions about global health and global quality of life.

The EORTC BR23 breast cancer module is a 23-item scale designed for use in breast cancer treatment [4]. It consists of 6 subscales: breast symptoms, arm symptoms, body image,

systemic side effects, sexual functioning, sexual enjoyment and items on hair loss and future perspective. This will be supplemented by 6 items specific to post-treatment effects evaluating change in skin appearance, change in overall appearance of the breast, breast shrinkage and hardening, position of the nipple and difficulty getting a bra to fit. An additional item measures shoulder stiffness.

The 10-item Body Image Scale (BIS) (of which 4 items are already incorporated in the BR23) was designed for use with cancer patients [5] and has been widely used in national breast cancer treatment trials.

The EORTC 13-item Fatigue module (EORTC QLQ-FA-13 (revised version Phase III) [6,7] will be used in this trial as detailed data are required to assess the short and longer term impact of RT. Relevant permission to use this scale has been obtained.

The PROMS evaluations are designed to complement the photographic assessments of breast appearance and clinical ratings of late normal tissue effects, and to capture the medium and long-term sequelae of breast radiation therapy on fatigue and psychological distress as important components of quality of life. The long-term PROMS sub-study is both comparative and descriptive: sample size considerations are addressed where appropriate.

The timing and mode of administration of PROMS questionnaires is based on experience from the START trials plus the need to assess adverse effects due to RT at an earlier time point (3 months). The PROMS data will be collected in a subset of centres participating in the FAST-Forward trial who wish to participate in the PROMS sub-study; this is the same strategy that was used in the START and IMPORT trials. All patients at PROMS participating centres will be invited to participate in the PROMS sub-study, but if they would prefer not to they may still be randomised into the main trial.

The PROMS outcomes will be summarised in a form that can be used by clinicians to inform patients and other stakeholders e.g. providers and commissioners of health care. No weighting will be given to prioritise any particular PROMS domain: the aim is to provide information from all PROMS domains as appropriate.

1) Normal tissue effects and body image

The proportion of patients suffering breast, arm and shoulder symptoms together with specific post-RT symptoms will be assessed at baseline, 3 and 6 months post-treatment and 1, 2, 5 and 10 years post-randomisation. Relevant symptoms from the breast cancer module (EORTC BR23) and protocol-specific post RT symptoms, all scored as 'quite a bit' or 'very much' will be used as an indicator of adverse effects. Body image concerns will be summarised for comparison between regimens, and where appropriate, individual items will also be compared.

2 General PROMS outcomes

1) The EORTC QLQ-C30 and the Fatigue module (EORTC QLQ-FA-13 revised Version Phase III) will be analysed according to EORTC guidelines and results compared between regimens for short and longer-term effects and differences.

2) Sexual function and sexual enjoyment (BR23)

Whilst we would not assume that these parameters are influenced primarily by RT, these domains are interrelated and may reflect the general impact of treatment. We will therefore be able to explore these domains within regimen and describe levels of dysfunction and distress across regimens. Formal statistical comparisons will be considered if differences emerge which warrant testing, but these are not expected. .

Summary of results to reflect favourable and unfavourable effects

In order to aid clinicians in an appraisal of the results we shall summarise the major findings, positive and negative, of the above outcomes. We will <u>not</u> attempt to produce a summary score representing a PROMS outcome for each regimen, but will report results for each domain under consideration. Results for medium and long-term effects will be presented in tabular form with accompanying explanatory paragraphs.

This will be a particularly important way of trying to provide a resume of a large study, which will help clinicians and others consider and discuss factors that influence a 'trade-off' of (psychosocial) cost and benefit, should this arise, the main one being considered to be enhanced cosmesis at a greater risk of local relapse.

Eligibility

All patients who:

- are entered into the FAST-Forward trial;
- are not taking part in a PROMS study as part of another trial;
- consent to be part of the PROMS sub-study and are available for follow up;

- are willing and able to complete the self-report PROMS questionnaires.

Sample Size

732 patients per group (total 2196) will provide 80% power to detect differences of ≥ 8% in the prevalence of specific normal tissue effects. Sample size estimate assumes a 2-sided significance level of = 0.025 (to allow for multiple testing) and allowing for 10% attrition due to illness or death (based on experience from the START trial).

The significance level chosen allows, to some degree, for the multiple testing involved in analysing individual sub-scales of the PROMS questionnaires. The numbers identified above also allow for some degree of attrition due to illness or death (10% non-completion). Experience from the START trial has shown compliance to be high. Particular care will be taken when approaching patients in the trial known to have relapsed, as although it is vital to collect these data, it may be requested at a sensitive point.

Patients will be stratified by centre and due representation geographically will be considered. The IDMC may recommend extending recruitment in the PROMS sub-study in all or a specific subgroup of patients. Such extension will take into account the attrition rate observed during follow-up in the study to date.

Timing of Assessments

The emphasis is on the long-term assessment of different treatment policies. Evaluation points are designed to allow comparison with the START and IMPORT LOW and HIGH trial PROMS outcomes.

Baseline Patient Reported Outcome Measures (PROMS): All measures: EORTC QLQ-C30 and BR23, protocol specific pre-RT items, Body Image Scale (BIS) and EORTC Fatigue module. A designated member of staff, trained in PROMS administration, should hand out questionnaires in the clinical centre. Patients will be asked to complete the questionnaires after a full explanation of the study and after giving informed consent but **before** the randomisation is known, to avoid the possibility of bias.

Patient Reported Outcome Measures (PROMS) Follow-up: Results of the START trials indicated a rise in breast symptoms at the 6-month evaluation and more precision is needed in estimating these effects closer to treatment. All PROMS measures (EORTC QLQ-C30 and BR23, Protocol specific post–RT breast symptoms, BIS, EORTC Fatigue module) will therefore

be mailed to patients from the FAST-Forward Trials Office at 3 and 6 months post radiotherapy, 1 and 10 years post randomisation. A smaller set of PROMS measures (EORTC BR23, BIS, protocol specific post-RT breast symptoms) will be mailed to patients from the FAST-Forward Trials Office at 2 and 5 years post randomisation.

Follow-up - general aspects of PROMS: administered by the Trials Office, will be made as follows:

Due care will be taken to check the physical status of all patients prior to questionnaire mailing. This will be done through email or telephone contact with the hospital department and/or GP as appropriate. The follow-up questionnaires will be sent out by the FAST-Forward Trials Office to the patients' home requesting completion within the week. If the questionnaires have not been returned 2 weeks after having been sent out, a letter will be sent to the patients enclosing another booklet requesting completion and return in the usual way. The follow-up assessments will be sent out shortly after the patient attends the hospital for routine annual follow-up, thereby ensuring that information on the patient's health status is up to date.

Missing data

All reasonable efforts will be made to ensure correct completion of the PROMS assessments. Full explanation of the PROMS study will be given by the responsible research nurse/member of breast care team prior to administration of the baseline questionnaires. On collection, the questionnaires will be briefly checked for completeness. The follow-up questionnaires will include instructions for completion. When individual items are missing, procedures, which have been used in similar studies, will be adopted:

- where the missing item is a single item measure this is simply recorded as a missing value;
- where the missing item forms part of a sub-scale a prorating procedure will be used depending on the total number of items on the scale and the number appropriately completed:
- where fewer than 50% of the items of the sub-scale have been completed correctly then this constitutes a missing case for that sub-scale;
- where at least 50% of the items of the sub-scale have been completed then the mean score obtained for the completed items can be inserted.

PROMS Study Management

Trials Office

The Study Co-ordinator, based in the FAST-Forward Trials Office, will be responsible for overall co-ordination of the study. The Co-ordinator will liaise closely with those responsible for the PROMS study in each participating centre and with the expert psycho-oncologist and clinicians involved in the project. The Co-ordinator will verify the status of the patient and send out the follow-up questionnaires. Any queries regarding the patient or the patient's management will be referred to the responsible person in the centre.

Centre

It is necessary for each participating centre to identify a person responsible for the conduct of the PROMS protocol. This person will explain the study to the patient, ensuring that the patient understands how to complete the PROMS questionnaire, and forward the first set of completed questionnaires to the Study Co-ordinator. He or she will maintain close liaison with the Study Co-ordinator in the FAST-Forward Trials Office and be responsible for organising cover in times of holiday or other planned absence.

PROMS Data Management

The Study Co-ordinator will be responsible for checking the data for consistency and completeness, for providing reminders for overdue questionnaires to the responsible persons in the centres and for entering the data onto the central database for the trial.

Statistical Analysis Plan

The algorithms developed for use with the PROMS questionnaires will be used to measure the parameters of interest. Groups of patients will be compared at agreed time points and overall for differences in these parameters [8]. The treatment groups will be compared at the individual time points with appropriate adjustments being made for multiple comparisons. Normal tissue effects will also be analysed using methodology developed for the START Trials i.e. survival analyses of time to occurrence of moderate or marked effects (scored ‘quite a bit’ or ‘very much’). Because of the longitudinal nature of the data, an analysis which takes into account the repeated measures is also needed. A generalised linear modelling approach will be adopted [9,10. This will allow the appropriate error distribution to be used and will enable the analysis to take account of important factors such as age, stage of disease, treatment received and other socio-demographic and clinical characteristics.

Informed Consent and Ethical Issues

Details for the main trial are outlined in section 20.1. The principal investigator or his/her delegated representative is responsible for obtaining each patient's signed informed consent prior to the administration of the baseline PROMS assessment.

Appendix 4 references

1. Hopwood P, Haviland JS, Sumo G, Mills J, Bliss JM, Yarnold JR; START Trial Management Group. *Comparison of patient-reported breast, arm, and shoulder symptoms and body image after radiotherapy for early breast cancer: 5-year follow-up in the randomised Standardisation of Breast Radiotherapy (START) trials*. Lancet Oncology 2010 Mar;11(3):231-40. Epub 2010 Feb 6.
2. Aaronson NK, Ahmedzai S, Bergman B, Bullinger M, Cull A, Duez NJ, Filiberti A, Flechtner H, Fleishman SB, de Haes JC, et al. *The European Organization for Research and Treatment of Cancer QLQ-C30: a quality-of-life instrument for use in international clinical trials in oncology.* J Natl Cancer Inst.1993 Mar 3;85(5):365-76.
3. Hopwood P, Haviland J, Mills J, Sumo G, Bliss J on behalf of the START Trial Management Group: The impact of age and clinical factors on quality of life in early breast cancer: An analysis of 2200 women recruited to the UK START (Standardisation of Breast Radiotherapy) Trial. Breast 2007;16: 241-51
4. Sprangers, M.A. and et al, *The European Organisation for Research and Treatment of Cancer Breast Cancer-Specific Quality-of Life Questionnaire Module: First results from a three-country field study.* J Clin Oncol, 2001. **5**(Classic Papers and Current Comments): p. 917-929.
5. Hopwood, P., et al., *A body image scale for use with cancer patients.* Eur J Cancer, 2001. **37**(2): p. 189-97.
6. Weis JB, Flechtner H, Bottomley A, Bredart A, Singer S, Holzner B, Larsson G, Jones L *"Development of an EORTC Module for the Assessment of Cancer Related Fatigue (EORTC FA 25; FAR 15)",* Quality of Life Research, (2005) Vol 14, No 9, pp 2035.
7. Weis J, Ya Gao, Conroy T, Singer S, Hammerlid E, Holzner B, Fleissner C, Efficace F, Arraras J, Bottomley A, Flechtner H, Lanceley A, Jones L*. Development of an EORTC module for the assessment of cancer related fatigue (EORTC FAR-15): Phase III results.* Quality of Life Research, ISOQOL Conference Toronto, Canada, Abstracts Issue October 2007*.*
8. Agresti, A., *A survey of models for repeated ordered categorical response data.* Stat Med, 1989. **8**(10): p. 1209-24.
9. McCullagh, P. and J.A. Nelder, *Generalised Linear Models*. 1989, London: Chapman & Hall.
10. Liang, K.Y. and S.L. Zeger, *Longitudinal Data Analysis using Generalised Linear Models.* Biometrika, 1986. **73**: p. 13-22.

APPENDIX 5: Health Economics (HE)

Rationale for HE measurement

The primary outcome measure for the health economic evaluation will be the cost per quality-adjusted life year (QALY) gained informed from health resource usage and EQ-5D-5L health status. The objective of the health economic evaluation is to establish whether a 5-fraction schedule of curative radiotherapy is cost-effective relative to current UK practice.

The health economic analysis will make use of a generic, preference-based measure of HRQoL (health-related quality of life). The objective is to have an index measure of HRQoL where quality of life and absence of morbidity are valued on the same scale as quantity or length of life. This enables the calculation of quality adjusted survival where duration of time spent experiencing certain health states (e.g. receiving radiotherapy, experiencing a local recurrence) is weighted according to the HRQoL value associated with that health state [1, 2].

Short term study

All patients in the PROMS study will complete the EQ-5D-5L assessment at 3 months in order to compare HRQoL 'off treatment' between treatment groups. This would provide information about how soon HRQoL may improve following radiotherapy and provide information on whether any differences between treatment arms persist beyond the end of treatment.

Long term study

Comparison of HRQoL at one year would provide information to test the assumption that there are no long-term differences in HRQoL directly resulting from different radiotherapy fractionation schedules. Repeated follow-up of EQ-5D-5L could potentially be used to estimate the impact of ever having had a long-term adverse event and/or recurrence on HRQoL. However, as recurrences are likely to be rare, data on the HRQoL impacts of recurrence may be supplemented or obtained from literature review. Such published data may be limited for the long-term adverse events associated with radiotherapy.

Resource use data

The aim of this part of the HE evaluation would be to compare the treatment groups in terms of resource implications for the NHS. This would primarily entail comparing the costs of providing each radiotherapy regimen and the costs of treating adverse events (short and long term) and further breast cancer events. The trial may well detect hospitalisations associated with adverse events or further breast cancer events. Questions relating to resource use outside of inpatient care, such as the use of specialist nurses, GP visits, medication and outpatient visits for treating adverse events and side effects from treatment will be added to the PROMS questionnaires,

as in the IMPORT LOW trial. Information on the resource use associated with recurrent breast cancer events may be supplemented or obtained from literature review. These data may be limited for long-term adverse events associated with radiotherapy. In previous studies expert opinion has been utilised to provide estimates of the resource use that would typically be associated with common adverse events, which is then applied to the number of such events observed in the trial.

Measures

The **EQ-5D-5L** is a standardised instrument designed for self-completion in an adult population [3]. The questionnaire asks patients to describe their current health status by specifying one of five levels of severity (no problems, slight problems, moderate problems, severe problems and unable to/extreme problems) across five dimensions (mobility, self-care, usual activities, pain/discomfort, anxiety/depression). The preference based health related quality of life weights for each of the health states described by the EQ-5D-5L are currently being estimated and will be available before the end of the trial. Currently EQ-5D-5L health states can be valued using a cross-walk algorithm from a set of preferences established on the basis of a UK general population survey [4].

The resultant EQ-5D-5L scores will be compared between each regimen at each time point and will inform the health economic analysis in calculating quality adjusted survival for patients receiving each regimen. Where appropriate, the EQ-5D-5L scores will be subject to regression analysis to identify the impact on health related quality of life of having experienced an adverse event or recurrence. In order to extrapolate beyond the trial these data will be supplemented by a literature search of previous studies of the health related quality of life impact of treatment and events in patients with breast cancer.

The EQ-5D-5L will enable the calculation of quality adjusted life years that would be consistent and comparable with those routinely used in the economic evaluation of health care technologies by the National Institute for Health and Clinical Excellence in the UK [5].

Resource use questions:

1. How many times have you **<u>been visited</u>** by your GP for any reason (even if not related to your breast cancer)?
2. How many times have **<u>you visited</u>** your GP for any reason (even if not related to your breast cancer)?
3. How many times have you **<u>been visited</u>** by a district nurse?
4. How many times have you **<u>been visited</u>** by a MacMillan nurse?
5. How many days have you spent in hospital **<u>related to your breast cancer</u>**?

6. How many days have you spent in hospital **for other reasons**?
7. How many hospital outpatient visits have you had **related to your breast cancer**?
8. How many hospital outpatient visits have you had **for other reasons**?

Timing of assessments

EQ-5D-5L and resource used questions will be collected at: baseline 3 and 6 months post radiotherapy and 1, 2, 5 and 10 years post randomisation.

Methods

A decision analytic model describing a series of health states and health events experienced by patients with early breast cancer will be developed [6]. This model will be used to synthesise information from the trial and other published studies in order to estimate costs and quality adjusted survival over an appropriate time horizon from the perspective of the UK NHS and PSS. Uncertainty around the values used in the decision analytic model will be characterised using probabilistic sensitivity analysis. The trial regimens will be evaluated using standard cost-effectiveness analysis. If one strategy is not found to be dominant (i.e. less costly and more effective) in comparison to the other, then an incremental cost-effectiveness ratio (ICER) will be determined [7]. The ICER will be based on the mean costs and mean QALYs estimated within the probabilistic sensitivity analysis of the decision model. Uncertainty around cost-effectiveness will be described using cost-effectiveness acceptability curves which describe the probability that an intervention is cost-effective.

Appendix 5 references

1 Stein K et al. *Putting the 'Q' in quality adjusted life years (QALYs) for advanced ovarian cancer – An approach using data clustering methods and the internet.* European Journal of Cancer 2007; 43(1): 104-113
2. Kimman M et al. *Responsiveness of the EQ-5D in breast cancer patients in their first year after treatment.* Health and Quality of Life Outcomes 2009,7:11
3. Herdman M et al. Development and preliminary testing of the new five-level version of Eq-5D (EQ-5D-5L). Quality of Life Research 2011; 20(10): 1727-36
4. http://www.euroqol.org/about-eq-5d/valuation-of-eq-5d/eq-5d-5l-value-sets.html
5. National Institute for Health and Clinical Excellence. Guide to the methods of technology appraisal. London: NICE, 2008.
6. Briggs A, Sculpher M, Claxton K. Decision modelling for Health Economic Evaluation. Oxford University Press, 2006. Oxford.
7. Drummond M, Sculpher M, Torrance G. Methods for the Economic Evaluation of Health Care Programs. Oxford University Press, 2005. Oxford.

FAST-Forward

(Randomised clinical trial testing a 1-week course of curative whole breast radiotherapy against a standard 3-week schedule in terms of local cancer control and late adverse effects in patients with early breast cancer)

Planning Pack

for the FAST-Forward Trial

Version 3

A guide to outlining, planning & verifying

FAST-Forward patients

FAST-Forward Trial Management Group
Modified May 2013

CONTENTS

CONTENTS 80
1 INTRODUCTION..81
2 LOCALISATION..81
3 OUTLINING..83
3.1 Target Volumes 83
3.1.1 Tumour Bed 83
3.1.2 Whole Breast and Chest Wall 85
3.1.2.1 Volume-based planning 85
3.1.2.2 Field-based planning 86
3.2 Organs at Risk..91
4 TREATMENT PLANNING 94
4.1 Dose Prescription 94
4.2 Dose Targets and Constraints..94
4.2.1 Target Volumes 94
4.2.2 Dose Constraints for Organs at Risk..95
4.3 Bolus Use 96
4.4 Whole Breast and Chest Wall 96
4.4.1 Photons...96
4.4.2 Electrons...99
4.5 Tumour Bed Boost 99
4.5.1 Photons...99
4.5.2 Electrons...101
5 RADIOTHERAPY TREATMENT VERIFICATION 102
5.1 Set-up Verification – Breast and Chest Wall 102
5.1.1 Control Group 102
5.1.2 Test group 1 and 2..102
5.2 Set-up Verification – Tumour Bed Boost..102
5.2.1 Photon Boost 102
5.2.2 Electron Boost...103
5.3 In-vivo Dosimetry..103
6 TREATMENT GAPS 103

1 INTRODUCTION

This document should be used as an accompaniment to the FAST-FORWARD trial protocol by all clinicians, physicists, radiographers and dosimetrists involved in the planning and treating of FAST-FORWARD patients. It provides basic guidance on localisation, outlining, planning and treatment verification. However, it should be noted that the planning methods are not intended to be entirely prescriptive – it is hoped that they will provide a good starting point from which each centre may decide to develop its own technique to meet the planning aims. Please feel free to discuss any aspect of this planning pack with the FAST-Forward QA team.

2 LOCALISATION

Delineation of the tumour bed is strongly recommended for all patients who have had breast conserving surgery as this facilitates appropriate placement of the tangential breast field to maximise target coverage whilst minimising dose to organs at risk (OAR).

To assist the delineation, it is strongly advised that titanium clips or gold seeds are implanted into the walls of the tumour excision cavity (tumour bed) at the time of breast conserving surgery as per British Association of Surgical Oncology (BASO) guidelines [1].

It is recommended that pairs of clips/seeds are positioned at the medial, lateral, superior, inferior, anterior and posterior margins of surgical resection. Placing the clips in pairs as shown in Fig. 1 ensures that any clip migration will be evident by the visualisation of single clips [1].

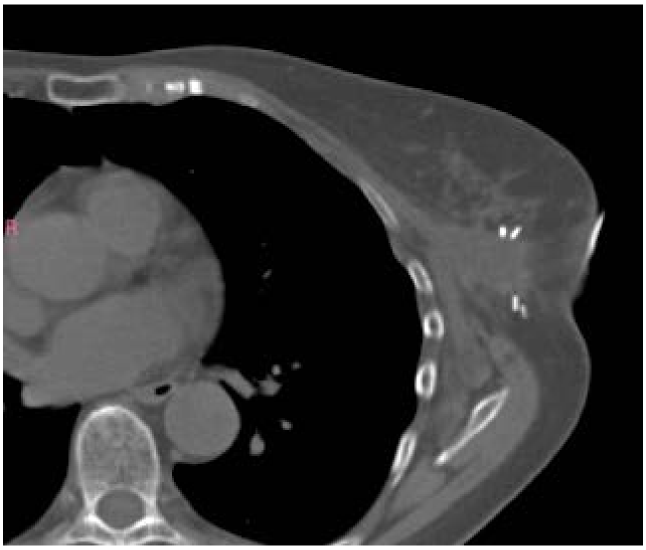

Fig. 1: CT slice showing 2 pairs of titanium clips implanted around the tumour bed

The tumour bed may be localised if there is a well-defined seroma in the absence of implanted markers. At least one of these localisation methods will be necessary if the boost radiotherapy is to be delivered with a conformal photon plan. For more detailed guidance, please refer to the IMPORT surgical clips protocol.

The same method of localisation, outlining and planning must be adopted for all trial groups.

3 OUTLINING

3.1 Target Volumes

It is compulsory to outline target volumes and the relevant organs at risk for radiotherapy planning of FAST-Forward patients. A summary is presented in Table 1. Outlining the boost is mandatory only for patients that are to receive tumour bed boost treatment.

Table 1: Summary of radiotherapy planning volumes and margins

	CTV	PTV
**Whole Breast (WB)**	CTV_{WB} = Soft tissues of the whole breast, 5 mm below the skin surface	PTV_{WB} = CTV_{WB} + 10 mm margin
**Chest Wall (CW)**	CTV_{CW} = Skin flaps and soft tissues	PTV_{CW} = CTV_{CW} + 10 mm margin
**Boost**	CTV_{TB} = tumour bed	PTV_{TB}= CTV_{TB} + 10 mm margin

3.1.1 Tumour Bed

CTV_{TB} is outlined by drawing around the implanted markers and any changes in the surrounding tissue architecture (Fig. 2). For patients with no visible seroma centres should consider contouring around the clips and adding a 5 to 10 mm margin to it to obtain the CTV.

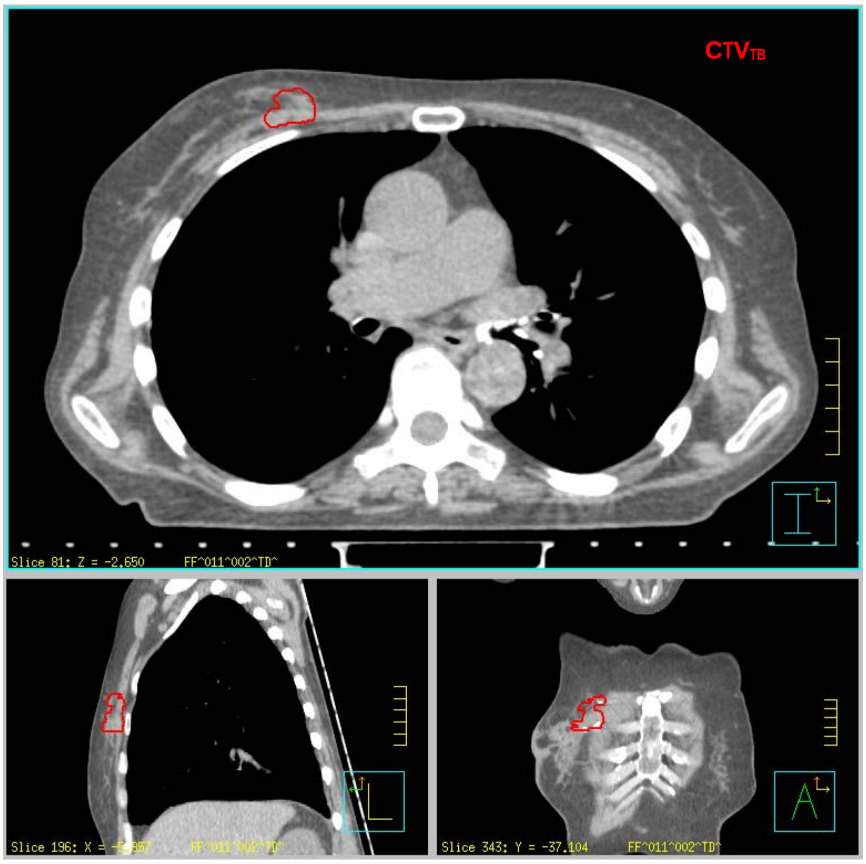

Fig. 2: Tumour bed CTV in axial, sagittal, and coronal planes

This is grown by 10 mm to give the tumour bed PTV or PTV_{TB} (Fig. 3). When planning the tumour bed boost, the treatment fields should be positioned to cover the unmodified PTV_{TB} with an appropriate margin for penumbra.

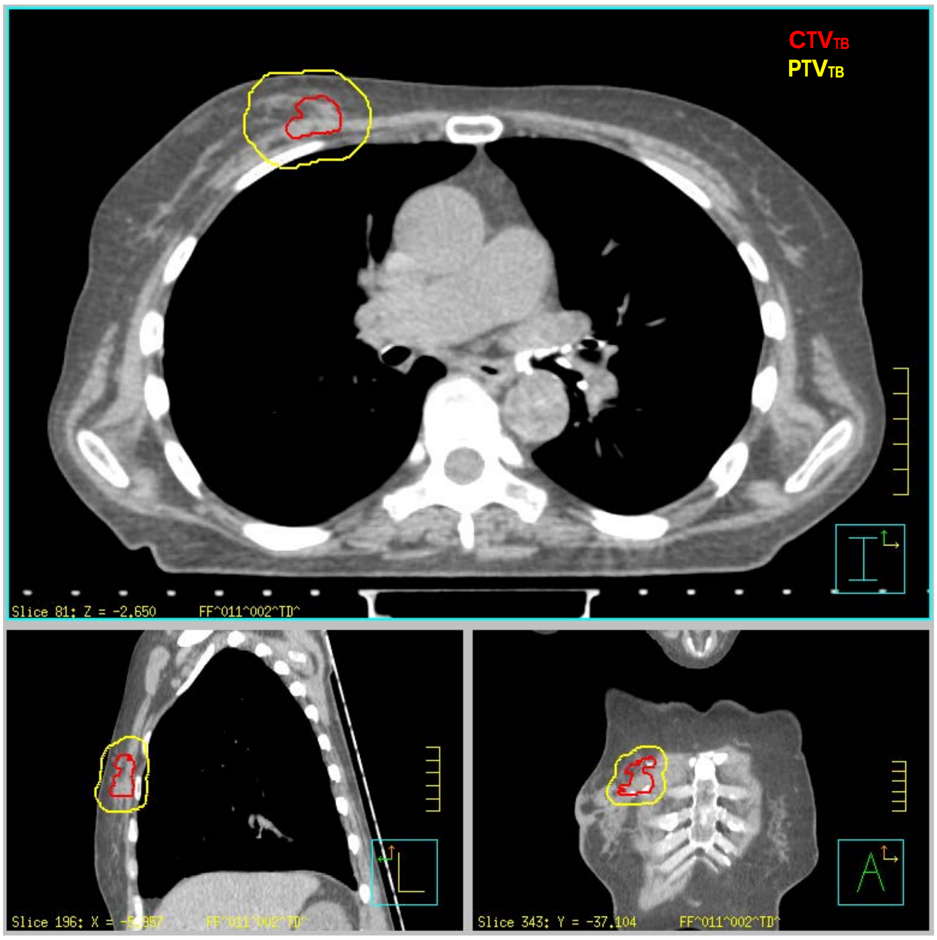

Fig. 3: Axial slices showing tumour bed PTV on central axis

For reporting purposes only the PTV is then modified 5 mm inside the skin surface and 5 mm around the lung (Fig. 4). This structure is denoted as PTV_{TB} DVH.

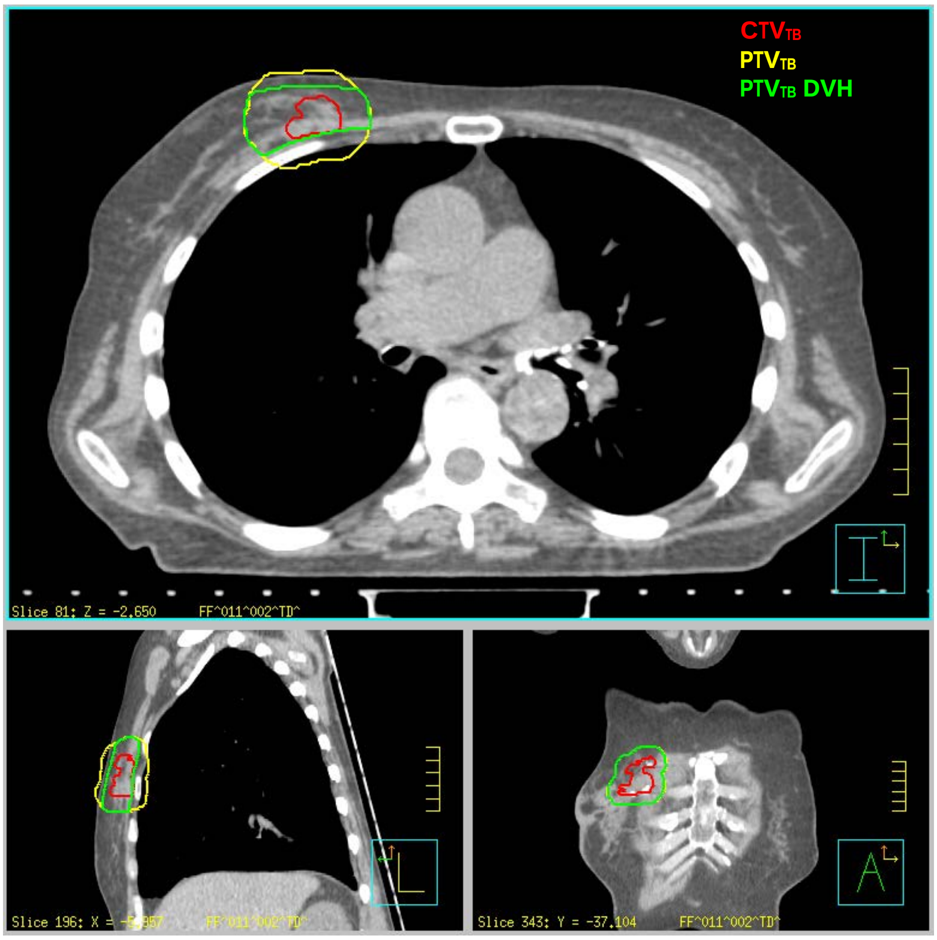

Fig. 4: Axial slice showing modified tumour bed PTV

3.1.2 Whole Breast and Chest Wall

3.1.2.1 Volume-based planning

The Whole Breast CTV is based on the recommendations in the START trial protocol. The CTV includes the soft tissues of the whole breast from 5 mm below the skin surface down to the deep fascia, excluding muscle and underlying rib cage. The posterior margin should not extend beyond the deep fascia (unless clearly breached by the tumour). If the anatomy of this region cannot be easily visualised, the posterior margin should be limited to 5 mm anterior to the lung/chest wall interface. CTV_{WB} should not extend beyond the edges of the visible/palpable breast in medial and lateral directions. A 10 mm margin is added to create the Whole Breast PTV (PTV_{WB}). The treatment fields should be positioned to cover the unmodified whole breast PTV with an appropriate margin for penumbra.

This PTV_{WB} is then cropped 5 mm inside the skin and 5 mm from the lung surface for dose reporting purposes only. This structure is denoted as PTV_{WB} DVH.

3.1.2.2 Field-based planning

When outlining a CTV volume on each axial slice it can be very difficult to accurately delineate breast tissue on X-ray CT images, and this can result in an overestimate of the whole breast volume, especially in obese patients. An alternative strategy is to generate a field-based structure which is not a true PTV, but is helpful for reporting purposes. A provisional tangential field pair is selected to cover the breast tissue and minimise dose to the normal tissues, by scrolling up and down the CT dataset. The whole breast field based PTV is then generated according to the following criteria, as illustrated on the images below:

- 5 mm from the skin surface
- 5 mm from the lung/chest wall interface
- 5 mm from the posterior beam edge (or the MLC if it is used on the main beams)
- 10 mm from the superior and inferior beam edges

The margins of the field-based PTV from the superior and inferior field borders are valid under the assumption that the isocentre of the plan has been placed in the central region of the target. For a mono-isocentric technique or if divergence has been removed from the superior edge using floor twist please contact the QA team for guidance.

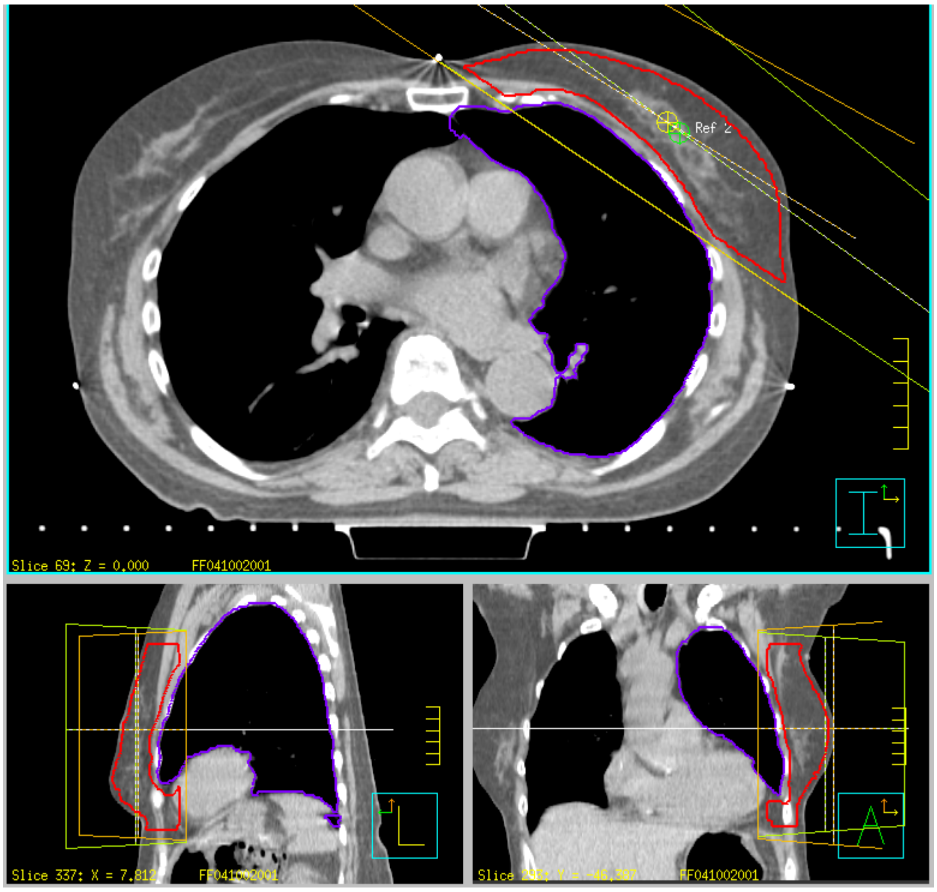

Fig. 5: Field-based whole breast PTV in axial, sagittal, and coronal planes

The Chest Wall CTV encompasses the skin flaps and includes the soft tissues down to the deep fascia, excluding the underlying muscle and rib cage. A 10 mm margin is added to create the Chest Wall PTV. This PTV is then cropped 5 mm inside the skin or along the skin (depending on the centre's practice of bolus use) and 5 mm from the lung surface for dose reporting purposes. This structure is denoted as PTV_{CW} DVH.

The same field based PTV principle listed above can also be applied to chest wall patients in the trial as shown in Fig. 6. In case of a very thin chest wall when the field-based PTV_{CW} generated according to the procedure outlined above does not represent the irradiated volume adequately, contact the QA team for advice.

If it is the centre's policy to use bolus for part of the treatment fractions, the bolus and no bolus plans should be evaluated and submitted separately, and two dose reporting structures should be created: one cropped 5 mm inside the skin for the no bolus plan, and one including the skin for the bolus plan. Target dose constraints should be met for both structures individually.

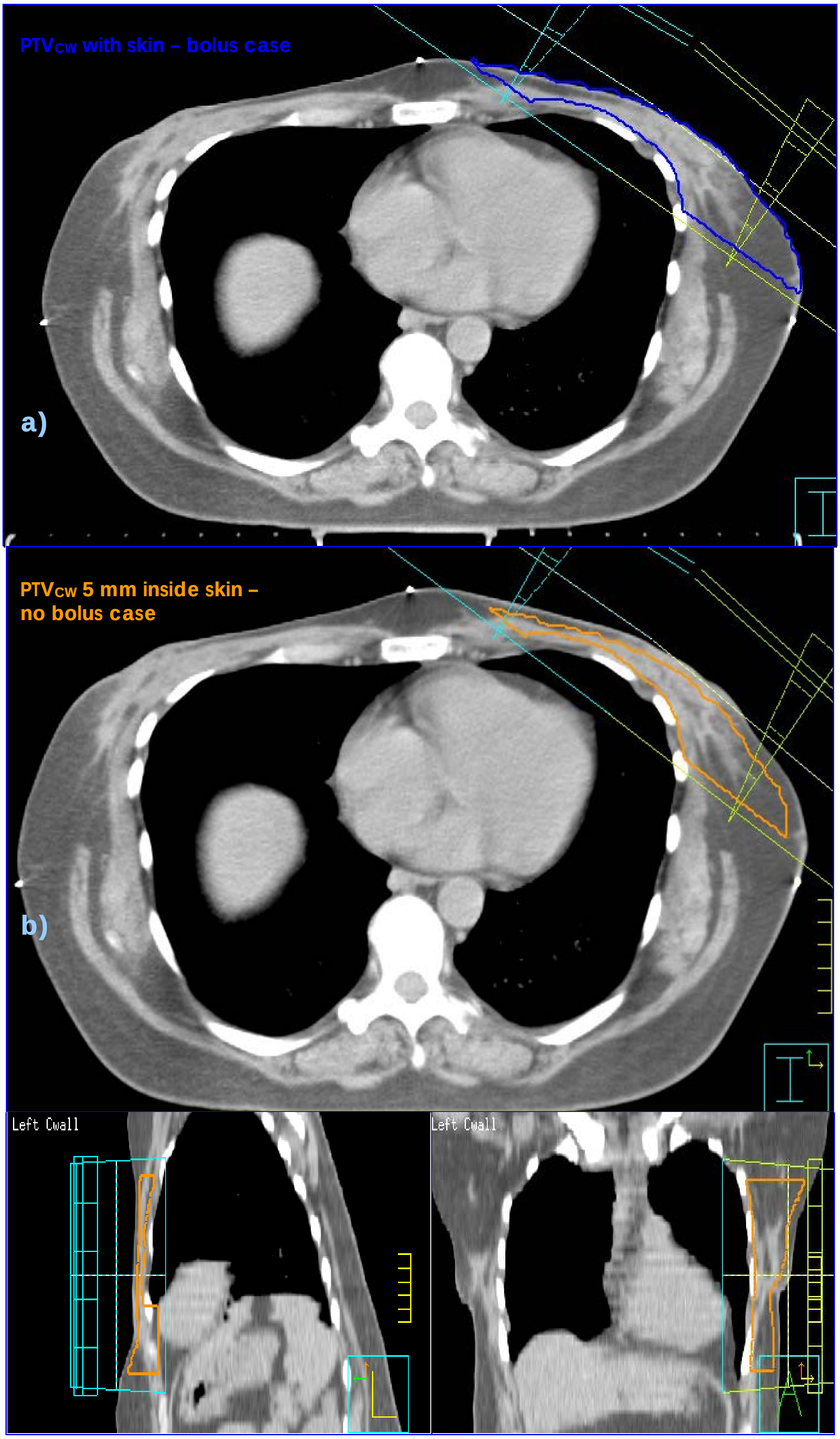

Fig. 6: Field based chest wall PTV with (a) and without bolus (b)

It is often necessary to use the MLC to shield organs at risk in the treatment beams and this is recommended, provided that the tumour bed is away from the shielded area. In cases like this, when the MLC is used on the main tangential beams, the field-based reporting structure should be contoured 5 mm inside the MLC instead of 5 mm inside the field border. The example on Fig. 7 shows a left side patient with MLC shielding for the heart; the same can be done to shield parts of the lung or the liver in right side plans.

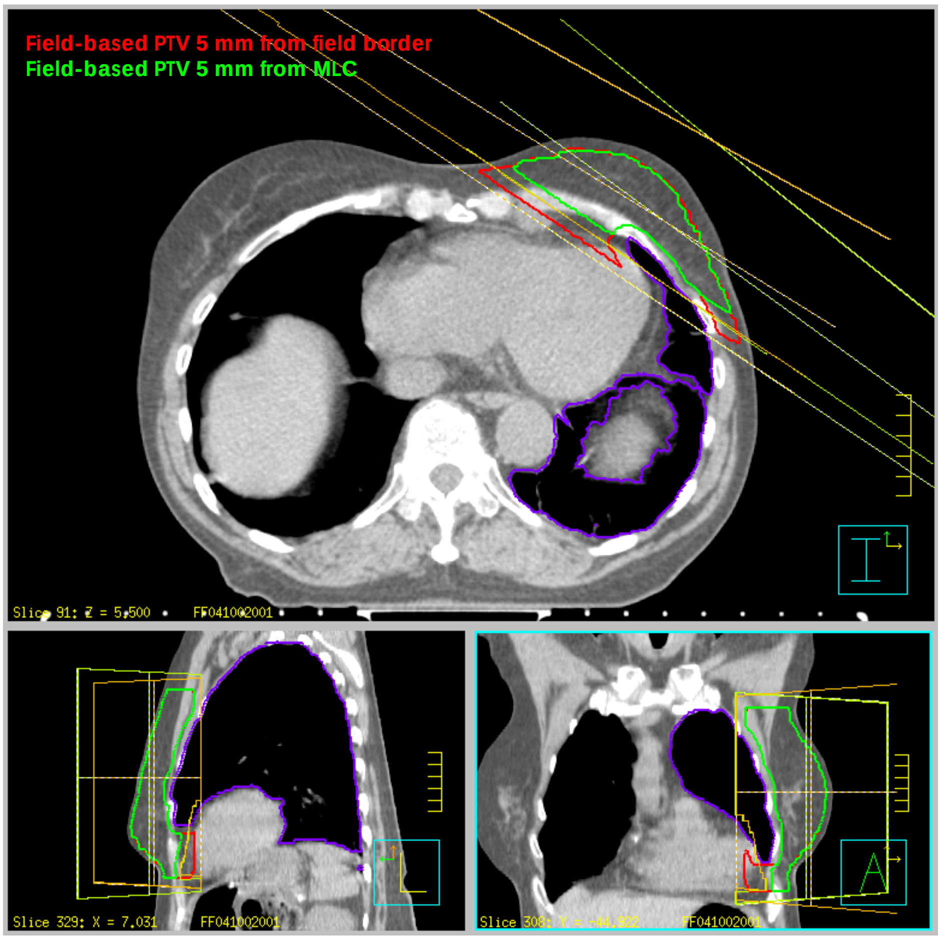

Fig. 7: Field-based PTV in case of MLC shielding for the heart.

The achieved target and OAR dose constraints should in this case be reported for the modified field-based PTV structure.

Once created according to the criteria outlined above, the field-based PTV must not be modified any further. It will sometimes include tissues that are not breast in the

superior and inferior region of the target (Fig. 8); in view of the consistency of the dose reporting structure these should not be manually cropped out.

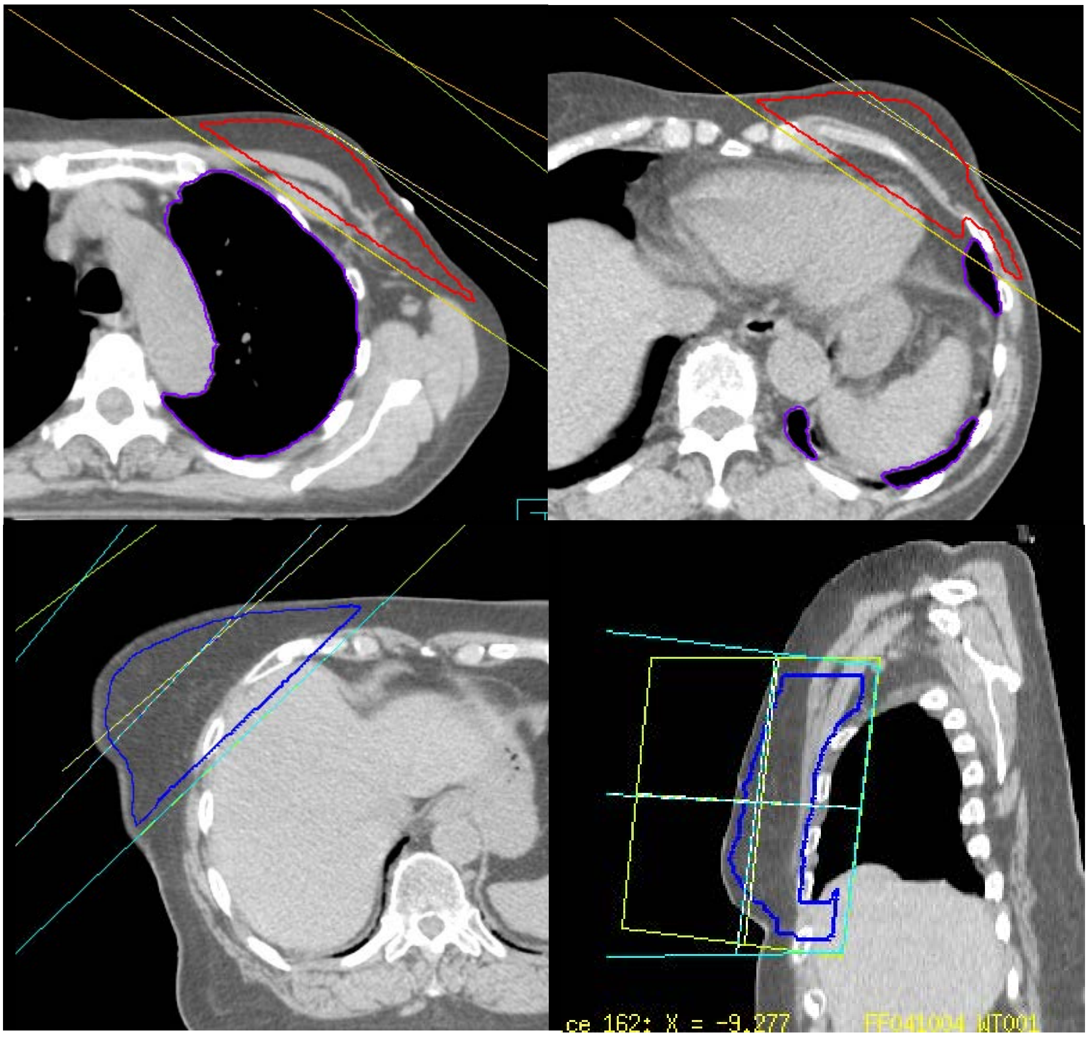

Fig. 8: Non-breast tissue included in the dose reporting structure

Whenever possible, non-breast tissue should be excluded from the treatment fields or shielded using the MLC, as long as the primary tumour site is away from the shielded area.

3.2 Organs at Risk

It is mandatory to contour the ipsilateral lung and heart for dose volume histogram assessment (Fig. 9).

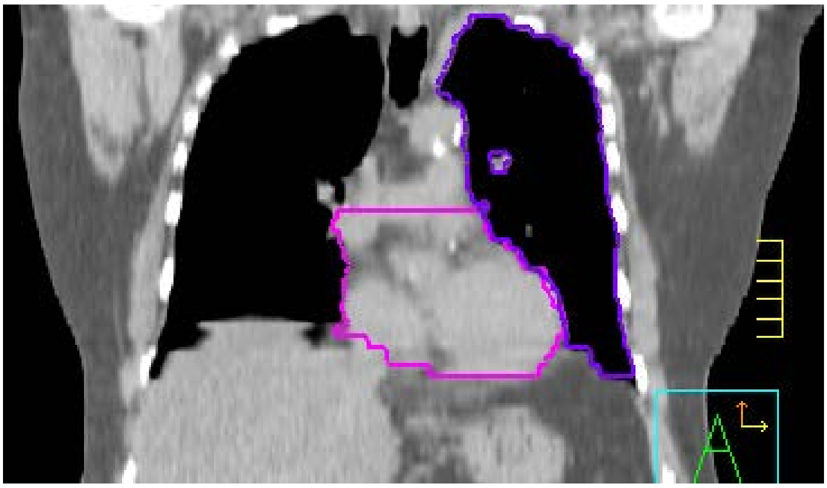

Fig. 9: Delineation of organs at risk: ipsilateral lung and heart in axial and coronal planes

The ipsilateral lung must be outlined as a single structure, and care should be taken not to include any air ways or major blood vessels. The scanning area must cover the entire lung volume.

The following guidelines for heart delineation have been adapted from the Wales Cancer Trials Unit SCOPE1 Radiotherapy Treatment Planning and Delivery Document: leads - T Crosby, J Staffurth and L Wills.

'The whole heart should be outlined to the extent of the pericardial sac (if visible). The major blood vessels (superior to the organ) and the inferior vena cava (towards the inferior extent of the heart) are excluded. The superior extent is often difficult to define and may be simplified by identification of the vessels superior to the heart. Use the point where the pulmonary trunk and the right pulmonary artery are seen as separate structures as indication of the superior extent of the heart.

Shown below in Fig. 10 are alternate CT images for a scan taken at 0.3 cm intervals.

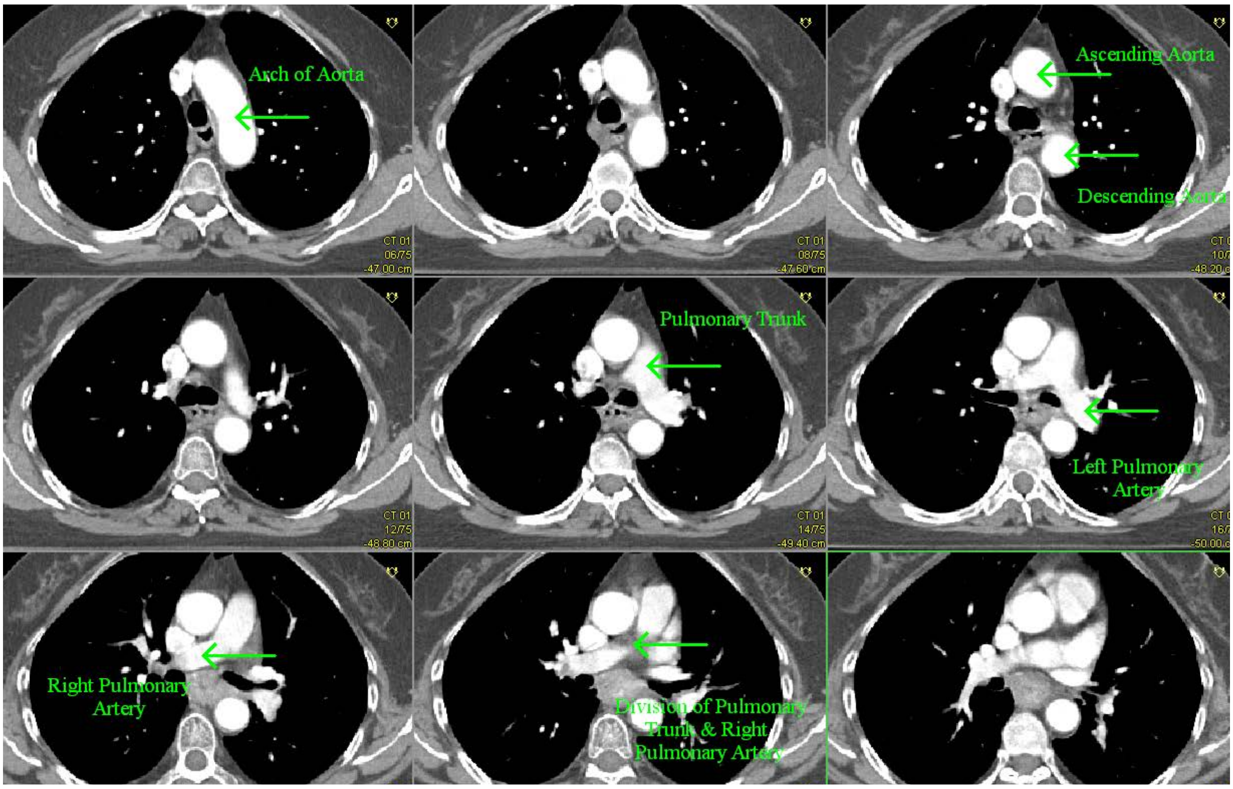

Fig. 10: Alternate 0.3 cm CT slices indicating cardiac anatomy

The definition of the heart is shown in Fig. 11 on the same data set. The superior extent of the heart has been interpreted as the 1st section on which the right and left pulmonary arteries have separated. Throughout, the heart is outlined to the extent of the pericardial sac. The inferior extent is less problematic to delineate as the organ appears well defined compared to the surrounding tissues in the abdomen however if possible the inferior vena cava should be excluded.'

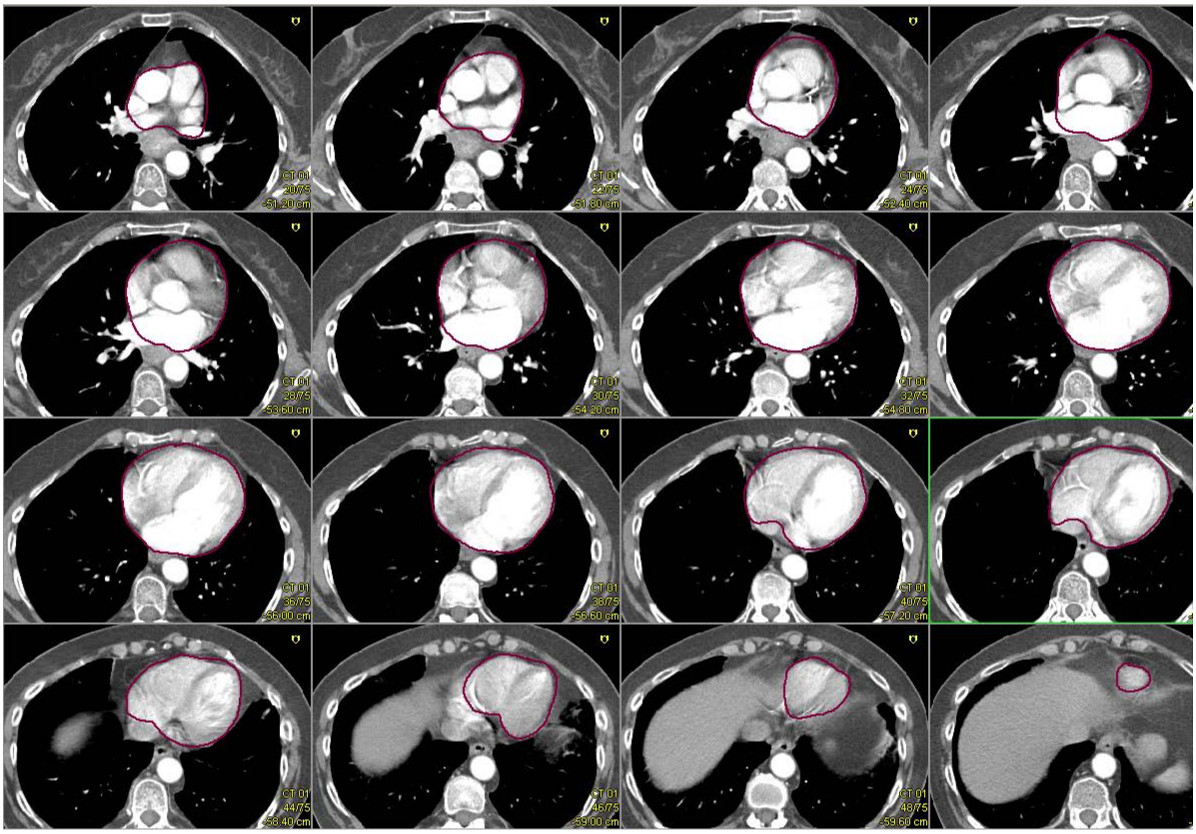

Fig. 11: Alternate 0.3 cm CT slices indicating heart delineation

4 TREATMENT PLANNING

All computer planning must be carried out on a 3D dataset, and correction for tissue heterogeneity must be applied. Usually, a tangential pair beam arrangement is used to encompass the whole breast PTV, minimising the ipsilateral lung and heart in the fields. The treatment plan must be optimised with 3D dose compensation aiming to fulfil the criteria in Tables 2 and 3 below.

4.1 Dose Prescription

The trial schema for the FAST-FORWARD trial is presented on Fig. 12.

Control Group	Test Group 1	Test Group 2
40.05 Gy in 15 Fr<br>3 weeks<br>2.67 Gy/F	27.0 Gy in 5 Fr<br>1 week<br>5.4 Gy/F	26.0 Gy in 5 Fr<br>1 week<br>5.2 Gy/F

16 Gy or 10 Gy in 2 Gy fractions sequential electron or photon boost to the tumour bed is allowed in all 3 treatment groups (boost decision to be declared before randomisation for each individual patient)

Fig.12: FAST-FORWARD trial schema

4.2 Dose Targets and Constraints

4.2.1 Target Volumes

A clinically relevant normalisation point should be used to prescribe the dose to the tangents. Seek QA advice for inverse planned.

The whole breast or chest wall plan should be optimised aiming to fulfil the criteria specified in Tables 2 and 3 below.

Table 2: Upper and lower dose limits for whole breast PTV

	**Mandatory**	**Optimal**
**Lower limit**	V95% ≥ 90%	V95% ≥ 95%
**Upper limit**	V105% ≤ 7%	V105% ≤ 5%
	V107% ≤ 2%	
	D_{max} ≤ 110%	

The V95% and V105% dose objectives have been divided into mandatory and optimal levels.

Exceptions to the lower dose limit may be accepted if all of the following conditions are met:

- All methods for target dose coverage improvement (field-in-field modulation, mixed energies, MLC-shaped segments, longitudinal wedges, etc.) have been applied within reasonable limits;
- More than 90% of the target receives at least 95% of the prescribed dose;
- Coverage to the target is compromised in non-breast tissue or in tissue that is not clinically important;
- The area of the tumour bed is completely covered by the 95% isodose;
- The clinical oncologist has been notified and has accepted the plan.

Every effort should be made to achieve the dose objectives specified above. However, if planning takes more than 1.5 times the amount of time required for routine breast planning and the objectives are still not met, please contact the QA team for advice.

In case the constraints have not been met the reasons for this should be stated in the Comments box on the Plan assessment form.

4.2.2 Dose Constraints for Organs at Risk

The dose constraints for whole breast radiotherapy using tangential field arrangements are listed below. If non-tangential fields are used, e.g. inverse planned IMRT for patients, then the participating centre must seek advice of the QA team.

These constraints do not take into account the tumour bed boost dose.

Control group

- The volume of ipsilateral lung receiving 12.0 Gy should be less than 15%
- The volume of heart receiving 2.0 Gy and 10.0 Gy should be less than 30% and 5% respectively.

Test group 1 and 2

- The volume of ipsilateral lung receiving 8.0 Gy should be less than 15%
- The volume of heart receiving 1.5 Gy and 7.0 Gy should be less than 30% and 5% respectively.

The dose constraints for the organs at risk for whole breast irradiation are summarised in Table 3. In case of photon planned tumour bed boost the local centre policy may be applied. This should be sent to the QA team and must be adhered to for all

patients regardless of trial group. Alternatively, the QA team can provide some guidelines.

Table 3: Dose constraints for organs at risk for whole breast and chest wall irradiation

	**Mandatory**	**Optimal**
**Ipsilateral lung**	V30% ≤ 17%	V30% ≤ 15%
**Heart**	V25% ≤ 5%	
	V5% ≤ 25%	

Although it is essentially a clinical decision, the dose constraints for the organs at risk are generally prioritised over the target coverage constraints. Planners should aim to reduce the amount of lung and heart in the treated area by using MLC shielding on the main treatment fields and/or by shifting the field borders as appropriate, taking into consideration the position of the tumour bed.

4.3 Bolus Use

Centres are encouraged to use bolus to cover part or the entire area of the chest wall for all fractions. If bolus is applied, it should be indicated whether it is to be applied a) to part (e.g. the scar area) or all of the chest wall, b) for all or a specified number of fractions and c) thickness of bolus used for a given photon energy. Bolus can be used in chest wall treatment but not after breast conservation surgery. Bolus policy should be the same for all trial groups.

4.4 Whole Breast and Chest Wall

4.4.1 Photons

Treatment of whole breast and chest wall patients should preferably be done with high-energy photons. Beam energies for treatment as per local practice, usually 6 MV, but a mixture of energies e.g. 6 MV and 15 MV can be used for larger patients.

Treatment planning requirements for each of the 3 groups of the trial may be satisfied with a variety of beam arrangements, however for ease of patient set-up and treatment delivery the following coplanar forward-planning technique is recommended.

The plan consists of two standard tangential fields with non-divergent posterior field edges (Fig. 13 and 14). The isocentre can be placed at the centre of PTV_{WB} or on the slice which falls in the centre of the tumour bed, either on the posterior border or in the centre of the tumour bed volume. The field sizes are selected to cover PTV_{WB} with

collimator rotation to minimise the irradiated lung volume. MLC can be used to shield the ipsilateral lung and heart where possible without compromising PTV coverage.

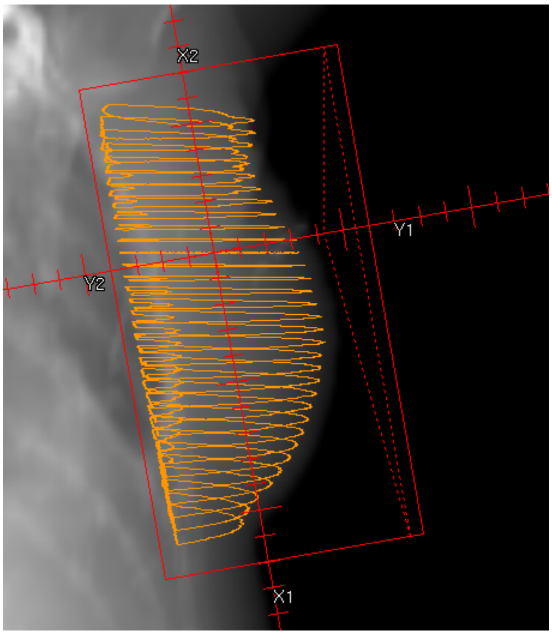

Figure 13: Beam's eye view of whole breast tangent

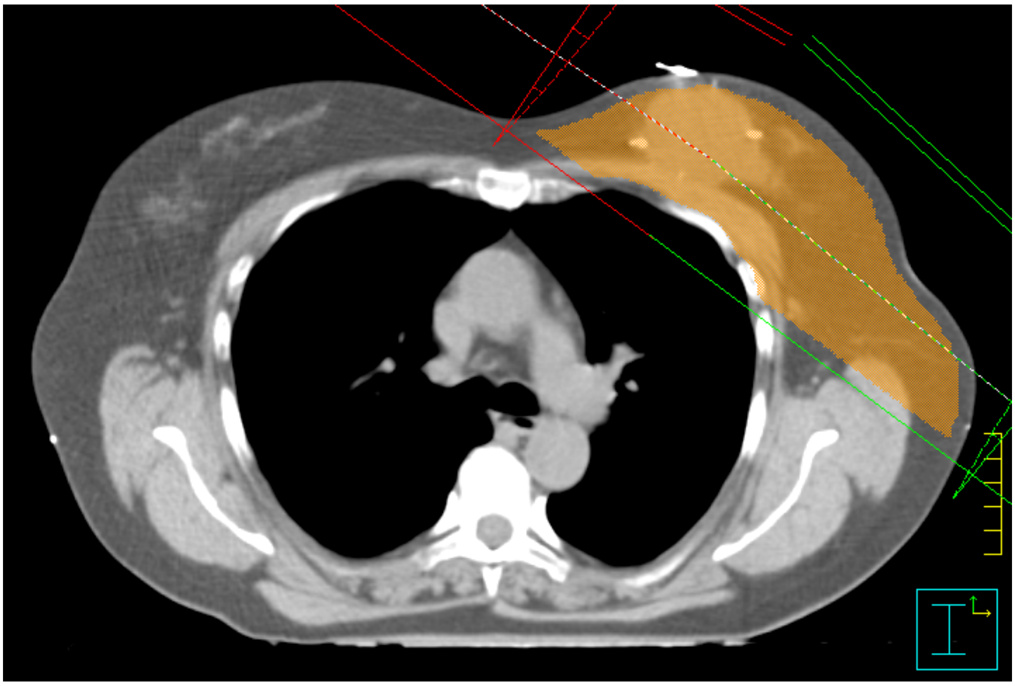

Fig. 14: Whole breast tangential field arrangement

A combination of wedges, simple field-in-field modulation, mixed energies or electronic compensation with step-and-shoot MLC can be used to create a

homogeneous dose distribution to $PTV_{WB/CW}$. Planners should aim to achieve PTV coverage of between 95% and 107% of the prescribed dose.

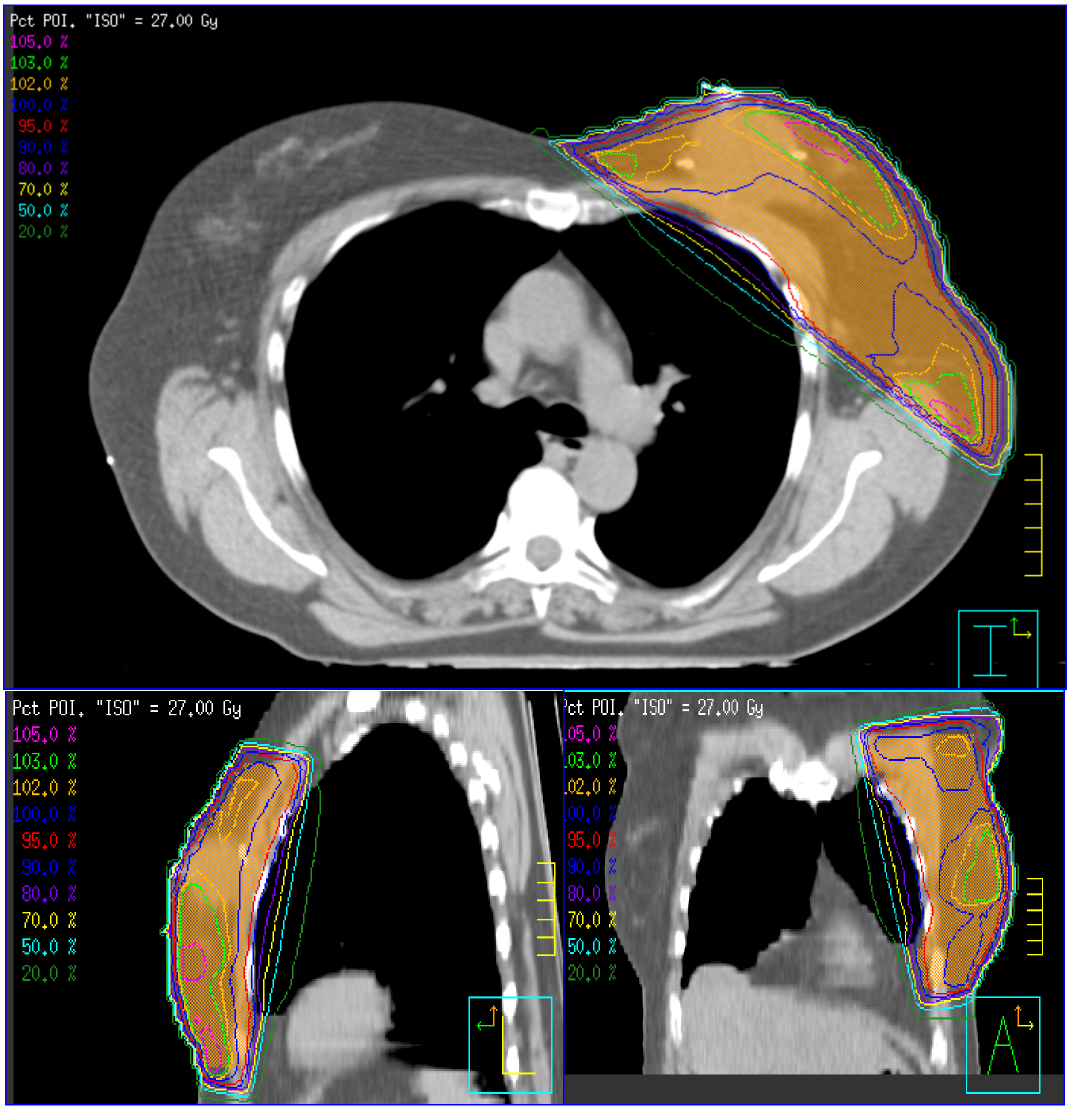

Fig. 15: Dose distribution in whole breast PTV: axial, sagittal and coronal view

On Fig. 16-b) below a projection of the dose clouds in the beam's eye view shows that using simple 3D compensation can minimise hotspots across the breast volume. The image on a) illustrates that using wedged tangents optimised on the central axis with no compensation can produce very large areas of 107% (shown in lavender dose cloud) away from the central axis.

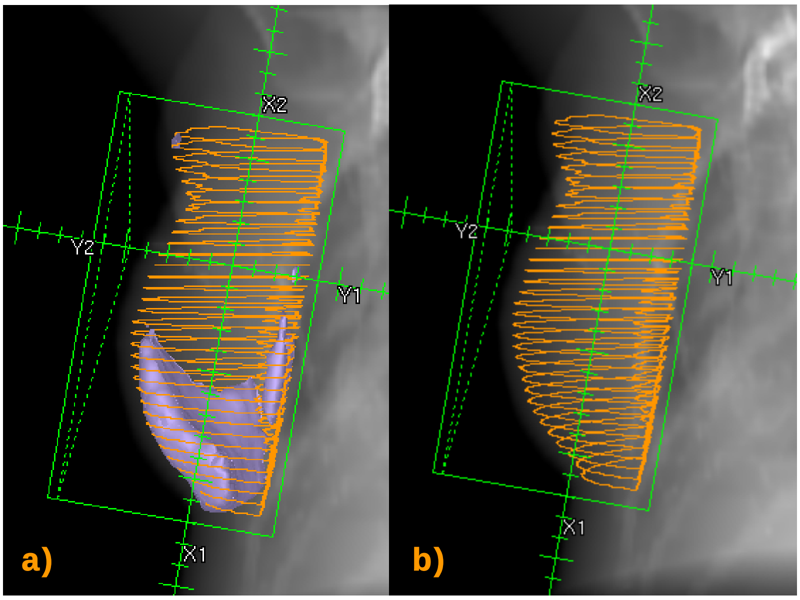

Fig. 16: 3D compensation technique

4.4.2 Electrons

If a centre wishes to treat chest wall patients with electrons, this should be discussed with the QA team.

4.5 Tumour Bed Boost

4.5.1 Photons

Mini-tangential fields can be used for the tumour bed boost, covering the tumour bed PTV with an appropriate penumbra margin. The whole breast field borders are decreased so as to include the tumour bed PTV only. A mini-tangential beam arrangement for the tumour bed boost is presented on Fig. 17.

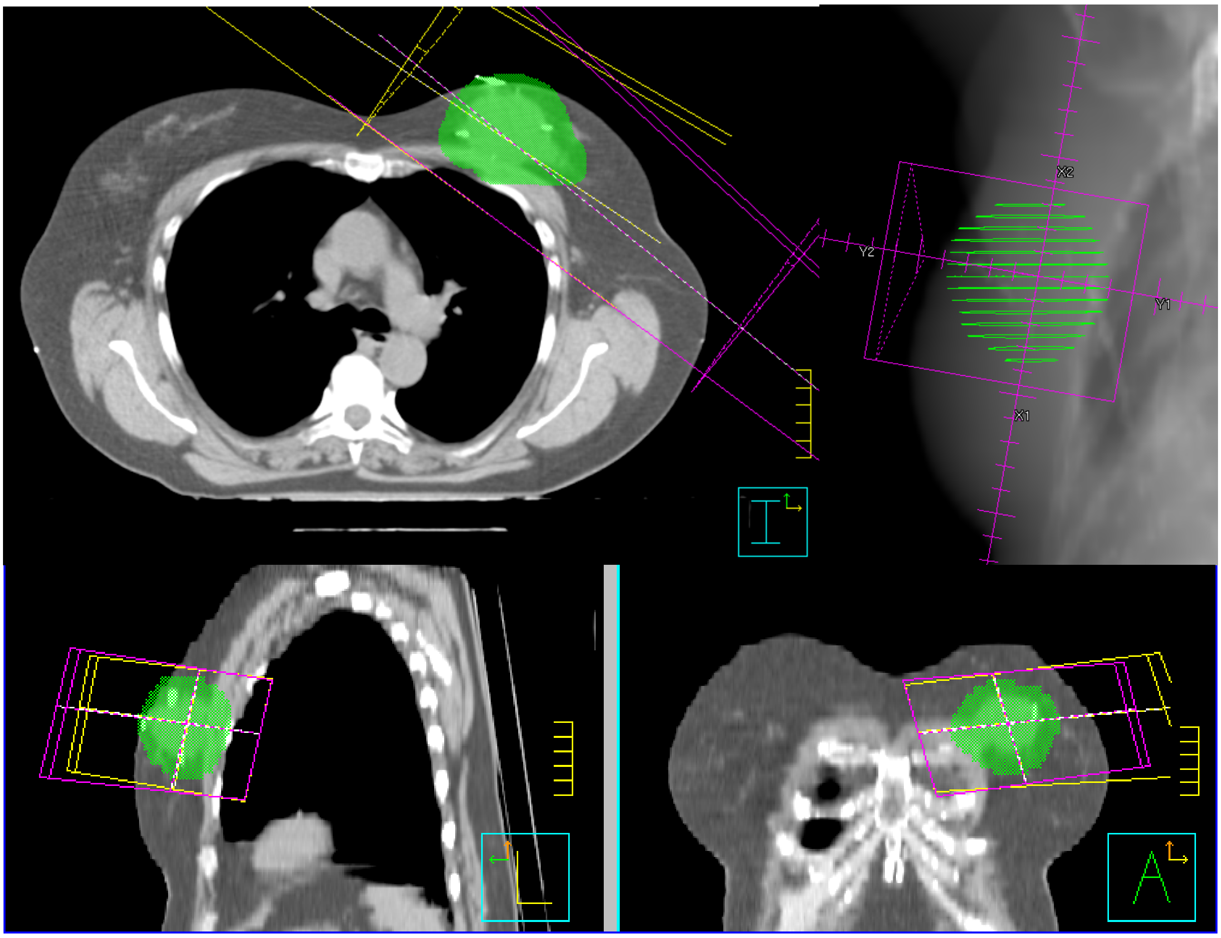

Fig. 17: Image of TB with mini-tangential beams

An alternative field arrangement for the photon boost is a coplanar geometry consisting of 3 to 5 medial and lateral oblique fields. Gantry angles are selected between the whole breast tangents aiming to give 10 Gy or 16 Gy to the tumour bed PTV. The lateral posterior beam(s) will have a shallower gantry angle than the conventional tangent to spare breast tissue, but care should be taken not to exit through the contralateral breast. Extra care should be taken with the lateral anterior beam to ensure the field does not exit through the heart. In the example below 3 beams are added to cover the tumour bed PTV with no margin for penumbra.

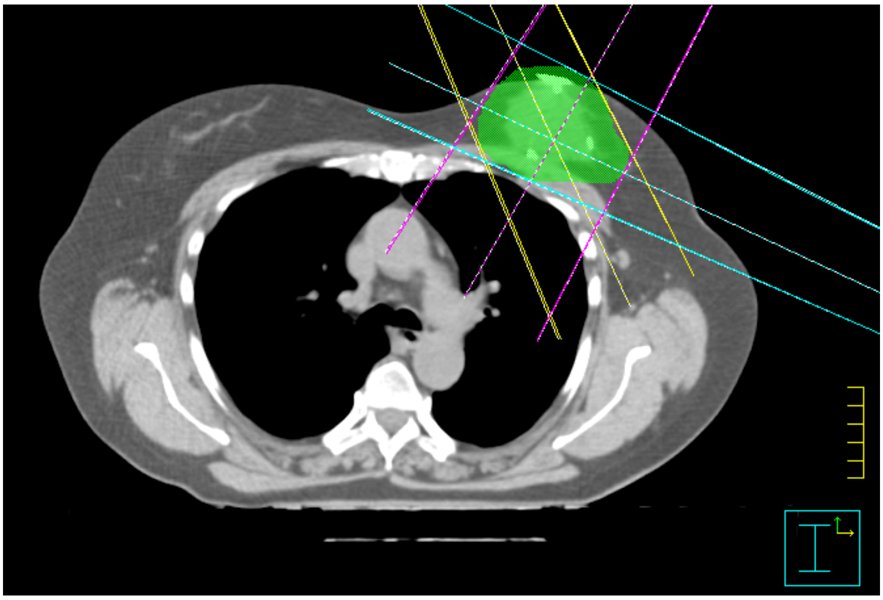

Fig. 18: Tumour bed field placement – coplanar technique

4.5.2 Electrons

Tumour bed boost with electrons can be planned using tabulated depth dose data. Centres should make sure that the beam size, shape, energy and gantry angle are based on the full CT data. The depth of the tumour bed should be used to determine the most appropriate electron energy. The size and shape of the electron aperture should be consistent with the PTV_{TB} contoured in the treatment planning system. Dose should be prescribed to the 100% isodose line, aiming to cover the tumour bed PTV with 90% isodose.

If a centre has the availability to use Monte Carlo or similar algorithm to plan electron boost in the treatment planning system, TPS calculated dose distributions can also be used.

5 RADIOTHERAPY TREATMENT VERIFICATION

5.1 Set-up Verification – Breast and Chest Wall

Verification is carried out using electronic portal imaging of the treatment beam. This can be either MV or kV. The following verification methods are proposed for the control and test groups.

5.1.1 Control Group

Treatment verification is required for the first three fractions in the first week of treatment to determine, and correct for any systematic error. All systematic errors should be corrected, and this is recommended as best practice. If a centre wishes to use a correction tolerance on systematic error it should not be greater than 5 mm, and preferable not more than 3 mm, and reported to the QA team. Correction is carried out following local practice as long as this has been approved by the QA team.

Any correction is applied on fraction 4, with imaging to confirm the move. A suitable tolerance for the check of the correction is 5 mm. Verification is then done once weekly throughout the remaining whole breast field treatment with a tolerance of 5 mm.

5.1.2 Test group 1 and 2

Verification imaging is required for each fraction to check for a gross error prior to treatment. A tolerance of not more than 5 mm should be used. Local policy is followed if the check is out of tolerance. A further image may be taken to confirm any correction, and this also applies where daily imaging is used to correct couch position before treatment. Best practice is to correct all measured displacements.

If MV tangential fields are used, the method to derive the couch correction from the images follows local practice, as long as this has been approved by the QA team.

5.2 Set-up Verification – Tumour Bed Boost

5.2.1 Photon Boost

If a conformal photon boost is used, it is recommended to use one of the verification protocols below for both fractionation options (10 Gy in 5 fractions or 16 Gy in 8 fractions).

i) an on-line verification protocol which corrects for both systematic and random errors in patient set-up but has a time and dose penalty as it requires daily imaging and correction

ii) an off-line (eNAL or NAL eNAL protocol of de Boer) correction protocol which only corrects for the systematic error but has the advantage of a reduced imaging burden as daily imaging is not required.

If photon mini-tangent fields are used, the first 2 or 3 fractions are imaged (as appropriate for the fractionation scheme). A correction for the systematic error is made for the remaining fractions. It is recommended that all systematic errors are corrected. If a centre wishes to use a correction tolerance on systematic error it should not be greater than 5mm, and preferable not more than 3 mm, and reported to the QA team. A check of the correction may be made on fraction 6 for the 16 Gy in 8 fraction schedule; 5 mm is a suitable tolerance.

Alternatively, as the fractionation schedules are short, daily imaging may be used as described for the whole breast/chest wall Test Groups 1 and 2.

5.2.2 Electron Boost

The electron boost set up is verified daily by visual matching to marks on the skin and checks on the gantry and collimator angles required for matching. If virtual simulation is used, the gantry and collimator angles should not vary by more than 5° from the values identified as most suitable during simulation.

Where the need for more complex treatment planning (e.g. inverse planning or tomotherapy) requires a verification method not described here, centres are requested to discuss this on an individual basis with the QA Team. Similarly, if a centre wishes to use a tighter PTV margin with a more stringent verification protocol, this should be discussed with the QA Team.

5.3 In-vivo Dosimetry

In line with current UK guidelines, it is strongly recommended that all FAST-Forward patients have in-vivo dosimetry within the first week of treatment for the control group, and on the first day for the test groups. This may be performed using diodes or thermo-luminescent dosimetry (TLD).

6 TREATMENT GAPS

A gap of up to 3 days is acceptable in the event of machine service, breakdown or patient illness. If the treatment machine is unavailable for more than 3 days, or if you wish to move a patient to a machine with no imaging facilities before the initial correction for systematic error has taken place, please contact the QA team.

7 REFERENCES

1. Surgical guidelines for the management of breast cancer. Eur J Surg Oncol, 2009. 35 Suppl 1: p. 1-22.
